# HIV Tat‐Stimulated Microglial Extracellular Vesicles Are Enriched for Ferroptosis Mediators: Role of Dysregulated Autophagy

**DOI:** 10.1002/jex2.70153

**Published:** 2026-06-11

**Authors:** Seema Singh, Elias Horanieh, Craig L. Semerad, Palsamy Periyasamy, Susmita Sil, Shilpa Buch

**Affiliations:** ^1^ Department of Pharmacology and Experimental Neuroscience University of Nebraska Medical Center Omaha Nebraska USA; ^2^ Flow Cytometry Research Facility University of Nebraska Medical Center Omaha Nebraska USA

**Keywords:** autophagy, extracellular vesicles, ferroptosis, HIV Tat, MEVs

## Abstract

Extracellular vesicles (EVs) serve as conduits for intercellular communication, under both physiological and pathological contexts. During disease pathogenesis, microglia‐derived EVs (MEVs) play a key role in transferring pathological cargo to the recipient cells, thereby modulating their phenotype and function. The current study was aimed to explore how HIV Transactivator of transcription (Tat) protein‐mediated dysregulated autophagy/ferritinophagy contributes to the expression and release of ferroptotic mediators in Tat‐activated MEVs. Exposure of microglial BV2 cells to HIV Tat for 48 h resulted in significantly increased expression of the ferroptosis mediators—FTH1 and ACSL4 in Tat‐MEVs. Reciprocally, pretreatment of microglia with the ferroptosis inhibitors abrogated the expression and release of these mediators in both microglial cells as well as in Tat‐MEVs, without affecting the overall MEV release. Furthermore, inhibition of autophagic flux with bafilomycin A1 resulted in potentiation of HIV Tat‐mediated expression and release of ferroptosis mediators in Tat‐MEVs. In contrast, treatment of cells with the autophagy inducer‐ rapamycin, attenuated these effects. Exposure of BV2 cells to HIV Tat also resulted in increased expression of the ferritinophagy adaptor protein NCOA4, an effect mitigated by both ferroptosis inhibitors and rapamycin. These findings were also validated in brain lysates as well as brain‐derived MEVs isolated from HIV Transgenic rats. Collectively, this study highlights the role of dysregulated autophagy in modulating both HIV Tat‐induced ferroptosis and the release of ferroptosis cargo in MEVs. These findings advance our understanding of the molecular mechanism(s) driving HIV‐associated neuropathology.

## 1 Introduction

As of 2023, Human immunodeficiency virus‐1 (HIV‐1) infection remains a major global health burden, with approximately 40 million people living with HIV‐1. While the advent of combined antiretroviral therapy (cART) has markedly reduced the mortality and substantially extended the lifespan of people living with HIV (PLWH), neurological complications, collectively termed NeuroHIV, continue to afflict infected individuals. Despite effective suppression of peripheral viremia by cART, the prevalence of cognitive impairment in PLWH remains high, affecting approximately 50%–70% of individuals (Zenebe et al. 2022), suggesting that mechanism(s) beyond active systemic replication contribute to disease progression, including low‐level viral activity and the continued presence of viral proteins such as HIV Tat (Henderson et al. 2019) within the central nervous system (CNS).

Once inside the cells, HIV Tat accumulates within the endolysosomal compartments, leading to lysosomal deacidification (Khan et al. 2022) and promoting the release of redox‐active iron into the cytosol. Elevated cytosolic iron is a well‐established driver of lipid peroxidation and ferroptotic cell death (Tang et al. 2021). Recent findings from our lab have demonstrated the role of miR‐204‐ACSL4 signaling axis in HIV Tat‐mediated microglial ferroptosis (Kannan et al. 2023), further supporting the role of lipid remodeling in ferroptotic susceptibility. In addition to iron release from endolysosomal compartments, lysosomes play a central role in regulating intracellular iron homeostasis through ferritinophagy, a selective autophagic process that mediates the degradation of ferritin heavy chain 1 (FTH1‐ one of the ferroptosis mediators). Disruption of lysosomal function can thus impair ferritinophagy, leading to intracellular buildup of FTH1, either in the cytosol or sequestered in the autophagosomes (Chen 2026). Since lysosomal and autophagic pathways are closely integrated with vesicular trafficking, disturbances in these processes could likely alter the composition and release of extracellular vesicles (EVs), thereby enabling the transmission of ferroptosis‐related signals beyond the affected cell. Furthermore, emerging evidence suggests that ferroptosis‐associated mediators, including lipid peroxidation products and iron regulatory molecules, can be packaged into and transferred through EVs, potentially influencing neighboring cells within the brain microenvironment (Huang et al. 2026; Ito et al. 2021).

EVs are nano‐sized, membrane‐enclosed particles released into the extracellular space by nearly all cell types and have been implicated as key mediators of intercellular communication, both in normal cellular physiology and in disease pathogenesis (Dixson et al. 2023; Hill 2019; Takeuchi 2021). Previous findings by us (Sil et al. 2021; Singh et al. 2023; Thangaraj et al. 2018) and others (Kyei et al. 2009) have shown that HIV‐Tat disrupts autophagy in both microglia and astrocytes. Taken together, we thus hypothesized that HIV Tat‐mediated impairment of the autophagy‐lysosomal axis contributes to the induction and release of ferroptosis‐associated cargo in Tat‐microglia‐derived EVs (MEVs). Our results demonstrated that HIV Tat significantly increases the release of MEVs, which are enriched in ferroptotic mediators, including Acyl‐CoA synthetase long‐chain family member 4 (ACSL4) and FTH1. Interestingly, bafilomycin‐A1 (BAF‐A1), a lysosomal inhibitor, further enhanced the release of Tat‐MEVs carrying ferroptosis‐related cargo, an effect that was attenuated by autophagy‐inducing agent, rapamycin (Rap). Together these findings underscore a critical role for the autophagy‐lysosomal pathway, and specifically dysregulated ferritinophagy in regulating the release of HIV Tat MEVs enriched in ferroptosis‐associated mediators. These observations were also validated in brain tissue and brain‐derived microglial EVs isolated from HIV Transgenic (Tg) rats.

## 2 Materials and Methods

### 2.1 Reagents and Antibodies

Endotoxin‐free, HIV recombinant Tat‐101 protein (Cat No. 1032–10) was purchased from ImmunoDiagnostics Inc., Woburn, MA, USA. Based on the manufacturer's specifications, endotoxin levels in the recombinant Tat protein were <0.01 EU/mg of protein, as determined using the BioWhittaker Kinetic QCL assay. The Tat protein was purified by ion‐affinity chromatography followed by reverse‐phase HPLC to >95% purity, as verified by SDS‐PAGE and HPLC analysis. GW4869 (Cat No. D1692), Deferoxamine (DFO; Cat No. D9533), ferrostatin‐1 (Fer‐1; Cat No. SML0583), Bafilomycin A1 (BAF‐A1; Cat No. B1793), Rapamycin (Rap; Cat No. and 553210), and Anti‐β‐actin (Cat No. A5316) were obtained from Sigma‐Aldrich, St. Louis, MO, USA. Anti‐LC3B (Cat No. NB100‐2220), Anti‐lysosome‐associated membrane protein 2 (LAMP2; Cat No. NB300‐591), and Anti‐ferritin heavy chain 1 (FTH1; Cat No. MAB9354) were sourced from Novus Biological Company, Centennial, CO, USA. Anti‐NCOA4 (Cat No. sc‐373739), horseradish peroxidase (HRP)‐conjugated goat Anti‐rabbit (Cat No. sc‐2004), Anti‐GPX4 (sc‐166570), and HRP‐conjugated goat Anti‐mouse (Cat No. sc‐2005) were purchased from Santa Cruz Biotechnology, Inc., Dallas, TX, USA. SQSTM1 (Cat No. PM045) was acquired from MBL International, Woburn, MA, USA. Anti‐CD63 (Cat No. ab216130), Anti‐CD9 (Cat No. ab92726), Anti‐TSG101 (Cat No. ab125011), Anti‐Alix (Cat No. ab275377), Anti‐Calnexin (Cat No. ab133615), Anti‐FTH1 (Cat No. ab75973), and Anti‐ACSL4/FACL4 (Cat No. ab205199) were purchased from Abcam, Boston, MA, USA. Triton X‐100 (Cat No. BP151), Alexa Fluor donkey anti‐goat 594 (Cat No. A11058), Alexa Fluor donkey anti‐rabbit 488 (Cat No. A21206), Alexa Fluor donkey anti‐mouse 647 (Cat No. A31571), Invitrogen ProLong^TM^ Gold antifade reagent with DAPI (Cat No. P36935), Alexa Fluor goat anti‐rabbit 488 (Cat No. A11008), and Alexa Fluor goat anti‐mouse 594 (Cat No. A11005) were obtained from Invitrogen, Thermo Fisher Scientific, Waltham, MA, USA. Antigen unmasking solution (citric acid‐based, Cat No. H‐3300) and goat serum (Cat No. S‐1000) were purchased from Vector Laboratories, Newark, CA, USA. Donkey serum (Cat No. 017‐000‐121) was purchased from Jackson Immuno Research Laboratories, West Grove, PA, USA.

### 2.2 Animals

Sixteen to eighteen‐month‐old HIV Transgenic (Tg; 3 male and 3 female) and age‐matched wildtype (WT; 2 male and 4 female) rats were used in this study (*n* = 6). The rats were housed under controlled temperature and humidity conditions with a 12‐h light/dark cycle (7:00 AM to 7:00 PM). Food and water were provided *ad libitum*. All experimental procedures were approved by the Institutional Animal Care and Use Committee at the University of Nebraska Medical Center and were conducted in accordance with the guidelines of the National Institutes of Health. The HIV transgenic rat model used in this study expresses a non‐replicating HIV‐1 provirus containing 7 of the 9 viral genes, with functional deletions in the gag and pol regions, enabling the expression of multiple viral proteins, including HIV Tat. This model exhibits sustained, low‐level expression of HIV‐1 proteins across tissues, including the CNS, and has been extensively used for the study of HIV‐associated neuropathology (Peng et al. 2010; Reid et al. 2001; Vigorito et al. 2015).

### 2.3 BV2 Cell Culture

The mouse BV2 cell line was generously provided by Dr. Sanjay Maggirwar from the George Washington School of Medicine and Health Sciences, Washington, D.C., USA. Cells were cultured in Dulbecco's Modified Eagle's Medium (DMEM) supplemented with 10% heat‐inactivated fetal bovine serum (FBS; Thermo Fisher Scientific, Waltham, MA, USA, Cat No. 16000–044) and penicillin‐streptomycin solution (5 mL; 10,000 Units/mL Penicillin, 10,000 µg/mL Streptomycin; Life Technologies Corporation, Grand Island, NY, USA, Cat No. 15140‐122). BV2 cells were seeded at a density of 0.15 × 10^6^ cells per well in a six‐well plate, 0.05 × 10^6^ cells per coverslip in a 24‐well plate, or 1.5 × 10^6^ cells per T150 flask, depending on the experimental requirements. The cells were grown in a humidified incubator with 5% CO_2_ at 37°C. Upon reaching 70% confluency, the cells were subjected to overnight starvation in serum free DMEM followed by exposure to HIV Tat protein (100 ng/mL) in serum free DMEM for 48 h. GW4869 (5 µM) was administered 30 min prior to exposing cells to HIV Tat. BV2 cells were pretreated with either DFO (2 µM), Fer‐1 (10 µM), or Rap (100 nM) 1 h prior to HIV Tat protein. BAF‐A1 was added 4 h prior to the end of the experiment. Control cells were treated with DMSO (final concentration 0.001%) and using the same experimental conditions as those for the inhibitors. Additionally, heated‐Tat was prepared from the same original stock of Tat (final concentration 100 ng/mL).

### 2.4 Western Blotting

Protein expression in the samples was assessed using western blotting. After the experiments, BV2 cells were rinsed with 1x Phosphate Buffered Saline (PBS) and lysed in 200 µL of RIPA buffer. For tissue samples, 500 µL of RIPA buffer was used to lyse brain tissues (10–15 mg) from HIV Tg and WT rats. Protein concentrations were determined using the Pierce BCA Protein Assay Kit (Thermo Fisher Scientific, Waltham, MA, USA, Cat No. 23227) according to the manufacturer's instructions. Total proteins in cell lysates (equal protein amounts) as well as in EVs (equal EV numbers) were separated by molecular weight using sodium dodecyl sulfate‐polyacrylamide gel electrophoresis (SDS‐PAGE). Following separation, proteins were transferred onto a polyvinylidene difluoride (PVDF) membrane (Millipore Sigma, MO, USA, Cat No. IPVH00010). To reduce nonspecific binding, the membrane was blocked with 5% nonfat skimmed milk for 1 h (Research Products International, PA, USA, Cat No. M17200‐1000.0). After blocking, the membrane was washed and then incubated overnight with primary antibodies specific to the target proteins of interest. The membrane was subsequently washed to remove unbound antibodies and incubated with HRP‐conjugated secondary antibodies (anti‐mouse or anti‐rabbit IgGs). The detection of protein bands was performed using SuperSignal chemiluminescent substrate (Thermo Fisher Scientific, Waltham, MA, USA, Cat No. VJ311133), which reacts with HRP to produce a chemiluminescent signal. This signal was captured using an imaging system (Fluorchem M, Cell Biosciences, Santa Clara, CA, USA), and β‐actin was used as a loading control. Densitometry analysis was performed to quantify the intensity of protein bands using ImageJ software (Schneider et al. 2012).

### 2.5 Immunocytochemistry

After completing the experiments, BV2 cells were rinsed twice with 1x PBS at room temperature (RT) to eliminate any remaining culture medium. The cells were then fixed using 4% paraformaldehyde at RT for 20 min to maintain cellular architecture. Following fixation, the cells were permeabilized with 0.1% Triton X‐100 for 15 min at RT, which allowed the antibodies to penetrate and bind to intracellular targets. To minimize nonspecific antibody binding, the cells were subsequently blocked with 10% normal goat serum (Vector laboratory, Newark, CA, USA, Cat. No. S‐1000‐20). The cells were then incubated overnight at 4°C with primary antibodies specific to FTH1 (1:500) and NCOA4 (1:500). After overnight incubation, cells were washed three times with 1x PBS to remove unbound primary antibodies followed by incubation with fluorescent‐conjugated secondary antibodies—goat anti‐rabbit (1:1000) and goat anti‐mouse (1:1000)—for 1 h at RT to enable visualization of the targeted proteins. Following this, the cells were thoroughly washed to remove any unbound secondary antibodies and subsequently mounted with DAPI to stain the nuclei. Images of the stained cells were captured using a Z1 inverted microscope (Carl Zeiss, Thornwood, NY) and analyzed with AxioVs 40 Version 4.8.0.0 software (Carl Zeiss MicroImaging GmbH). Quantitative analysis of the protein expression was performed using ImageJ software (Schneider et al. 2012).

### 2.6 Immunohistochemistry

Immunohistochemistry was conducted on formaldehyde‐fixed, paraffin‐embedded tissue sections (40 µm thick) obtained from the frontal cortex of HIV Tg and wildtype control rats. Initially, the tissue sections were deparaffinized by immersion in xylene and then rehydrated through a descending ethanol series (100%, 70%, and 50%) followed by rinsing with 1 x PBS. Antigen retrieval was performed using a citrate‐EDTA buffer (pH 6.0) at 100°C to expose the epitopes. Following antigen retrieval, the sections were blocked with 10% normal donkey serum (Jackson ImmunoResearch, West Grove, PA, USA, Cat. No. 017‐000‐121) to minimize nonspecific binding. The tissues were then incubated overnight at 4°C with primary antibodies targeting FTH1 (1:250), NCOA4 (1:250), and IBA1 (1:250) to ensure specific binding to the target antigens. After removing excess primary antibodies by washing with 1X PBS, the sections were incubated with corresponding fluorescent‐conjugated secondary antibodies for 2 h at RT. Subsequent washes were performed to eliminate unbound secondary antibodies. The tissues were then mounted with DAPI‐containing ProLong Gold antifade reagent. Imaging was performed using a Z1 inverted microscope (Carl Zeiss, Thornwood, NY), with consistent exposure settings applied across all samples to facilitate accurate comparisons. The captured images were analyzed using AxioVs 40 Version 4.8.0.0 software (Carl Zeiss MicroImaging GmbH), and protein expression levels were quantified using ImageJ software (Schneider et al. 2012).

### 2.7 EV Isolation From BV2 Microglia

EVs were isolated from BV2 cell‐conditioned media using a differential centrifugation (DUC) approach as previously described by us (Kannan et al. 2022). DUC is a widely used and well‐established method for EV isolation, allowing efficient processing of relatively large sample volumes (60 mL conditioned media for each replicate) while also maintaining compatibility with downstream functional and molecular analyses. Briefly, at the end of the experiments, the conditioned media was collected and subjected to an initial centrifugation at 300 × *g* for 10 min to remove cellular debris. This was followed by another centrifugation at 2000 × *g* for 10 min to eliminate larger apoptotic bodies and dead cells. The resulting supernatants were then centrifuged at 10,000 × *g* for 30 min to remove larger vesicles and organelles. To isolate small EVs, the supernatants were passed through 0.22 µm filters to remove any remaining large particles and subsequently ultracentrifuged at 100,000 × *g* for 70 min using a Beckman SW 32 Ti rotor (Beckman Coulter, Brea, CA, USA). All centrifugation steps were conducted at 4°C to maintain EV integrity. The purified EVs were resuspended in 200 µL of 0.22 µm filtered PBS for further characterization and downstream analyses. EV characterization was performed using a nanoparticle tracking analyzer (NTA) using the ZetaView instrument (Particle Metrix, Ammersee, Bavaria, Germany), as previously detailed (Kannan et al. 2022; Sil et al. 2021). Additionally, EV morphology was assessed using transmission electron microscopy (TEM), and the presence of EV‐specific markers, such as CD63, CD9, ALIX, and TSG101, was confirmed by western blotting. Protein concentration was quantified using the BCA protein assay kit (Pierce, Rockford, IL, USA).

### 2.8 Isolation of EVs From Rat Brain

The protocol used for brain EV isolation in this study is well established and has been described in previous publications from our group (Dagur et al. 2020; Sil et al. 2021). Briefly, brains from HIV Tg and wildtype rats were extracted, weighed, and 1000 mg of brain tissue was homogenized in 10 mL of chilled Hibernate A medium (Thermo Fisher Scientific, Waltham, MA, USA. Cat No. A12475‐01) with papain (20 units/mL, Thermo Fisher Scientific, Waltham, MA, USA, Cat No. 416760100) using the gentleMACS Octo Dissociator (Miltenyi Biotec, Gaithersburg, MD, USA) for 30 min. The reaction was then stopped by adding 20 mL of warm Hibernate A medium. The homogenate was centrifuged at 300 × *g* and 2000 × *g* for 10 min each at 4°C to remove debris. The supernatant was centrifuged at 10,000 × *g* for 30 min at 4°C to isolate larger vesicles. After filtration through 0.22 µm filters, smaller EVs were pelleted by ultracentrifugation at 100,000 × g for 70 min at 4°C. The EV pellet was resuspended in 500 µL of 1x PBS. For further purification, EVs were subjected to iodixanol (Optiprep, Cosmo Bio, Carlsbad, CA, USA. Cat No. 04‐03‐9392/01) gradient centrifugation. Gradients were prepared with 40%, 20%, 10%, and 5% iodixanol layers (2.7 mL each). The EVs were layered onto the gradient and ultracentrifuged at 100,000 × *g* for 18 h at 4°C. Iodixanol density gradient ultracentrifugation was used to isolate EVs based on buoyant density, while also minimizing contaminants and preserving EV integrity. This method was prioritized over size‐exclusion chromatography (SEC) because it provides higher EV yield from brain tissue and enables pooling of EV‐rich fractions (F6–F9) to obtain concentrated, high‐purity vesicle preparations for downstream analyses. In contrast, although SEC is effective for separating EV populations by size, it often generates relatively diluted eluates, which may limit recovery of sufficiently enriched EVs for subsequent experimental applications (Welsh et al. 2024).

Following centrifugation, 0.9 mL fractions (F1‐F12) were collected, washed with 1X PBS by ultracentrifugation at 200,000 × *g* for 3 h at 4°C, and resuspended in 300 µL filtered (0.22 µm) 1X PBS. Aliquots were stored at −80°C for future use. EV preparations were evaluated based on multiple criteria in accordance with the MISEV2023 guidelines (Welsh et al. 2024) to ensure isolation of EV‐rich fractions from brain samples. EV characterization included nanoparticle tracking analysis (NTA) using the Nanosight NS300 system to determine size distribution, TEM analysis to visualize the characteristic cup‐shaped morphology of EVs, and Western blot analysis to confirm the presence of EV‐specific markers (TSG101, CD9, CD63) while assessing potential contaminants such as calnexin. Protein expression levels were normalized to EV numbers, and densitometric quantification was performed used ImageJ with standardized settings, applying identical analysis parameters across all groups (Schneider et al. 2012).

### 2.9 ZetaView Tracking Analysis

EVs isolated from BV2 cells and rat brain tissues were analyzed using the ZetaView NTA (Particle Metrix, Ammersee, Bavaria, Germany) and ZetaView 8.04.02 SP1 software. The instrument was calibrated using 100 nm polystyrene standard particles prior to analysis. EVs were diluted in filtered 1X PBS to achieve optimal particle concentration. Video recordings were made with system settings at a shutter speed of 100, sensitivity of 85, and a frame rate of 30 frames per second. The size distribution (in nanometers) and concentration (particles per milliliter) were determined by injecting the diluted EVs into the ZetaView analyzer, with two cycle readings taken at each position. The total number of EVs per sample was calculated by multiplying the particle count per milliliter by the dilution factor.

### 2.10 TEM

Negative staining of BV2 and Rat brain‐derived EVs (BEVs) was performed with slight modifications to standard protocols. Briefly, 10 µL of EVs suspended in filtered 1X PBS were placed onto 200‐mesh Formvar‐coated copper grids and allowed to adsorb for 5 min. The grids were then stained with uranyl acetate for contrast. After staining, the grids were rinsed with 1X PBS, blotted with filter paper to remove excess liquid, and air‐dried at RT. Imaging was acquired using a Hitachi H7500 electron microscope (Hitachi, Tokyo, Japan) at 200 kV.

### 2.11 Flow Cytometry Analysis of EVs

The EV fractions (pooled F6‐F9, BEVs) were analyzed using a BD Biosciences LSR II. Briefly, ∼ 10 × 10^10^ BEVs were incubated with rat anti‐CD11B (1:50), rabbit anti‐CD63 (1:50), and mouse anti‐FTH1 (1:50) for 48 h at 4°C. Following washing with filtered PBS, BEVs were incubated with corresponding secondary antibodies (1:100) (AF488 goat anti‐rat, AF647 goat anti‐rabbit, and AF594 goat anti‐mouse) at 4°C and then washed with filtered PBS. Samples were analyzed for microglia‐specific EVs. Prior to sample analysis, the Nano Fluorescent Size Standard Kit (100–500 nm Cat No. NFPPS‐52‐4K, Spherotech) was used to help establish PMT voltages, set the threshold, and adjust the scatter gate. UltraComp eBeads Plus Compensation Beads (Cat No. 01‐3333‐42, Thermo Fisher) and single fluorescent‐stained samples were used to perform compensation. Analysis gates were based on unstained EVs, single‐color EVs controls, and fluorescence minus one (FMO) controls. Following initial gating based on scatter parameters, BEVs were sequentially gated for CD63 followed by CD11B expression. The CD63^+^CD11B^+^ subpopulation was subsequently analyzed for FTH1 expression. To minimize swarm detection, BEV samples were serially diluted in 1X filtered PBS (1:1 to 1:4), and events analyzed. Particle numbers were determined within the linear detection range of the instrument. Double‐positive EV events were distinguished from aggregates using fluorescence intensity distribution and forward/side scatter gating. Stringent gating combined with serial dilution ensured detection within the single‐particle range. Post analysis, EV samples were treated with 10% Triton X‐100 to disrupt membrane‐bound vesicles, thereby confirming the detected signals originated from intact vesicular structures. Data was analyzed using FlowJo 10.10.0 (BD Biosciences) and/or BD FACSDiva Software, Version 8.0.2 (BD Biosciences).

### 2.12 Statistical Analysis

Data distribution was evaluated prior to statistical analysis to assess normality using standard approaches available in GraphPad Prism (version 10.4.1, San Diego, CA, USA). In vitro datasets that did not meet normality assumptions were analyzed using non‐parametric methods. Specifically, in vitro data were analyzed using the Kruskal‐Wallis one‐way analysis of variance followed by Dunn's multiple comparisons test to determine differences between groups and control for type I error. For in vivo animal studies involving two‐group comparisons (WT vs. HIV Tg; *N* = 6 per group), an unpaired Student's t‐test was applied where data satisfied parametric assumptions. Sample sizes were determined based on prior literature and our preliminary studies, with an emphasis on detecting biologically meaningful differences and ensuring reproducibility. All experiments were performed using at least six independent biological replicates unless otherwise specified. For rat BEV studies, three independent biological replicates were used. Data are presented as mean ± SEM. All statistical analyses were performed using GraphPad Prism (version 10.4.1, San Diego, CA, USA). Statistical significance was defined as *p* < 0.05.

## 3 Results

### 3.1 Exposure of BV2 Microglia to HIV Tat Increased the Release of Tat‐MEVs With Enriched Ferroptotic Cargoes

**FIGURE 1:**
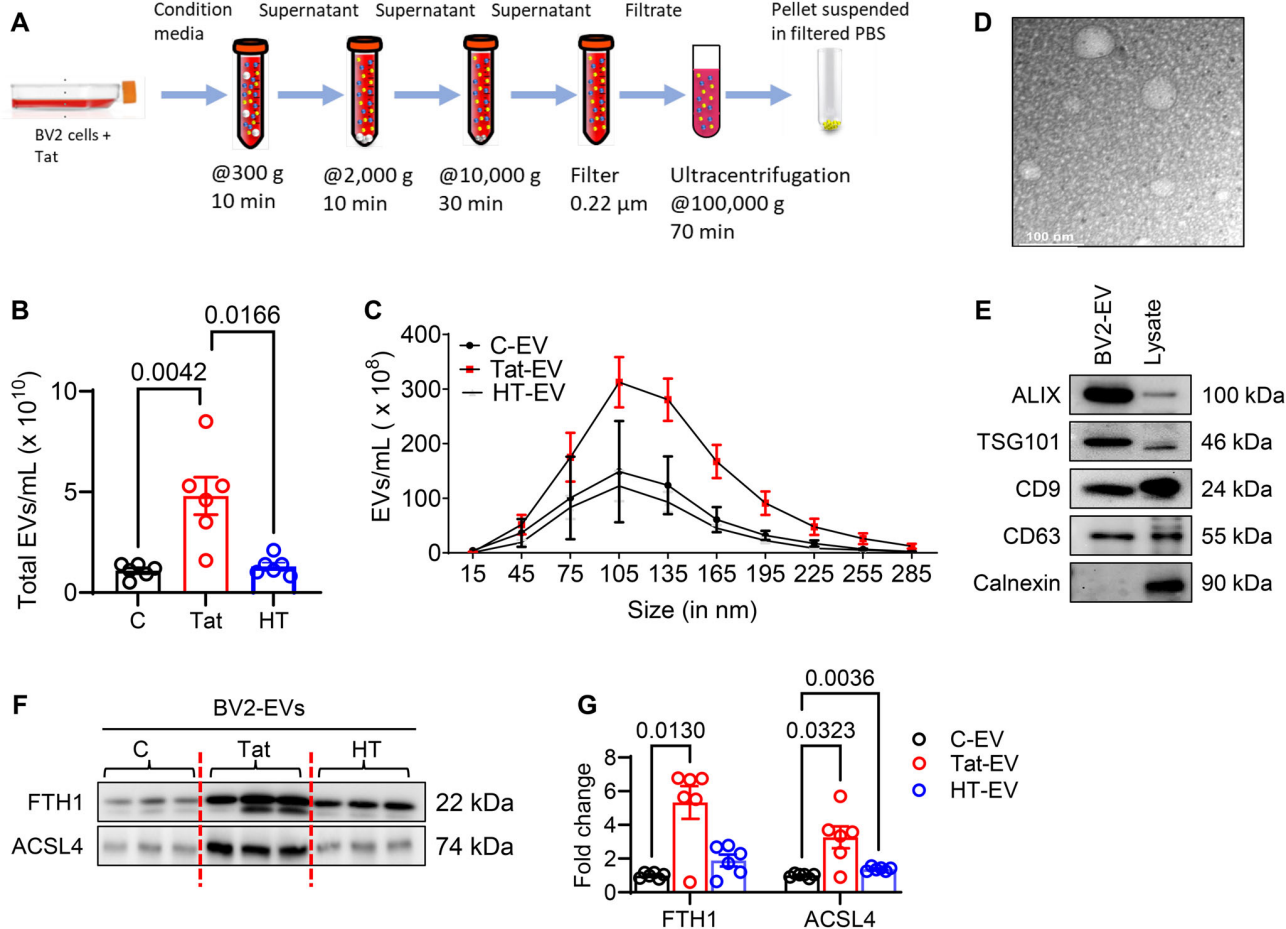
Characterization of BV2‐derived EVs. (A) Schematic illustrating the EV isolation protocol from conditioned media of BV2 cells; (B) Total EV count, and (C) particle size distribution as measured by ZetaView; (D) Representative TEM image of EVs; scale bar: 100 nm; (E) Western blot analysis of EV biomarkers (ALIX, TSG101, CD9, CD63) and non‐EV marker calnexin; (F) Western blot analysis and (G) quantification of ferroptosis markers—FTH1 and ACSL4 in EVs isolated from control, HIV Tat (100 ng/mL), or heat‐inactivated Tat (HT, 100 ng/mL) exposed BV2 cells for 48 h. Data is presented as mean ± SEM from six independent experiments. Abbreviations: HT: heat‐inactivated Tat protein; EV: extracellular vesicles; TEM: transmission electron microscope; FTH1: ferritin heavy chain‐1; ACSL4: acyl‐CoA synthetase long chain family member 4.

Tat‐MEVs were isolated from the conditioned media of HIV Tat‐activated BV2 cells by gradient centrifugation and characterized (Kannan et al. 2022), as depicted in Figure 1A. NTA analysis revealed a significant increase in the total number of particles released by BV2 cells exposed to HIV Tat (4.8 × 10^10^ ± 0.9338 × 10^10^) compared to MEVs released from control cells (1.088 × 10^10^ ± 0.1378 × 10^10^) or in cells exposed to heat‐inactivated HIV Tat (HT; 1.307 × 10^10^ ± 0.1838 × 10^10^) groups (Figure 1B). The size distribution analysis indicated that these particles ranged primarily between 45 and 195 nm in diameter, peaking at 105 nm (Figure 1C). Pretreatment of BV2 cells with GW4869 (2.583 × 10^10^ ± 0.9728 × 10^10^) abrogated the release of EVs from BV2 cells (Figure S1A). TEM studies further confirmed the characteristic cup‐shaped morphology of the EVs (Figure 1D). Western blot analysis revealed that these fractions were enriched in EV markers, including ALIX, TSG101, CD9, and CD63, thereby confirming their identity as EVs (Figure 1E). The absence of calnexin, a non‐EV marker, further validated the purity of the isolated MEVs (Figure 1E). To further assess whether HIV Tat influences the release of ferroptotic mediators in the BV2‐EVs, we next examined the expression of FTH1 and ACSL4 in the MEVs using western blotting. The results indicated that HIV Tat exposure significantly increased the expression levels of FTH1 by 4.32 fold (*p* = 0.0130, *n* = 6) and ACSL4 by 3.26 fold (*p* = 0.0323, *n* = 6) in Tat‐MEVs (Figure 1F,G) compared to control‐MEVs. Furthermore, pretreatment of BV2 cells with GW4869 downregulated Tat‐mediated expression of FTH1 by 4.63 fold (*p* = 0.0178, *n* = 6) and ACSL4 by 6.20 fold (*p* = 0.049, *n* = 6) compared to Tat‐MEVs (Figure S1B). Interestingly, while GW4869 (5 µM; 48 h) pretreatment downregulated ACSL4 expression in MEVs, it had differential effects on the intracellular expression of these markers in BV2 cells. Specifically, GW4869 pretreatment upregulated FTH1 expression by 1.30 fold (*p* = 0.0068, *n* = 3) while downregulating ACSL4 expression by 1.207 fold (*p* = 0.0014, *n* = 3) when compared to Tat‐MEVs (Figure S1C). Overall, these findings thus suggested that HIV Tat not only promoted the release of MEVs but also influenced the cargo content, leading to dissemination of ferroptotic signaling proteins into the extracellular environment.

### 3.2 HIV Tat‐Mediated Induction of Ferroptosis in Microglia Modulated the Release of Tat‐MEV Cargoes

**FIGURE 2:**
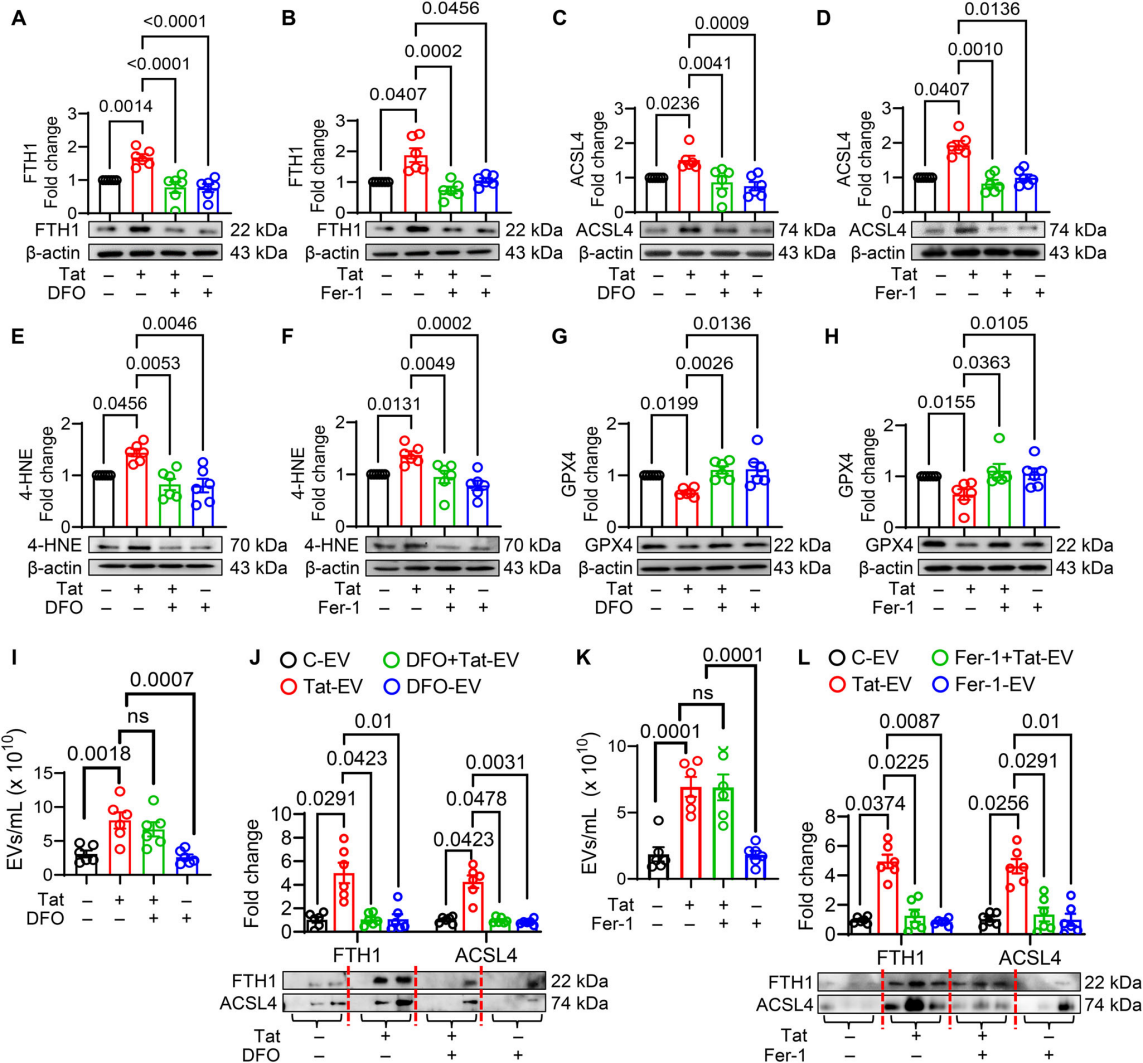
Inhibition of the ferroptosis pathway in BV2 cells decreased the release of ferroptotic cargo in BV2‐derived EVs. (A–H) Western blot and quantification showing expression of ferroptotic markers FTH1 (A, B), ACSL4 (C, D), 4‐HNE (E, F), and GPX4 (G, H) in HIV Tat‐exposed BV2 cells treated with ferroptosis inhibitors DFO or Fer‐1, respectively. β‐actin was used as a loading control. (I) Representative bar graph of EV release per mL from BV2 cells (control or exposed to HIV Tat, DFO+ HIV Tat, and DFO) and (J) western blot analysis and quantification of FTH1 and ACSL4 in BV2‐EVs. (K) Representative bar graph of EV release per mL and (L) western blot analysis and quantification of FTH1 and ACSL4 in BV2‐EVs (control or exposed to HIV Tat, Fer‐1+ HIV Tat, and Fer‐1) (K). Data is presented as mean ± SEM from six independent experiments. Abbreviations: EV: extracellular vesicles; DFO: deferoxamine; Fer‐1: ferrostatin; FTH1: ferritin heavy chain‐1; ACSL4: acyl‐CoA synthetase long chain family member 4; 4‐HNE: 4‐hydroxynonenal; Tat: trans‐activator of transcription;GPX4: glutathione reductase‐4.

We next sought to determine whether modulating the intracellular ferroptosis pathway could alter the ferroptotic cargo release in MEVs. Our findings indicated that both DFO and Fer‐1 significantly inhibited HIV Tat‐induced expression of ferroptosis mediators in BV2 cells compared with control cells. This was evidenced by a decrease in the expression of FTH1 by 2.1 fold in the presence of DFO (*p* < 0.0001, *n* = 6, Figure 2A) and 1.7 fold in the presence of Fer‐1 (*p* = 0.0002, *n* = 6; Figure 2B) in Tat‐exposed cells. Similarly, the expression of ACSL4 in the presence of DFO was decreased by 1.7 fold (*p* = 0.0041, *n* = 6; Figure 2C) and in the presence of Fer‐1 by 2.4 fold (*p* = 0.0010, *n* = 6; Figure 2D). Expression of 4‐HNE in the presence of DFO was decreased by 1.7 fold (*p* = 0.0053, *n* = 6, Figure 2E) and, in the presence of Fer‐1 by 1.4 fold (*p* = 0.0049, *n* = 6, Figure 2F). This was accompanied by a concomitant increase in the expression of the antioxidant enzyme GPX4, which in the presence of DFO was increased by 1.6 fold (*p* = 0.0026, *n* = 6, Figure 2G) and in the presence of Fer‐1 was increased by 1.7 fold (*p* = 0.0363, *n* = 6, Figure 2H) in BV2 cells. Pretreatment of BV2 cells with DFO did not significantly aller the total number of Tat‐MEVs released, with EV count remianing comparable to those observed in control MEVs (Figure 2I). However, DFO pretreatment significantly resulted in decreased expression of FTH1 by 4.7 fold (*p* = 0.0423, *n* = 6) and ACSL4 by 4.3 fold (*p* = 0.0478, *n* = 6) in Tat‐MEVs (Figure 2J). Similarly, pretreatment of cells with Fer‐1 did not significantly affect Tat‐MEV release, and EV counts remained comparable across treatment groups and control MEVs (Figure 2K). In contrast, Fer‐1 pretreatment significantly resulted in decreased expression of FTH1 by 3.9 fold (*p* = 0.0225, *n* = 6), and ACSL4 by 3.4 fold (*p* = 0.0291, *n* = 6) in Tat‐MEV cargoes (Figure 2L). These findings thus demonstrated that inhibition of intracellular ferroptosis signaling attenuated enrichment of the ferroptotic mediators in Tat‐MEV cargo without altering EV release.

### 3.3 HIV Tat‐Mediated Dysregulated Autophagy Regulates the Ferroptotic Cargo in MEVs

**FIGURE 3:**
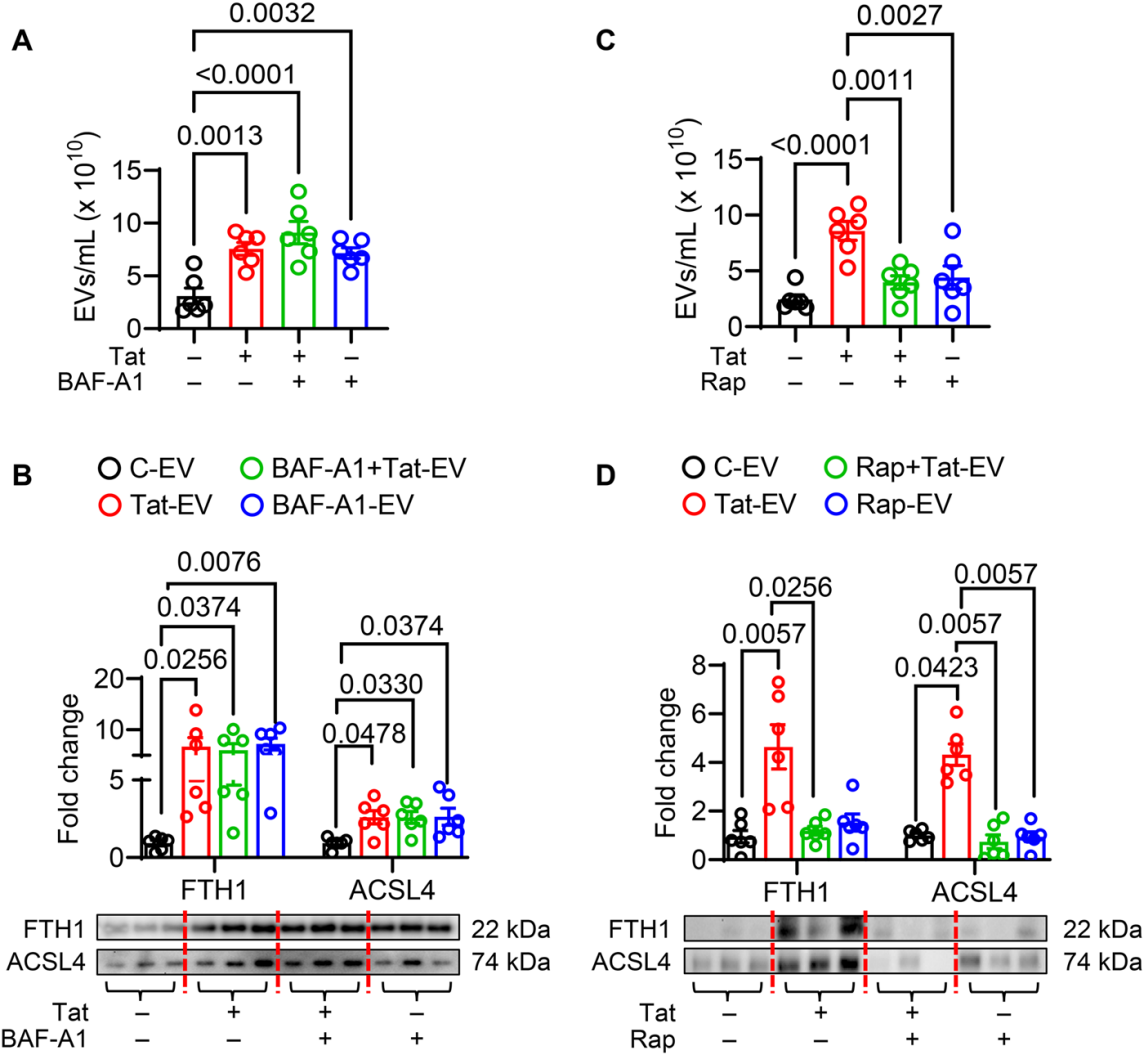
HIV Tat‐mediated dysregulated autophagy resulted in increased release of BV2‐derived EVs. (A) Representative bar graph showing total numbers of released EVs per mL in the supernatant collected from BV2 cells exposed to various conditions (control, HIV Tat, BAF‐A1+ HIV Tat, BAF‐A1) and, (B) western blot images and quantification of ferroptosis markers FTH1 and ACSL4 in these HIV Tat+BAF‐A1 exposed BV2‐EVs; (C) Representative bar graph showing the total numbers of released EVs per mL in the supernatant collected from BV2 cells exposed to various conditions (control, HIV Tat, Rap+ HIV Tat, Rap) and, (D) western blot images and quantification showing the expression of ferroptosis markers FTH1 and ACSL4 in these HIV Tat+Rap exposed BV2‐EVs. Data is presented as mean ± SEM from six independent experiments. Abbreviations: EV: extracellular vesicles; FTH1: ferritin heavy chain‐1; ACSL4: acyl‐CoA synthetase long chain family member 4; BAF‐A1: bafilomycin; Rap: rapamycin.

**FIGURE 4:**
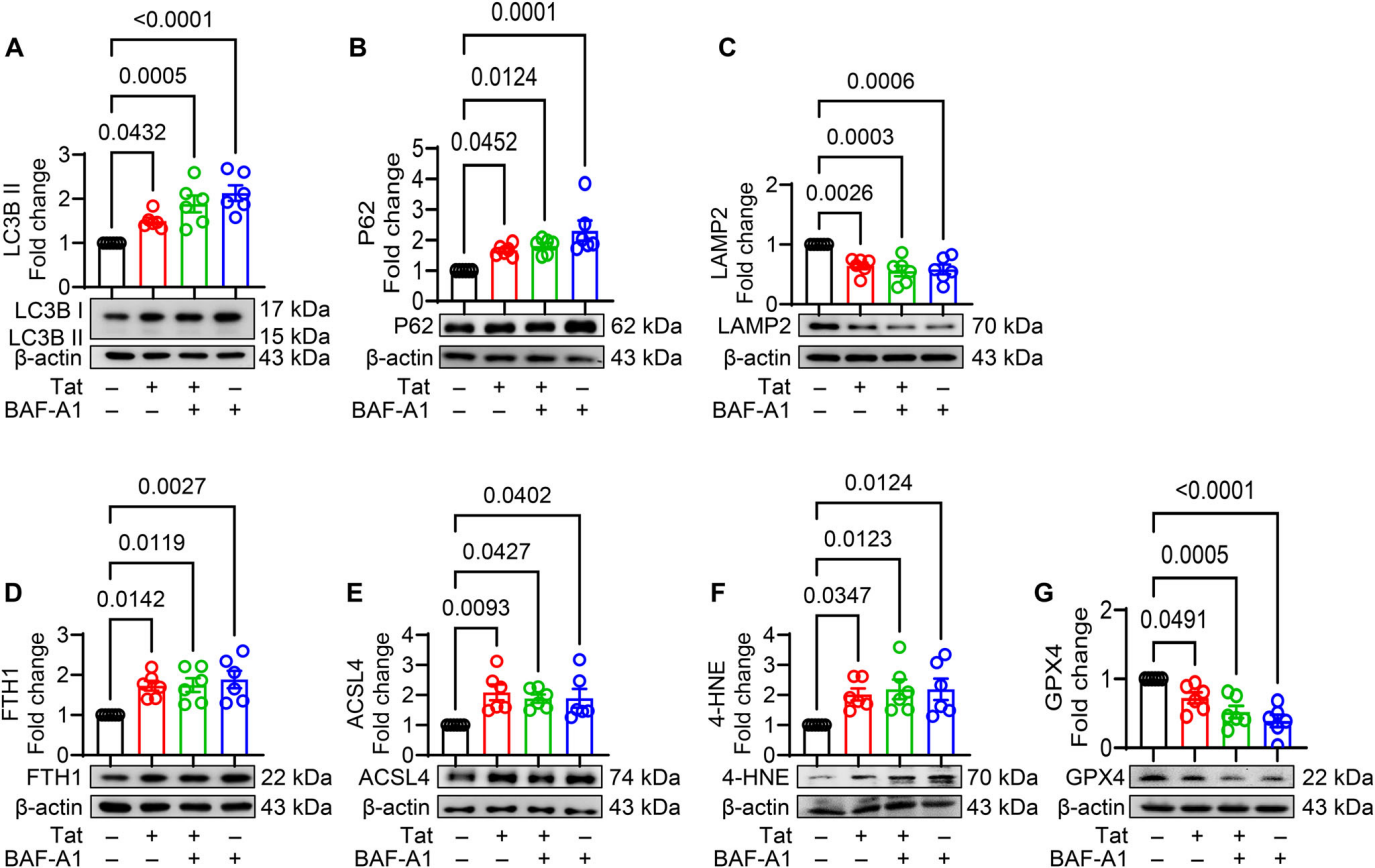
HIV Tat‐mediated dysregulated autophagy resulted in accumulation of ferroptosis mediators in BV2 cells. (A–G) Representative western blot images and quantification of autophagy markers—LC3B II (A), P62 (B), LAMP2 (C); and ferroptosis mediators—FTH1 (D), ACSL4 (E), 4‐HNE (F), and GPX4 (G) in HIV Tat (100 ng/mL) exposed BV2 cells cultured in the presence of the autophagy inhibitor BAF‐A1. β‐actin was used as a loading control. Data is presented as mean ± SEM of six independent experiments. Abbreviations: EV: extracellular vesicles; FTH1: ferritin heavy chain‐1; ACSL4: acyl‐CoA synthetase long chain family member 4; 4‐HNE: 4‐hydroxynonenal; Tat: trans‐activator of transcription; GPX4: glutathione reductase‐4; LAMP2: lysosomal associated membrane protein‐2; BAF‐A1: bafilomycin.

We next sought to investigate whether HIV Tat‐mediated dysregulated autophagy could influence EV release and the packaging of ferroptotic mediators in their cargo. Our results demonstrated that inhibition of microglial autophagy with BAF‐A1 increased the total number of Tat‐MEVs released (7.15 × 10^10^ ± 0.504 × 10^10^) compared to control cells (3.1 × 10^10^ ± 0.39 × 10^10^) (Figure 3A). Additionally, HIV Tat treatment in the presence of BAF‐A1 upregulated the expression of ferroptotic mediators in Tat‐MEVs as shown by increased expression of FTH1 by 7.2 fold (*p* = 0.0076, *n* = 6) and ACSL4 by 2.6 fold (*p* = 0.0374, *n* = 6) compared to control‐MEVs. Increased levels of both, FTH1 and ACSL4 by BAF‐A1 alone treatment remained comparable to Tat‐MEVs (Figure 3B). Conversely, rapamycin pretreatment reduced the total number of Tat‐MEVs (3.96 × 10^10^ ± 0.6 × 10^10^) compared with Tat‐MEV numbers without rapamycin pretreatment (8.58 × 10^10^ ± 0.84 × 10^10^) (Figure 3C). Rapamycin pretreatment also downregulated the expression of FTH1 by 3.9 fold (*p* = 0.0256, *n* = 6) and that of ACSL4 by 5.9 fold (*p* = 0.0057, *n* = 6) compared to Tat‐MEV cargo (Figure 3D). Furthermore, BV2 cells exposed to HIV Tat in the presence of BAF‐A1 treatment showed significantly increased expression of LC3B II by 1.88 fold (*p* = 0.0005, *n* = 6; Figure 4A) as well as that of P62 by 1.8 fold (*p* = 0.0124, *n* = 6; Figure 4B), while downregulating the expression of LAMP2 by 1.9 fold (*p* = 0.0003, *n* = 6; Figure 4C) compared with control cells. These levels were comparable to those observed in the HIV Tat‐alone group. This effect of Tat together with BAF1 was further accompanied by upregulated expression of the ferroptosis mediators—FTH1 by 1.7 fold (*p* = 0.0119, *n* = 6; Figure 4D), ACSL4 by 1.9 fold (*p* = 0.0427, *n* = 6; Figure 4E), and 4‐HNE by 2.2 fold (*p* = 0.0123, *n* = 6; Figure 4F), compared to control cells and, was also accompanied with a concomitant downregulation of GPX4 by 1.9 fold (*p* = 0.0005, *n* = 6; Figure 4G). In contrast, pretreatment with rapamycin (autophagy inducer) resulted in a 1.7 fold (<0.0001) increase in expression of LC3B II compared to control cells (no significant change observed compared to the HIV Tat alone group) (Figure S2A). LAMP2 expression was increased by 2.2 fold (*p* = 0.005, *n* = 6; Figure S2C), while that of P62 was decreased by 3.2 fold (*p* < 0.001, *n* = 6; Figure S2B) compared to HIV Tat alone exposed cells, thereby implicating enhanced autophagosome clearance. This was accompanied by reduced expression of ferroptosis‐associated mediators‐ FTH1 by 2.5 fold, (*p* < 0.0001, *n* = 6); ACSL4 by 1.6 fold (*p* = 0.0003, *n* = 6) and 4‐HNE by 1.5 fold (*p* = 0.0199, *n* = 6) with subsequent increase in GPX4 expression by 1.9 fold (*p* < 0.0001, *n* = 6) compared to HIV Tat alone exposed BV2 cells (Figure S2D–G).

**FIGURE 5:**
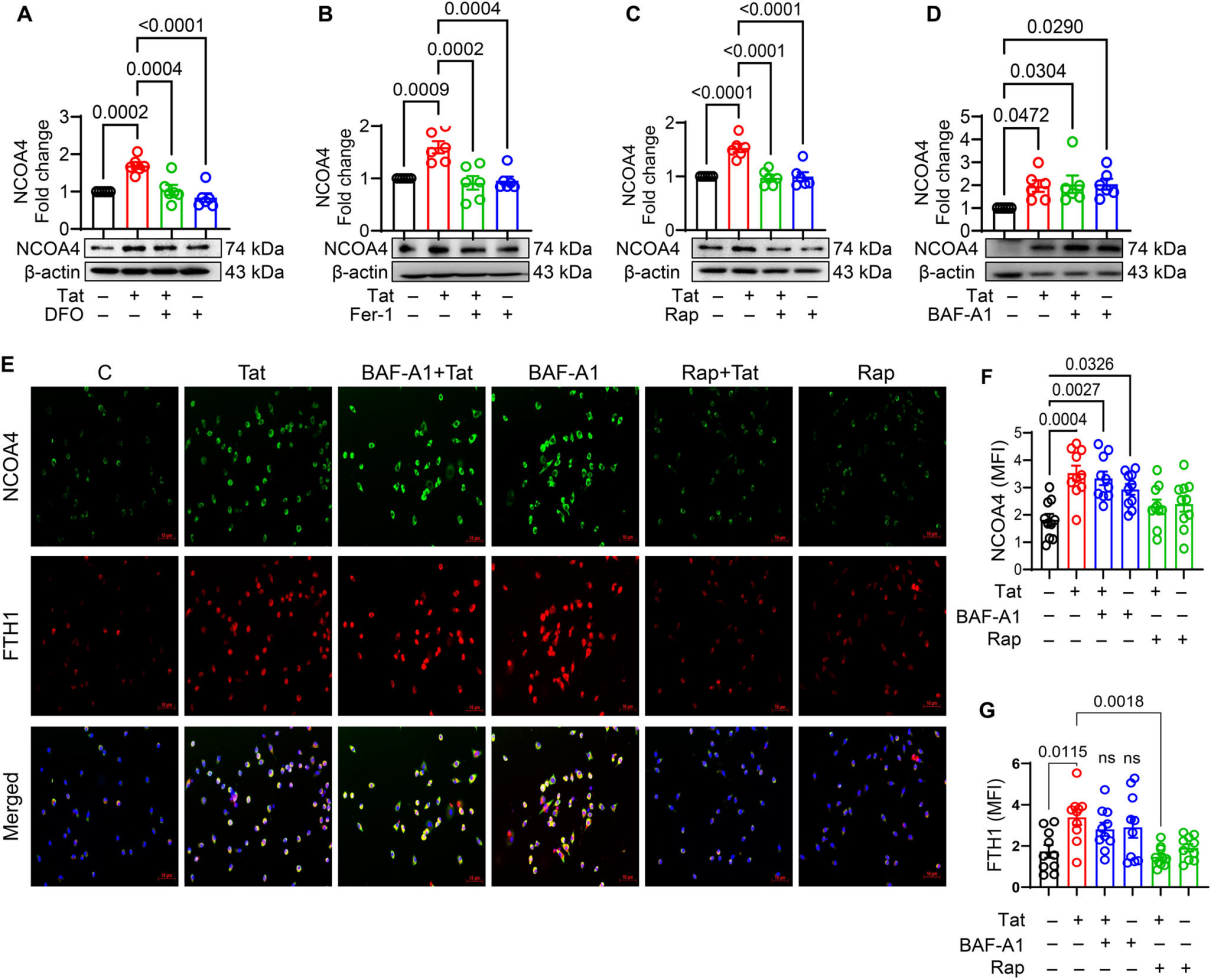
HIV Tat exposure impaired ferritinophagy in BV2 cells. (A–D). Representative western blot images and quantification of NCOA4 expression in the presence of DFO (A), Fer‐1 (B), Rap (C), and BAF‐A1 (D). β‐actin was used as a loading control. (E–G) Representative immunofluorescent staining for co‐localized NCOA4 and FTH1 and corresponding graph in BV2 cells exposed to HIV Tat in the presence or absence of bafilomycin or rapamycin. Scale bar: 10 µm. Data is presented as mean ± SEM of six independent experiments. Abbreviations: NCOA4: nuclear receptor coactivator‐4; DFO: deferoxamine; Fer‐1: ferrostatin; BAF‐A1: bafilomycin; Rap: rapamycin; Tat: trans‐activator of transcription; FTH1: ferritin heavy chain‐1, MFI: mean fluorescent intensity.

FTH1 undergoes autophagic degradation through a process known as ferritinophagy. We thus next sought to determine the expression of NCOA4 in HIV Tat‐exposed BV2 cells. There was significantly increased expression of NCOA4 by 1.5 fold (*p* = 0.0432, *n* = 6; Figure 5A) in the presence of HIV Tat in BV2 cells. HIV Tat‐mediated upregulation of NCOA4, however, was mitigated in BV2 cells pretreated with the ferroptosis inhibitors DFO by 1.67 fold (*p* = 0.0004, *n* = 6; Figure 5A) or Fer‐1 by 2 fold (*p* = 0.0002, *n* = 6; Figure 5B) verses HIV Tat‐treated cells. NCOA4 expression was also reduced in cells pretreated with autophagy inducer rapamycin by 1.67 fold (*p* < 0.0001, *n* = 6; Figure 5C). Treatment of BV2 cells with BAF‐A1, on the other hand, did not result in a further increase in the expression of NCOA4 (Figure 5D). This was further validated by immunofluorescence imaging, demonstrating increased mean fluorescence intensity by 1.9 fold (*p* = 0027, *n* = 6) and colocalization of NCOA4 with FTH1 in BV2 cells treated with BAF‐A1 (Figure 5E–G) in the presence of HIV Tat. HIV Tat‐mediated upregulation of NCOA4, however, was ameliorated in cells pretreated with either rapamycin (Figure 5E–G), DFO or Fer‐1 (Figure S3). Collectively, these results demonstrated that HIV Tat‐induced impairment of autophagic flux impaired ferritinophagy, in turn, leading to enhanced microglial ferroptosis, as well as release of ferroptotic mediators via Tat‐MEVs.

### 3.4 HIV Transgenic Rats Demonstrated Dysregulated Autophagy and Ferroptosis in Microglia

**FIGURE 6:**
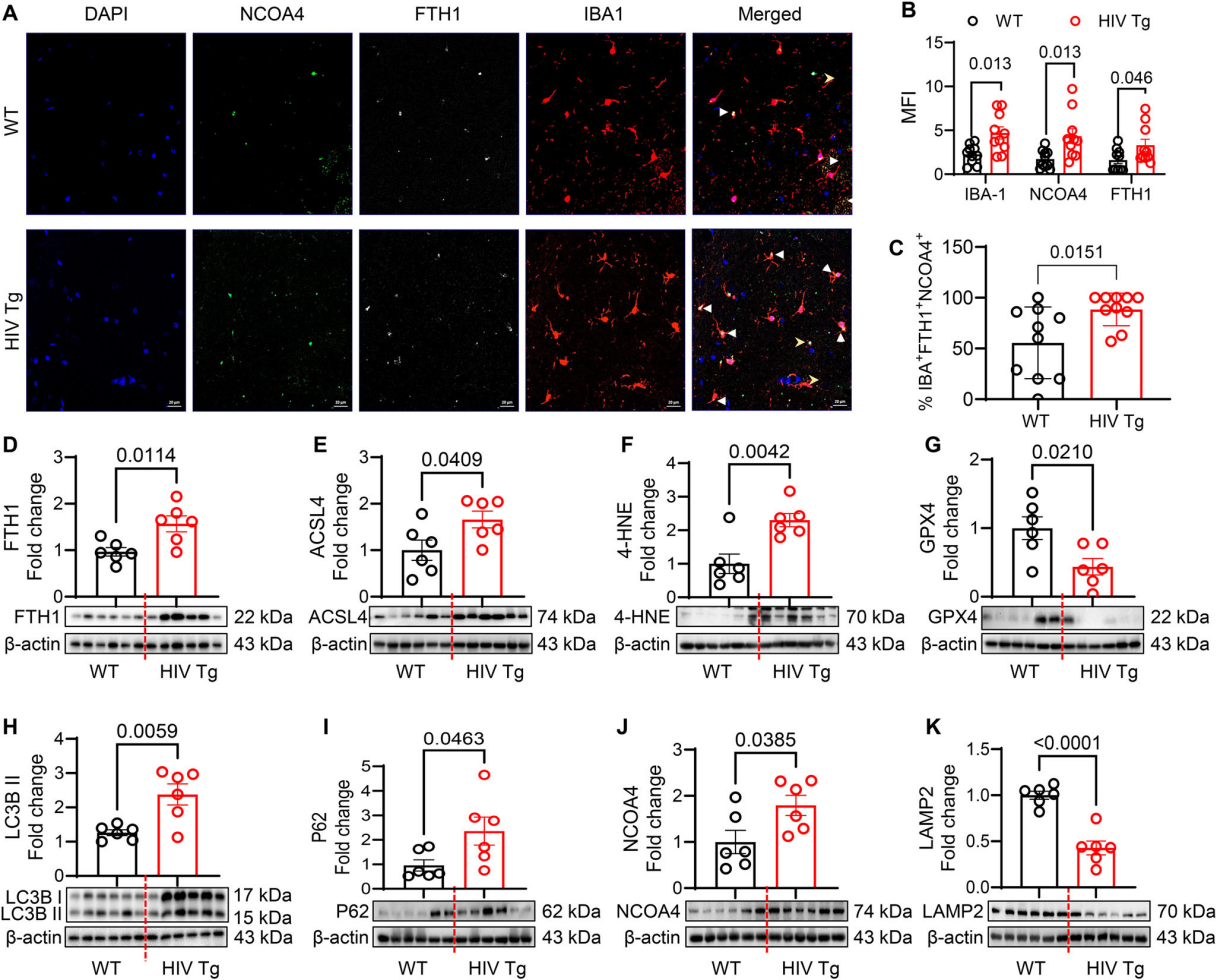
In vivo validation of upregulated ferroptosis mediators in the brains of HIV Transgenic rats. (A–C) Representative immunofluorescent staining and corresponding graphs for IBA‐1, FTH1, and NCOA4 in 16–18‐month‐old male rat cortical brain sections showing colocalization of FTH1 with IBA‐1 signals. White arrows represent the colocalization of FTH1 and NCOA4 with IBA1, and yellow arrows represent the colocalization of FTH1 and NCOA4 in other cells. Scale bar: 20 µm. (D–K) Western blot images and corresponding quantification graphs showing expression of ferroptosis markers—FTH1 (D), ACSL4 (E), 4‐HNE (F), and GPX4 (G) and autophagic markers—LC3B II (H), P62 (I), NCOA4 (J), and LAMP‐2 (K), in the brain lysates (frontal cortex) of HIV Tg and wildtype control rats. β‐actin was used as a loading control. Data is presented as mean ± SEM of six independent experiments. Abbreviations: ACSL4: acyl‐CoA synthetase long chain family member 4; FTH1: ferritin heavy chain; 4‐HNE: 4‐hydroxynonenal; LC3B II: microtubule associated protein light chain 3B; NCOA‐4: nuclear receptor coactivator‐4; LAMP2: lysosomal associated membrane protein‐2; GPX‐4: glutathione peroxidase‐4, MFI: mean fluorescent intensity.

Next, we sought to validate our in vitro findings in brains of HIV Tg and wildtype rats. There was significantly upregulated expression of FTH1 by 2 fold (*p* = 0.046), NCOA4 by 2.5 fold (*p* = 0.013), and microglial activation marker, IBA1 by 2 fold (*p* = 0.013) in the cortices of HIV Tg rats compared to those of control rats (*n* = 6) (Figure 6A,B). Increased colocalization of FTH1 with NCOA4 was also evident in IBA1‐positive cells by 1.6 fold (*p* = 0.0151, Figure 6C), thus implying that activated microglia exhibited increased expression of FTH1, and that this was associated with increased expression of NCOA4. Western blotting analysis further confirmed these observations, showing upregulation of ferroptosis markers—FTH1 by 1.6 fold (*p* = 0.0114, Figure 6D), ACSL4 by 1.7 fold (*p* = 0.0409, Figure 6E), and 4‐HNE by 2.3 fold, *p* = 0.0042, Figure 6F), with a concomitant downregulation of GPX4 by 2.2 fold, (*p* = 0.0210, Figure 6G) in the frontal cortices of HIV Tg rats compared to wildtype controls. We also determined the expression of autophagy markers in the brains of rats and found increased expression of LC3B II by 1.8 fold, (*p* = 0.0059, Figure 6H), P62 by 2.4 fold, (*p* = 0.0463, Figure 6I), and NCOA4 by 1.8 fold (*p* = 0.0385, Figure 6J), with decreased expression of LAMP2 by 2.3 fold (*p* < 0.0001, Figure 6K) in the frontal cortices of HIV Tg rats compared with the wildtype rats.

### 3.5 Secretion of Ferroptotic Cargoes in BEVs From HIV Tg Rats

**FIGURE 7:**
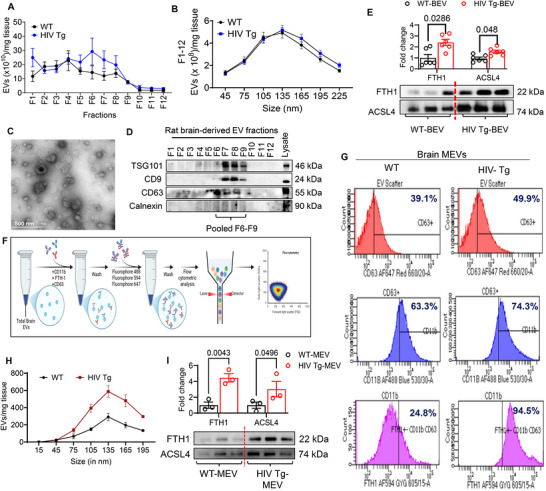
Rat brain‐derived EVs (BEVs) contained ferroptosis cargoes. (A) Total number of brain‐derived EVs per mg tissues and (B) size distribution determined by NTA using ZetaView; (C) Representative image of BEVs captured by TEM. Scale bar 500 nm; (D) Western blot images showing the expression of EV markers—TSG101, CD9, and CD63. Calnexin was used as a negative control for EV purity. (E) Western blot images and quantification of FTH1 and 4‐HNE in the BEVs isolated from HIV Tg or wildtype rats. (F) A schematic illustration showing the protocol for sample preparation of rat brain‐derived MEVs by flow cytometry; (G) Fluorescent histogram analysis showing percentage of CD63^+^ MEVs in the brains of HIV Tg rats compared to wild type brain MEVs as well as subpopulations CD63^+^CD11b^+^ and CD63^+^CD11b^+^FTH‐1^+^ EVs; (H) Particle size distribution was determined by NTA using ZetaView; (I) Western blot analysis and quantification showing ferroptosis markers FTH1 and ACSL4 in the brain MEVs from HIV Tg rats compared to those from wild type control brain MEVs. Data is presented as mean ± SEM of three independent experiments. Abbreviations: EV: extracellular vesicles; WT‐BEV: wild‐type brain extracellular vesicles; Tg rat‐BEV: Transgenic rat brain extracellular vesicle; TEM; transmission electron microscopy; FTH1: ferritin heavy chain‐1; ACSL4: acyl‐CoA synthetase long chain family member 4.

To further validate that HIV Tat can also cause upregulated expression of the ferroptotic cargoes in vivo, BEVs (total EVs derived from all brain cells including microglia, astrocytes, neurons, and endothelial cells) were isolated from both wildtype and HIV Tg rats (*n* = 3). BEVs were isolated using the protocol depicted in (Figure S4), as previously reported by us (Dagur et al. 2020; Sil et al. 2021). NTA analysis revealed an increase in the number of BEVs in fractions 6–7, with a size range of 45–195 nm (Figure 7A,B), in HIV Tg rats compared to wildtype controls, peaking at 135 nm. TEM analysis confirmed these particles had a structure representative of EVs (Figure 7C and Figure S5). Western blotting analysis of the EV‐specific markers TSG101, CD9, and CD63 further confirmed that fractions 6–9 contained EVs (Figure 7D). Pooled fractions 6–9 were then analyzed for the presence of ferroptotic cargoes FTH1 and ACSL4. The results showed significantly upregulated expression of both FTH1 by 2.4 fold (*p* = 0.0286, *n* = 3) and ACSL4 by 1.6 fold (*p* = 0.048, *n* = 3) in BEVs isolated from HIV Tg rats compared to wildtype controls (Figure 7E).

### 3.6 Secretion of Ferroptotic Mediators in MEV Cargoes Isolated From Brains of HIV Tg Rats

Next, to further determine the contribution of MEVs (microglia specific CD63+CD11B^+^ EVs) in the total BEVs, pooled fractions 6–9 (EV‐enriched fractions) were subjected to flow cytometry (using the protocol described in Figure 7F) using anti‐CD63 as an EV‐specific marker, anti‐CD11b as the microglial‐specific marker, and anti‐FTH1. As shown in Figures S6–S8, single EV events were first gated on CD63 positivity within gates defined by matched isotype and secondary‐only controls to quantify total EVs. Within the CD63^+^ population, MEVs were identified by CD11b positivity. Subsequently, FTH1 expression was analyzed within the CD63^+^CD11b^+^ EV population to assess cargo enrichment in MEVs. Our results demonstrated enrichment of CD63^+^ EVs in samples isolated from HIV Tg rats (49.9%) compared with the wildtype controls (39.1%) (Figures 7G and S9). CD63^+^ EVs were further enriched for microglia‐specific markers, as evidenced by an increased CD11b^+^CD63^+^ EV population in BEVs from HIV Tg rats, suggesting that HIV Tg rats release higher levels of MEVs (approximately 10% more) compared with wildtype controls. Furthermore, FTH1 protein expression was highest in the CD63^+^/CD11b^+^ EV population isolated from HIV Tg rat brains compared to the wildtype controls. To confirm that the detected events represented single EV particles rather than aggregates or coincident events, BEVs from both WT and Tg rats were serially diluted and analyzed. As shown in Supplementary material 1 and 2, event counts decreased proportionally with dilution, while scatter properties (FSC/SSC) and cargo distribution remained unchanged. These findings support the detection of single vesicles and exclude swarm effects. Furthermore, following flow cytometry acquisition, BEV samples were treated with Triton X‐100 (final concentration 10%). This resulted in an approximately 86% reduction in detectable events (Figure S10), confirming the majority of signals in fractions F6–F9 originated from membrane‐bound EVs. In addition, NTA analysis showed increased numbers of MEVs from the brains of HIV Tg rats compared to those from wild type controls (Figure 7H). Western blot results further validated increased expression of FTH1 by 4.4 fold, (*p* = 0.0043, *n* = 3) and ACSL4 by 3 fold, (*p* = 0.0496, *n* = 3) in MEVs isolated from the brains of HIV Tg rats compared to WT‐MEVs (Figure 7I), normalized with MEV numbers. Taken together, these findings suggest that MEVs are a major contributor to the elevated FTH1 levels observed in BEVs from HIV‐Tg rats.

## 4 Discussion

In the cART era, the landscape of NeuroHIV in PLWH has shifted significantly, manifesting as increased prevalence of asymptomatic neurocognitive impairments and mild neurocognitive disorders (Nightingale et al. 2014; Saylor et al. 2016). The underlying mechanisms of NeuroHIV are complex and multifactorial, with HIV infection of microglia playing a critical role. Microglia serve as viral reservoirs in the brain, where HIV continues to replicate persistently, albeit at low levels, despite treatment with cART. A key viral protein produced persistently by infected microglia in PLWH (under cART exposure) is HIV Tat, which plays a multifaceted role in the pathogenesis of NeuroHIV (Henderson et al. 2019; Johnson et al. 2013).

Previous findings from our group suggest that HIV Tat exposure initiates an early intracellular stress response in microglia characterized by oxidative stress, increased labile iron accumulation, mitochondrial dysfunction and activation of inflammasome and ferroptosis‐associated pathways, (Chivero et al. 2017; Kannan et al. 2022; Thangaraj et al. 2018). HIV Tat has also been shown to upregulate miR‐204 in microglia while suppressing the cystine/glutamate antiporter system Xc^−^ component SLC7A11, along with several antioxidant defense enzymes Nrf2 and GPX4 (Gupta et al. 2010; Kannan et al. 2022; Lin et al. 2023; Yang et al. 2022). These alterations disrupt intracellular redox homeostasis by increasing the labile iron pool and weakening antioxidant capacity, thereby promoting lipid peroxidation and ultimately contributing to ferroptotic cell death (Kannan et al. 2022). Building upon these observations, in the current study, we sought to examine whether HIV Tat exposure could also alter the release of ferroptosis‐associated mediators via MEVs, with a specific focus on the contributions of impaired ferritinophagy and dysfunctional lysosomes. Our studies suggested that HIV Tat‐induced ferroptotic signaling was not limited to intracellular stress responses but was also accompanied by selective extracellular export of ferroptosis‐associated cargoes in Tat‐MEVs, resulting in propagation of pathogenic signaling within the CNS.

EVs have been increasingly recognized as important mediators in various neurodegenerative diseases, including NeuroHIV (Chemparathy et al. 2024; Hill 2019; Hu et al. 2016, 2018, 2020; Quek and Hill 2017). EVs released from microglia, astrocytes, and neurons, carry and transport proteins, lipids, inflammatory mediators, and oxidative stress–related molecules modulating the function of recipient cells (Chemparathy et al. 2024; Hu et al. 2016; Lombino et al. 2025; Marangon et al. 2022; Schnatz et al. 2021). In the context of NeuroHIV, EVs from HIV‐infected cells and activated glial cells have been implicated to contribute to neuronal damage through multiple mechanism(s) (Caobi et al. 2023; Chemparathy et al. 2024; Kannan et al. 2022). They have been shown to transfer miRNAs, amyloid β, inflammasome and pro‐inflammatory cytokines, to cells, such as astrocytes and neurons, ultimately culminating in microglial activation/neuronal injury and/or cell death (Hu et al. 2020; Kannan et al. 2022; Sil et al. 2021; Yang et al. 2018). In this context, our current findings have expanded the emerging role of EVs by demonstrating that ferroptosis‐associated mediators, including FTH1 and ACSL4, were also enriched within Tat‐MEVs.

Importantly, the presence of ferroptotic cargo within Tat‐MEVs appears to represent an active stress‐adaptive response rather than a passive byproduct of dying cells. Inhibition of ferroptosis with deferoxamine or ferrostatin‐1 reduced ferroptotic cargoes in MEVs without significantly altering total MEV release. Our findings also indicated that ferroptosis‐associated cargo loading and EV secretion are two distinct processes. Whereas ferroptosis inhibition reduced intracellular ferroptotic burden, EV biogenesis and release itself remained unaffected, suggesting thereby that cargo selection rather than vesicle release was preferentially regulated under ferroptotic stress. In line with these observations, other studies have reported that EVs can transport oxidized lipids, ferritin, transferrin receptor 1, and divalent metal transporter 1, thereby facilitating iron dysregulation and lipid peroxidation in recipient cells (Chen and Tang 2023; Gunshin et al. 2001; Huang et al. 2026; Ito et al. 2021; Mattera et al. 2020). The EV cargoes could promote ferroptotic signaling in neighboring glia or neurons, thus amplifying the effects of localized microglial stress. Tat‐MEVs could therefore serve as vehicles for disseminating ferroptotic and inflammatory signals throughout the CNS, potentially contributing to neuronal dysfunction and cognitive decline associated with NeuroHIV. Taken together it can be thus speculated that during early‐to‐intermediate stages of HIV Tat‐mediated activation, microglia could utilize EV secretion as a mechanism to export toxic intracellular cargo and restore cellular homeostasis.

Our in vitro observations were further supported by *ex vivo* findings demonstrating the presence of FTH1 in brain‐derived MEVs isolated from HIV Tg rats. While HIV Tg rats expressing multiple viral proteins are a good model for validating the in vitro effects of Tat, since these rats also express other viral proteins, a possible limitation is that the observed effects cannot solely be attributed to the effect of Tat alone. Additionally, since these rats carry HIV proteins from birth, this can also contribute to increased systemic and neuroinflammation with age (due to accumulation of viral proteins) and hence can be an indirect contributing factor in the release of MEVs. Additionally, although brain‐derived MEVs were enriched using the microglia‐specific marker CD11b, contributions from other glial or brain cell‐derived EVs cannot be completely excluded and also remains a limitation of the current study.

Autophagy plays a multifaceted role in regulating EV biogenesis and secretion, with both pathways sharing intracellular trafficking machinery involved in cargo sorting and vesicle maturation (Eitan et al. 2016; Leidal et al. 2020; Murrow et al. 2015; Schuck 2020; van der Grein et al. 2019). Pharmacological modulation of autophagy significantly altered MEV release as well as cargo composition (Alves et al. 2020; Wang et al. 2025), implicating the role of dysregulated autophagic flux in the release of ferroptotic cargoes in MEVs. Inhibition of autophagic flux using bafilomycin A1 potentiated release of both Tat‐MEVs and their associated cargoes, whereas activation of autophagy with rapamycin ameliorated these effects. These observations are consistent with previous studies demonstrating that impaired lysosomal degradation shifts intracellular cargo toward EV‐mediated secretion (Alvarez‐Erviti et al. 2011; Cazzaro et al. 2022; Eitan et al. 2016).

Autophagy and EV release likely reflect selective mechanisms underlying the degradative and secretory pathways, respectively (Hartmann et al. 2024; Li et al. 2023; Solvik et al. 2022). Under normal physiological conditions, damaged proteins and organelles are directed toward lysosomal degradation through autophagy. However, when lysosomal clearance is impaired, cells compensate by redirecting the un‐degraded cargo into EVs for extracellular disposal. In the context of HIV Tat exposure, impaired autophagic flux could likely facilitate the accumulation and extracellular export of ferroptosis‐associated cargoes. HIV Tat has previously been shown to accumulate within endolysosomal compartments and impair lysosomal acidification (Hui et al. 2012; Khan et al. 2022), resulting in defective autophagic degradation. Since lysosomal function is essential for maintaining autophagic flux, Tat‐mediated lysosomal dysfunction is suggestive as a contributor of autophagosome accumulation and defective cargo clearance. This disruption provides a mechanistic link between HIV Tat exposure, impaired autophagy, and enhanced EV‐mediated export of the ferroptotic cargo.

A particularly important aspect of impaired autophagy in this context involves ferritinophagy, the selective autophagic degradation of ferritin mediated by the NCOA4 adaptor protein. HIV Tat increased intracellular accumulation of both ferritin and NCOA4, suggesting disruption of ferritinophagic flux. Enhanced ferritin accumulation of these proteins likely suggests impaired lysosomal processing of the ferritin‐NCOA4 complexes. Consistent with this, Bafilomycin‐A1 treatment enhanced NCOA4 accumulation, supporting the notion that blockade of autophagosome–lysosome fusion prevented degradation of ferritinophagy intermediates. Under these conditions (both in HIV Tat and Bafilomycin‐A1 treated microglia), accumulated ferritin complexes could be rerouted toward EV secretion pathways which would need to be cleared by the cells. This phenomenon has been reported in various other contexts (Grisard et al. 2022; Minakaki et al. 2018; Tanaka et al. 2024)

Thus, impaired ferritinophagy not only contributes to intracellular iron dysregulation but also increases the extracellular dissemination of ferritin‐associated cargo via MEVs. These findings were also validated in frontal cortices of HIV Tg rats, wherein increased expression of the autophagy‐associated markers in the lysates as well as colocalization of FTH1 with NCOA4 in tissue sections suggested impaired ferritinophagy in vivo. We have previously demonstrated elevated expression of ferroptosis‐related mediators, oxidative stress markers, and microglial activation in the frontal cortices of HIV Tg rats and HIV‐positive human brain tissues. Notably, *ex vivo* microglia isolated from HIV Tg rats also exhibited increased expression of FTH1, ACSL4, and 4‐HNE concomitant with reduced expression of GPX4 levels.

**FIGURE 8:**
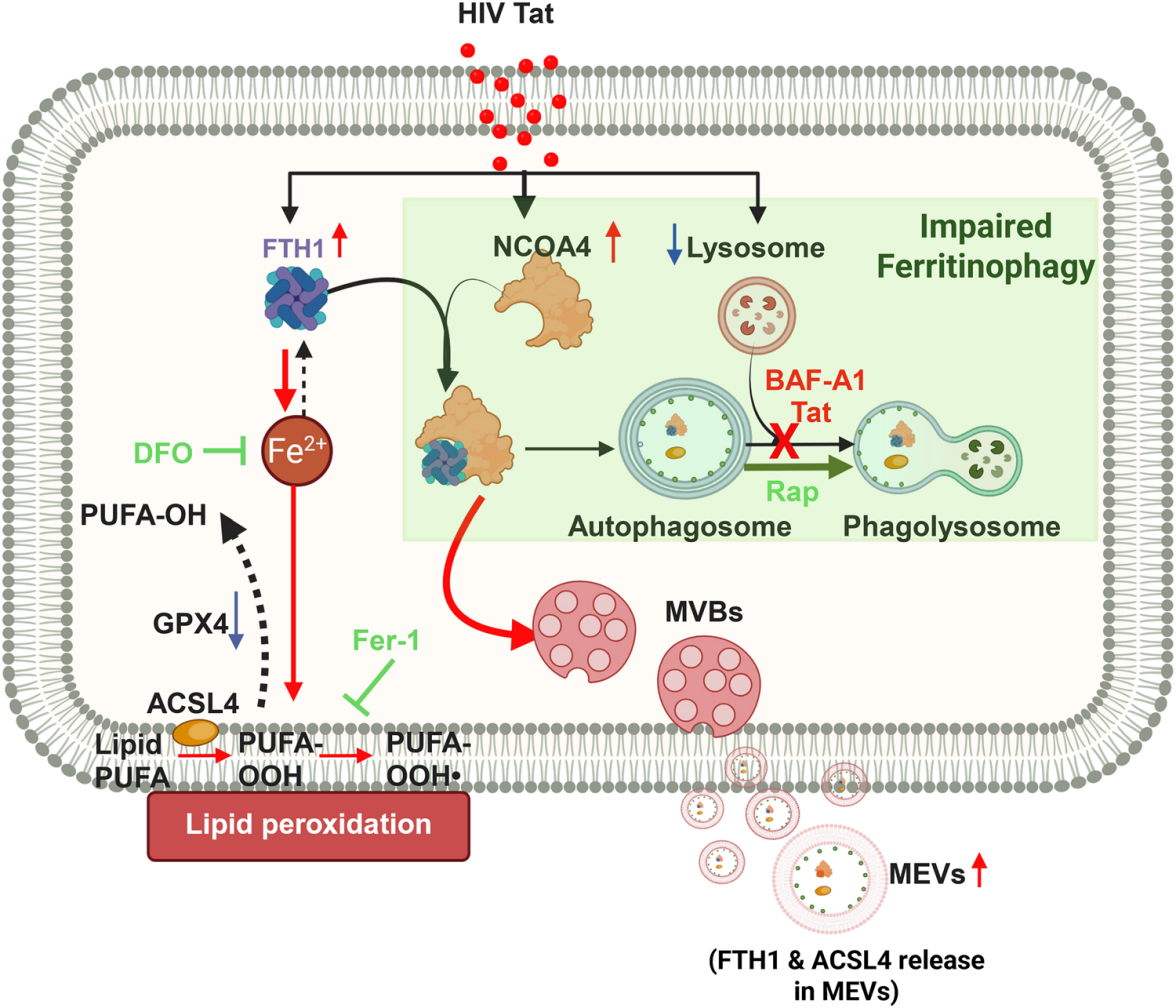
A schematic representation showing the role of dysregulated ferritinophagy in EV cargo release in HIV Tat‐exposed microglia. Exposure of microglia to HIV Tat protein leads to dysregulated expression of ferroptosis mediators, increased FTH1, ACSL4, 4‐HNE, and downregulated GPX4. This was also accompanied by increased expression of the nuclear receptor coactivator 4 (NCOA4), a cargo receptor that mediates the autophagic degradation of ferritin. Additionally, exposure of microglia to HIV Tat has also been shown to result in impaired lysosomal functioning (LAMP2 downregulation), thereby preventing the degradation of NAOA4/FTH1 complex by autophagosome‐lysosomal fusion, resulting in dysregulated ferritinophagy. This, in turn, leads to the release by the cells of excess, damaged, or misfolded proteins such as FTH1 into the extracellular space via EVs, likely as a compensatory mechanism. Image created with BioRender.com. Abbreviations: FTH1: ferritin heavy chain‐1; NCOA‐4: nuclear receptor coactivator‐4; LAMP2: lysosomal associated membrane protein‐2; GPX‐4: glutathione peroxidase‐4, ACSL4: acyl‐CoA synthetase long chain family member 4; PUFA: polyunsaturated fatty acid; DFO: deferoxamine; Fer‐1: ferrostatin‐1; BAF‐A1: bafilomycin A1; Rap: rapamycin, MEB: multivesicular bodies; MEV: microglial extracellular vesicles.

Collectively, as shown in Figure 8, our findings identify a mechanistic link between HIV Tat‐induced autophagic dysfunction, impaired ferritinophagy, and extracellular dissemination of ferroptosis‐associated cargo through MEVs. It can thus be envisioned that ferroptosis‐related EV cargo could serve as an important mediator of intercellular communication within the CNS and contribute to progressive neurodegenerative changes in the context of NeuroHIV.

There are several limitations to this study as well. Although our data demonstrates a strong association between ferroptosis markers, impaired ferritinophagy, and MEV cargo release, it does not establish whether ferroptosis is a primary initiating event or a secondary consequence of chronic inflammation and oxidative stress. Furthermore, while MEVs likely contribute to propagation of injury signals, the direct effects of Tat‐MEV cargo on recipient neurons or astrocytes remains to be fully elucidated. Future studies investigating cargo‐specific transfer mechanisms and downstream signaling pathways in recipient cells are warranted. Although both male and female rats were included in this study to enhance translational relevance, since HIV affects individuals of both sexes, the study was not well powered to assess sex as a biological variable and the analyses was therefore not stratified by sex. This remains a limitation of the current study, and future studies specifically designed to evaluate sex‐dependent differences will be important to elucidate potential sex‐specific mechanisms.

## Author Contributions

**Seema Singh**: conceptualization, writing – review and editing, writing – original draft, investigation, validation, visualization, formal analysis, methodology, data curation. **Elias Horanieh**: writing – review and editing, methodology, validation. **Craig L. Semerad**: methodology, data curation, formal analysis. **Palsamy Periyasamy**: conceptualization, writing – review and editing, resources, supervision. **Susmita Sil**: conceptualization, methodology, writing – review and editing, supervision. **Shilpa Buch**: conceptualization, writing – review and editing, resources, supervision.

## Funding

This project was supported by start‐up funds (S.B.) from the University of Nebraska Medical Center, Nebraska, USA. The support from the Nebraska Center for Substance Abuse Research (NCSAR) is also highly acknowledged.

## Ethics Statement

The animal experiment is approved by the ethical committee of the University of Nebraska Medical Center.

## Conflicts of Interest

The authors declare no conflicts of interest.

## Supporting information




**Supporting Information**: jex270153‐sup‐0001‐FigureS1.tif

**Supporting Information**: jex270153‐sup‐0002‐FigureS2.tif

**Supporting Information**: jex270153‐sup‐0003‐FigureS3.tif

**Supporting Information**: jex270153‐sup‐0004‐FigureS4.tif

**Supporting Information**: jex270153‐sup‐0005‐FigureS5.tif

**Supporting Information**: jex270153‐sup‐0006‐FigureS6.tif

**Supporting Information**: jex270153‐sup‐0007‐FigureS7.tif

**Supporting Information**: jex270153‐sup‐0008‐FigureS8.tif

**Supporting Information**: jex270153‐sup‐0009‐FigureS9.tif

**Supporting Information**: jex270153‐sup‐0010‐FigureS10.tif

**Supporting Information**: jex270153‐sup‐0011‐SuppMat.xls

**Supporting Information**: jex270153‐sup‐0012‐SuppMat.pdf

## Data Availability

The data that support the findings of this study are available from the corresponding authors upon request.
