# Supplementary figures and images for: HIV Tat‐Stimulated Microglial Extracellular Vesicles Are Enriched for Ferroptosis Mediators: Role of Dysregulated Autophagy
Source: J Extracell Biol. 2026 Jun 11;5(6):e70153. doi: 10.1002/jex2.70153 (PMC13257887; doi:10.1002/jex2.70153)

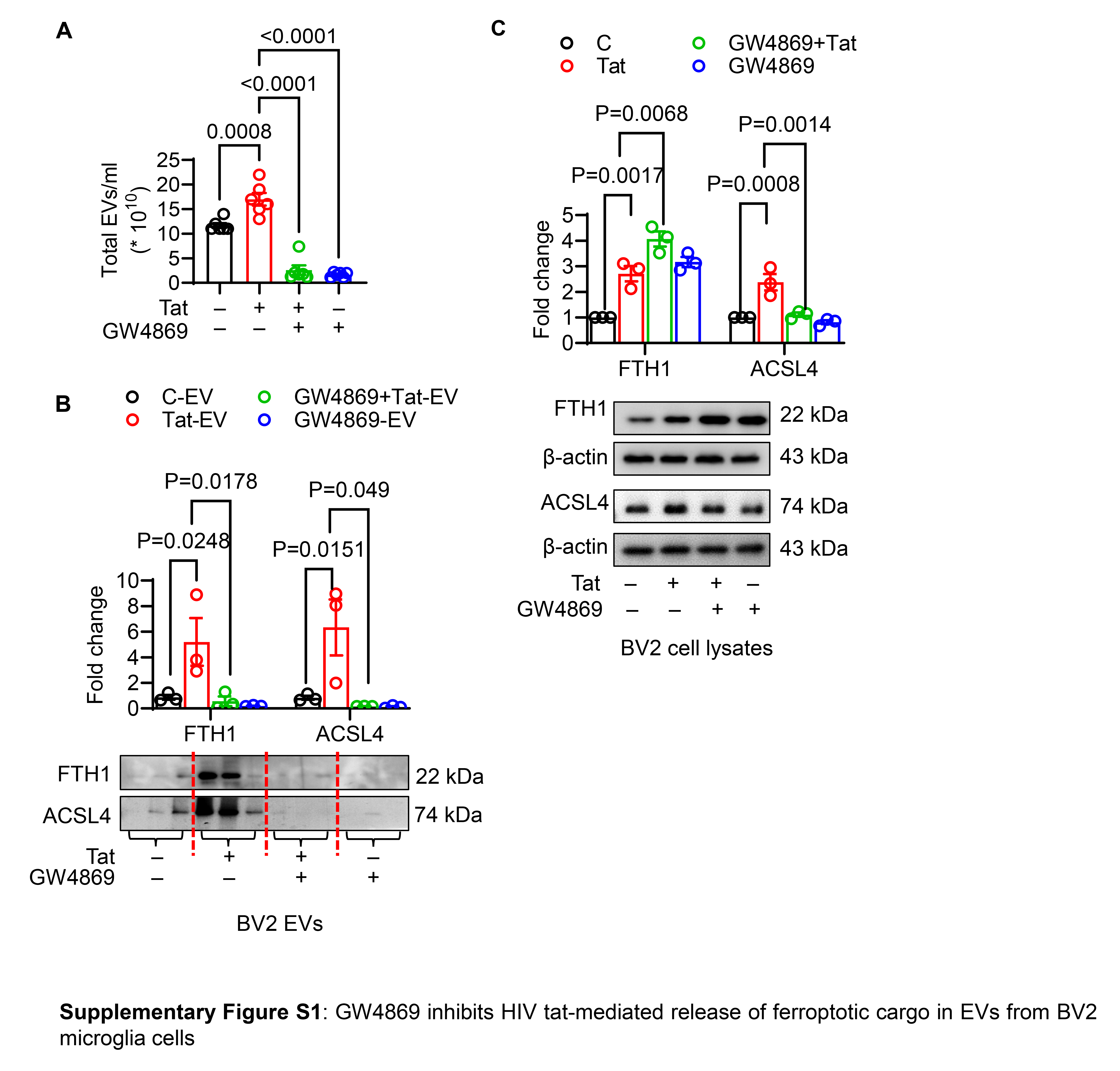

Supplement: Supplementary file 1 — Supporting Information: jex270153‐sup‐0001‐FigureS1.tif [file JEX2-5-e70153-s007.tif]

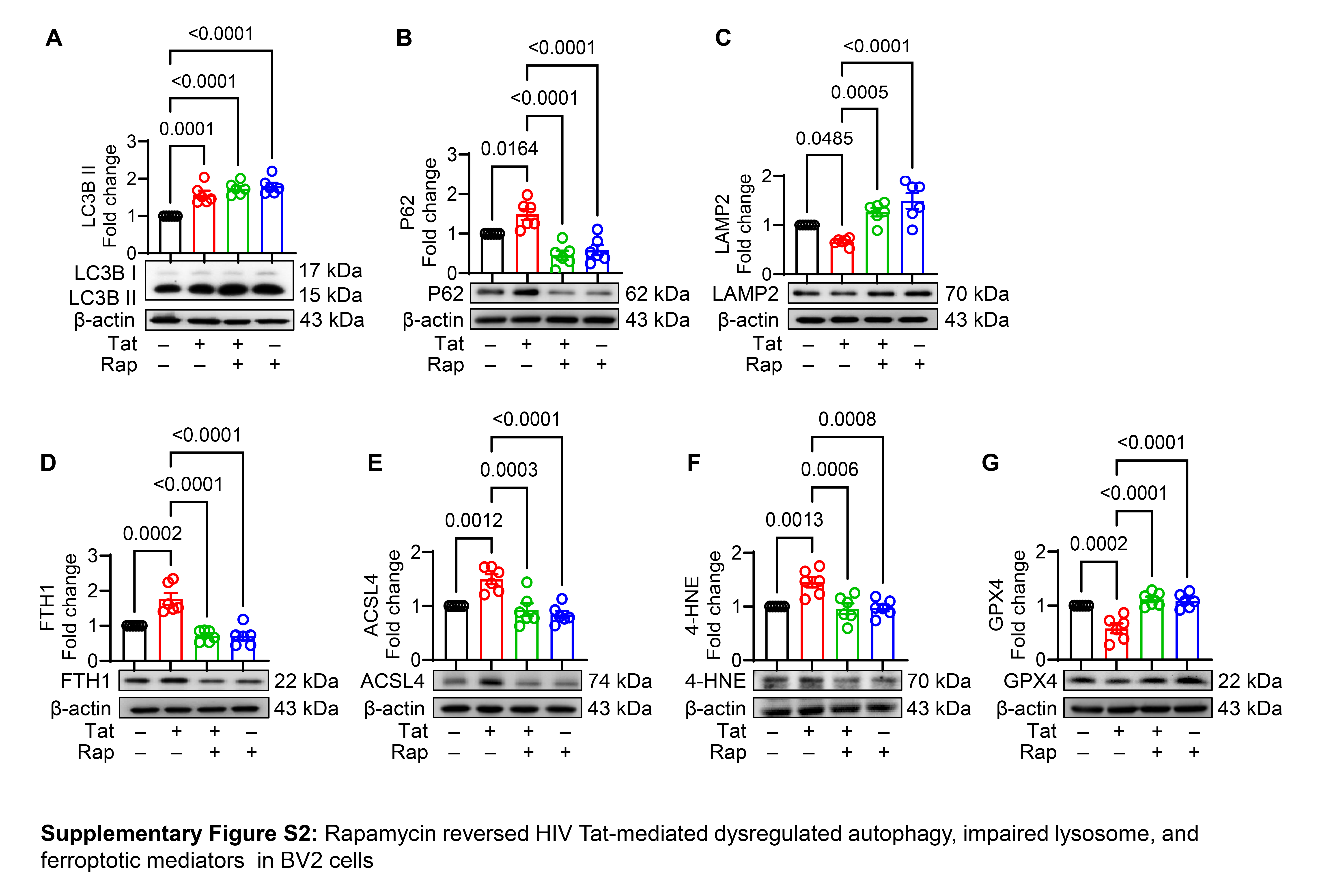

Supplement: Supplementary file 2 — Supporting Information: jex270153‐sup‐0002‐FigureS2.tif [file JEX2-5-e70153-s006.tif]

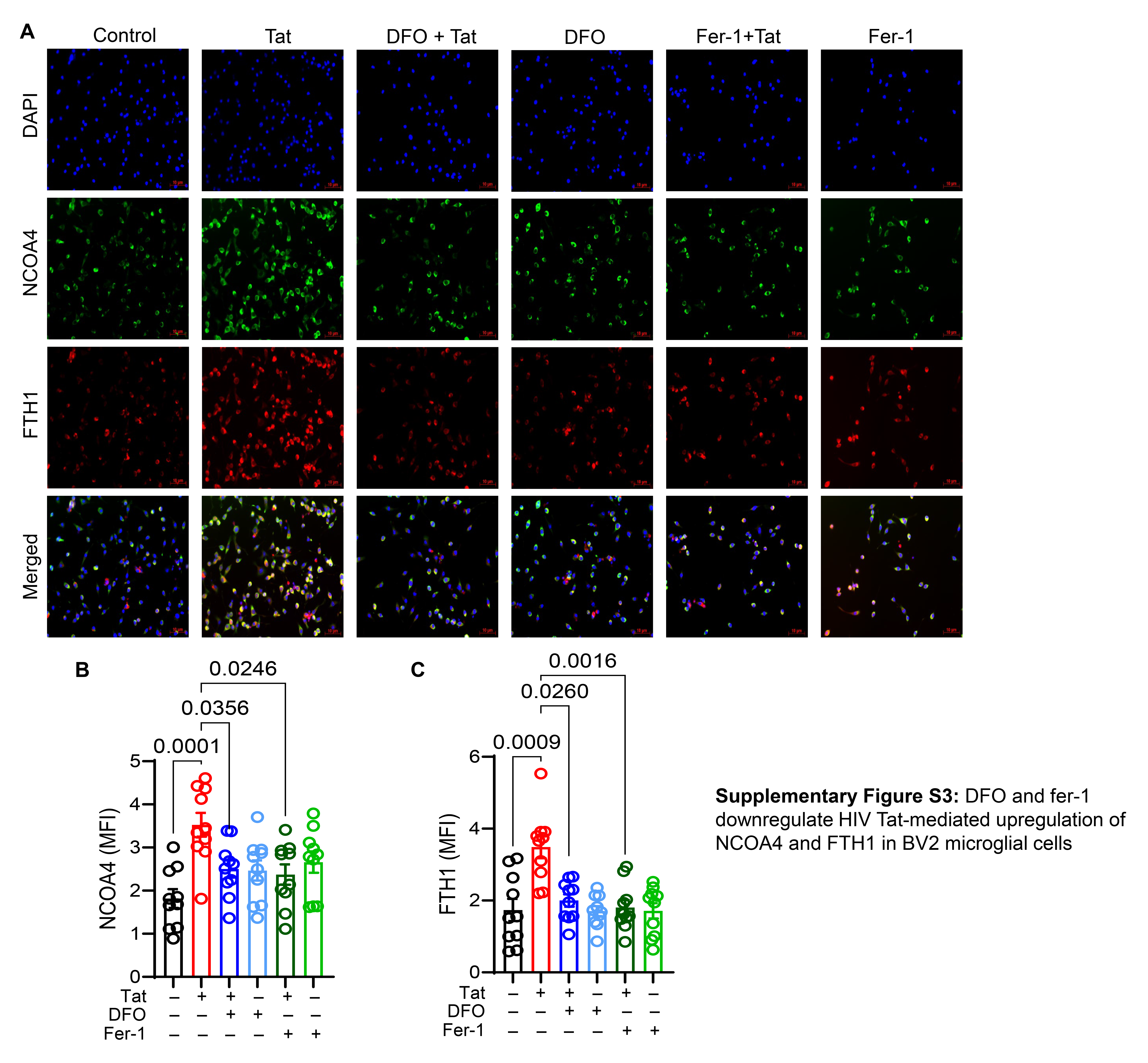

Supplement: Supplementary file 3 — Supporting Information: jex270153‐sup‐0003‐FigureS3.tif [file JEX2-5-e70153-s002.tif]

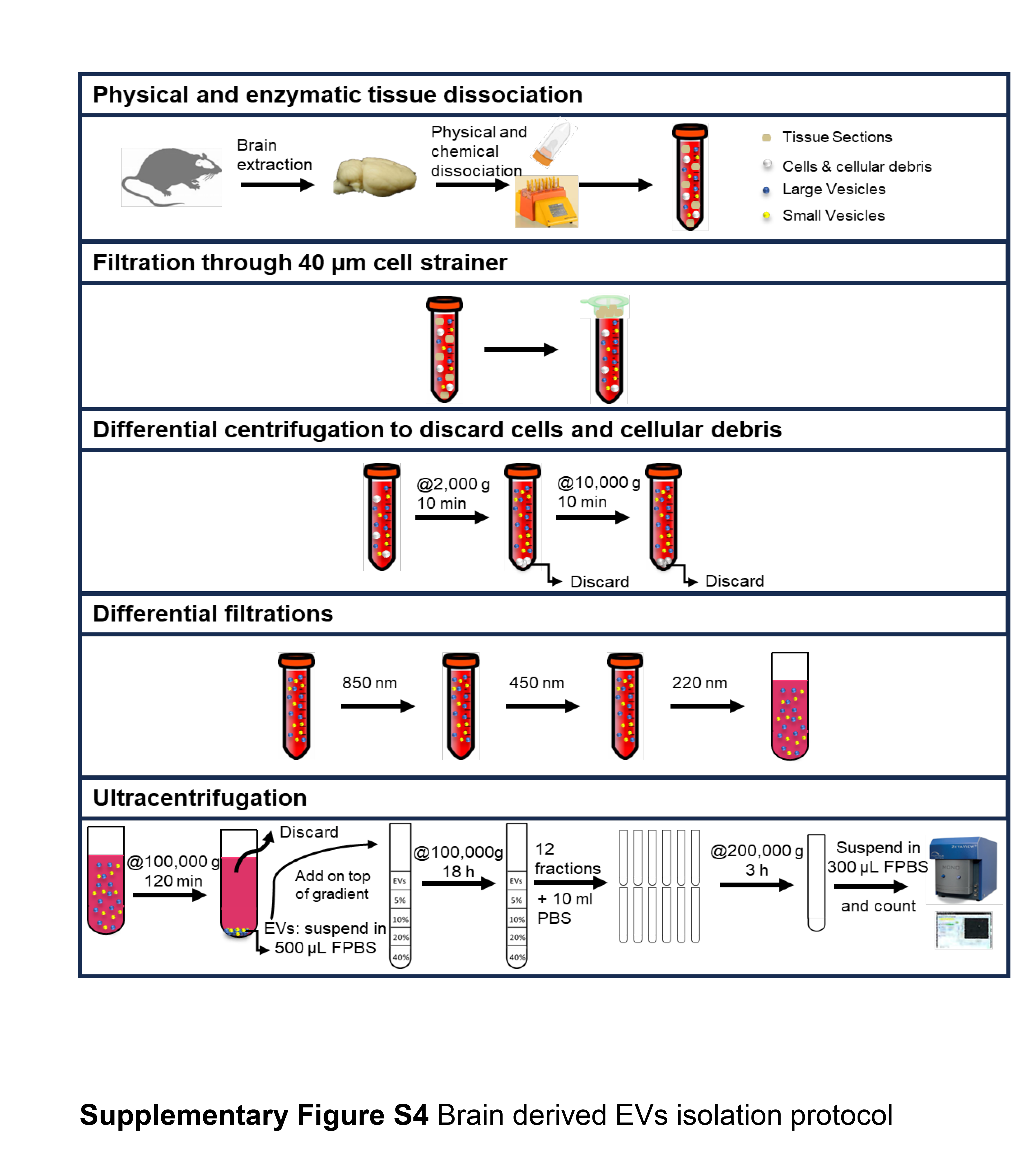

Supplement: Supplementary file 4 — Supporting Information: jex270153‐sup‐0004‐FigureS4.tif [file JEX2-5-e70153-s012.tif]

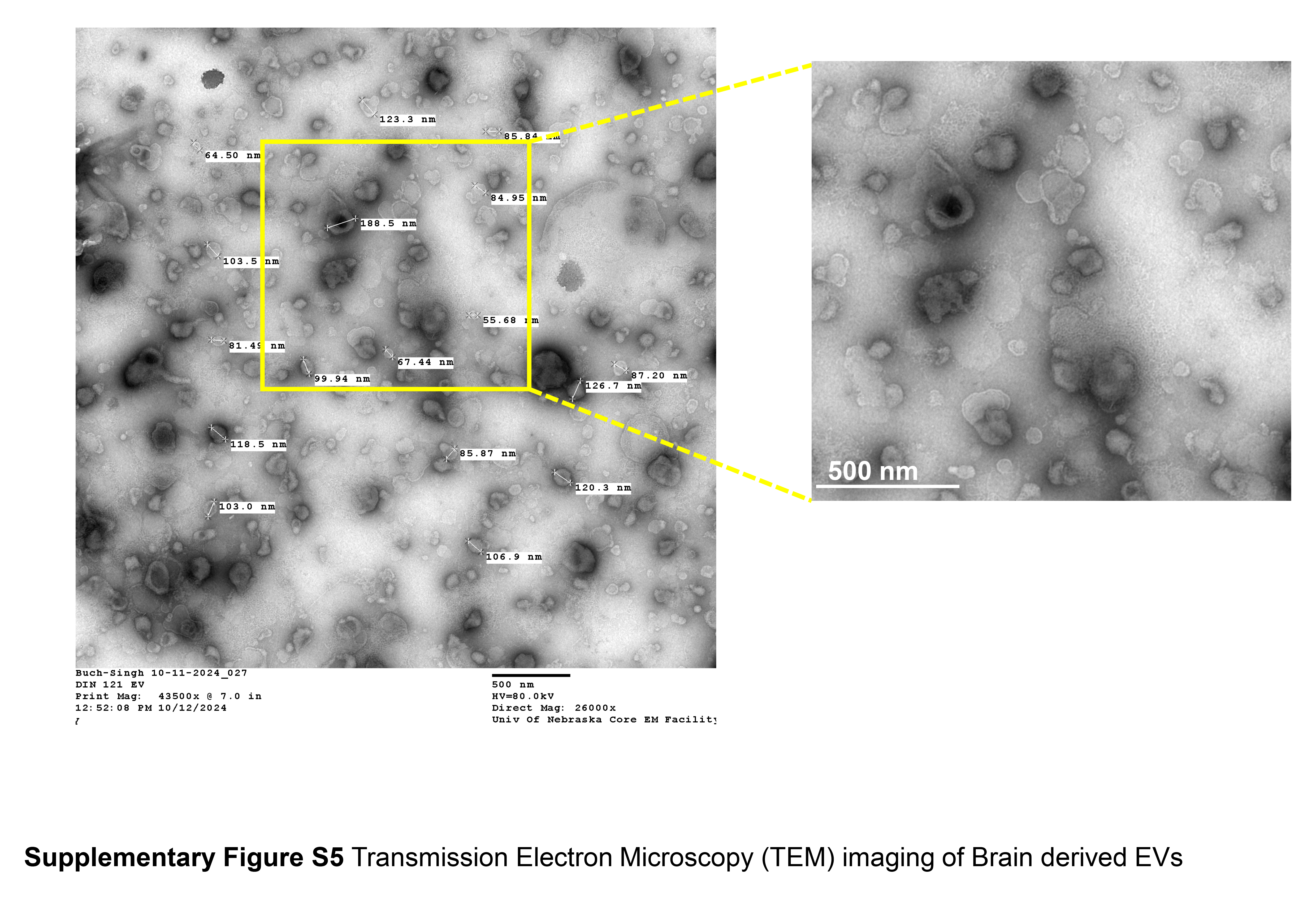

Supplement: Supplementary file 5 — Supporting Information: jex270153‐sup‐0005‐FigureS5.tif [file JEX2-5-e70153-s003.tif]

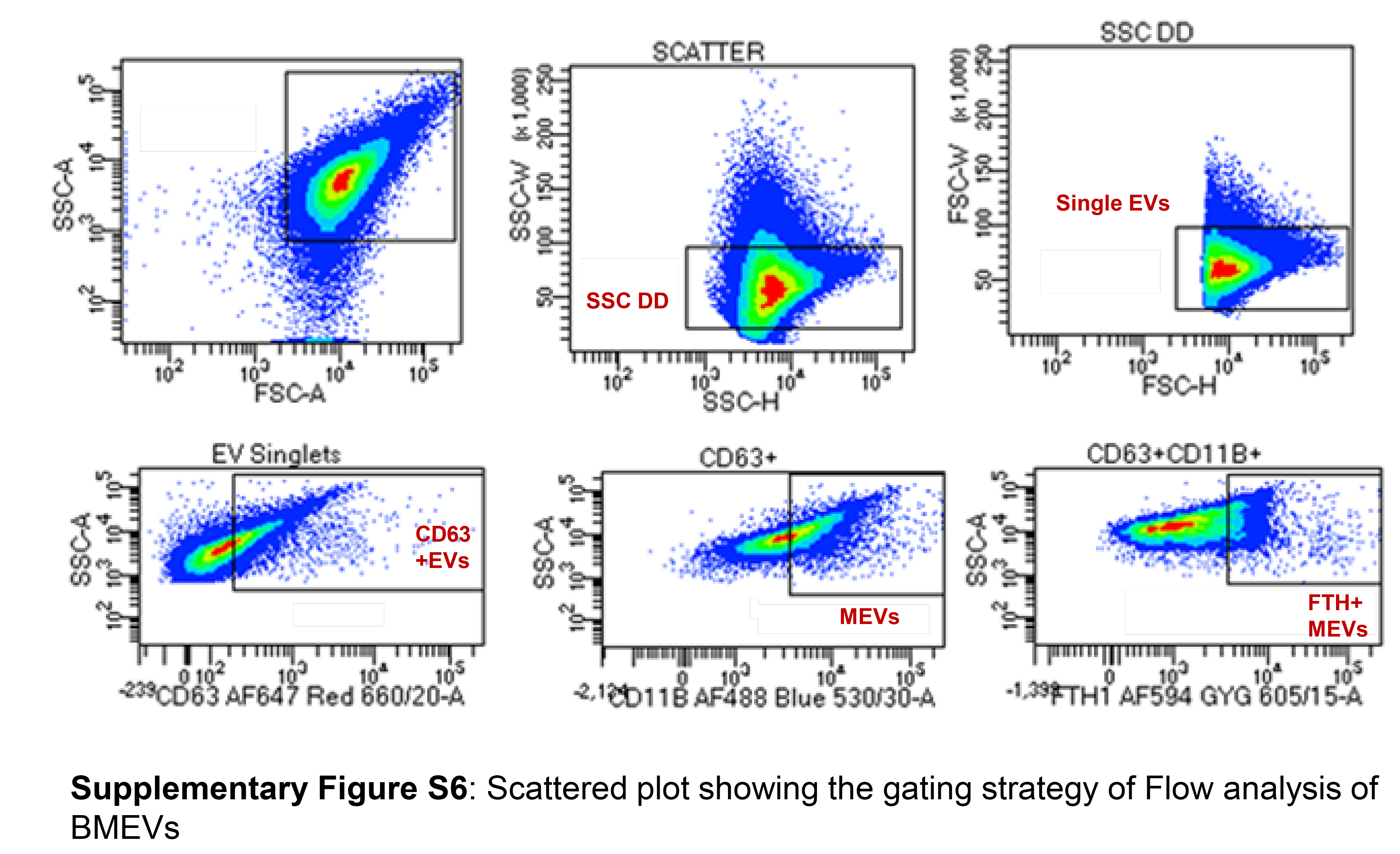

Supplement: Supplementary file 6 — Supporting Information: jex270153‐sup‐0006‐FigureS6.tif [file JEX2-5-e70153-s005.tif]

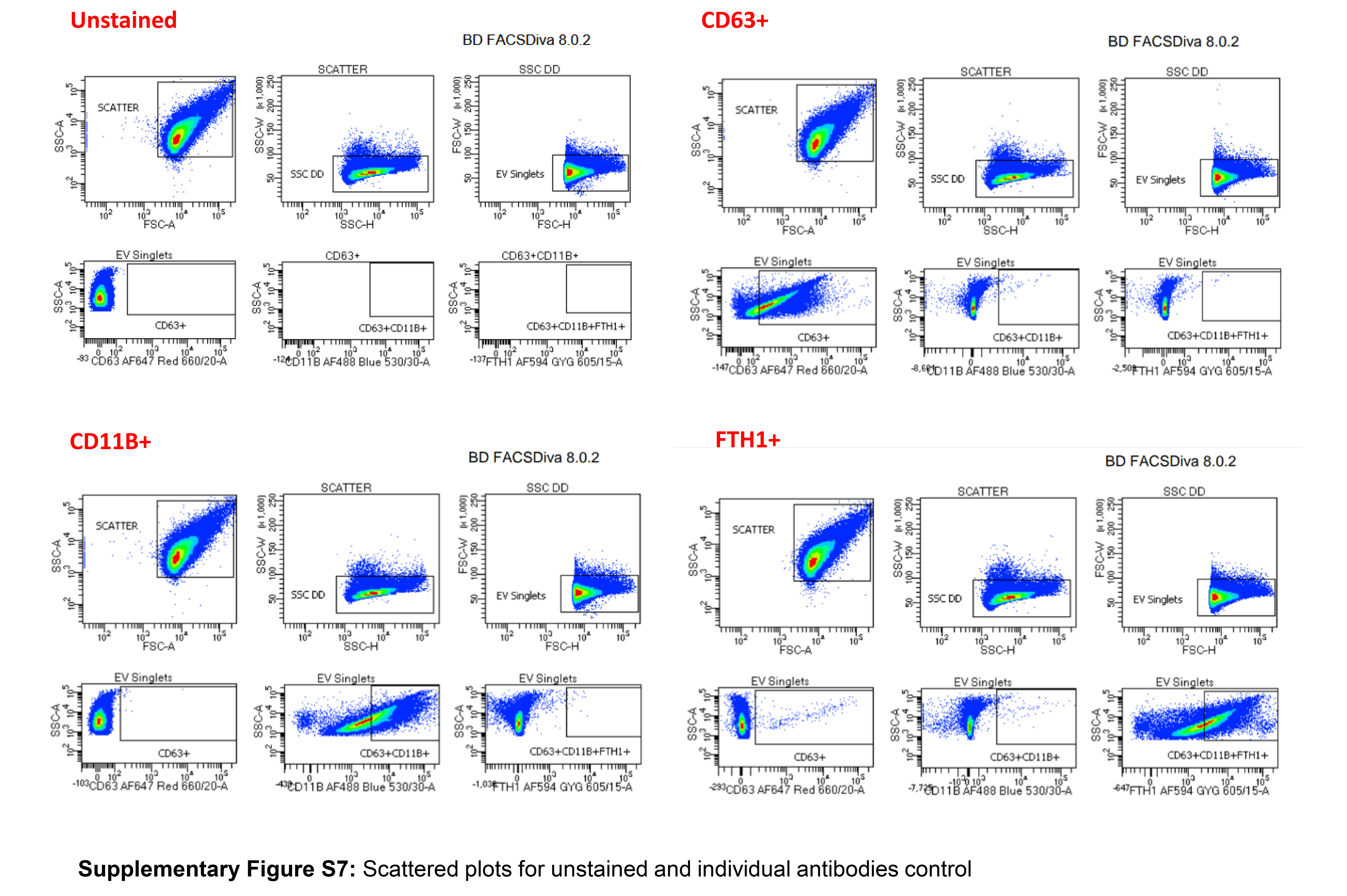

Supplement: Supplementary file 7 — Supporting Information: jex270153‐sup‐0007‐FigureS7.tif [file JEX2-5-e70153-s011.tif]

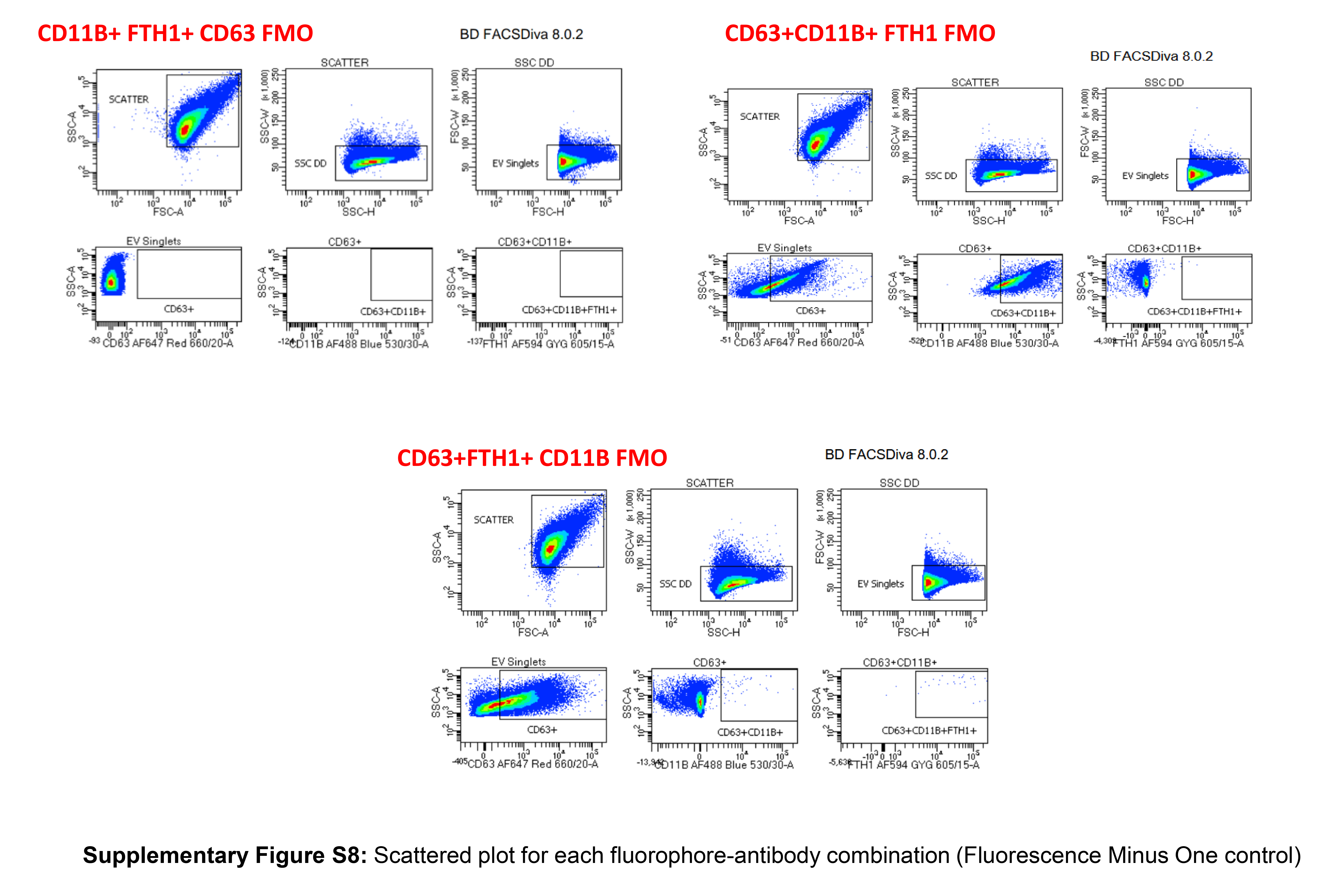

Supplement: Supplementary file 8 — Supporting Information: jex270153‐sup‐0008‐FigureS8.tif [file JEX2-5-e70153-s001.tif]

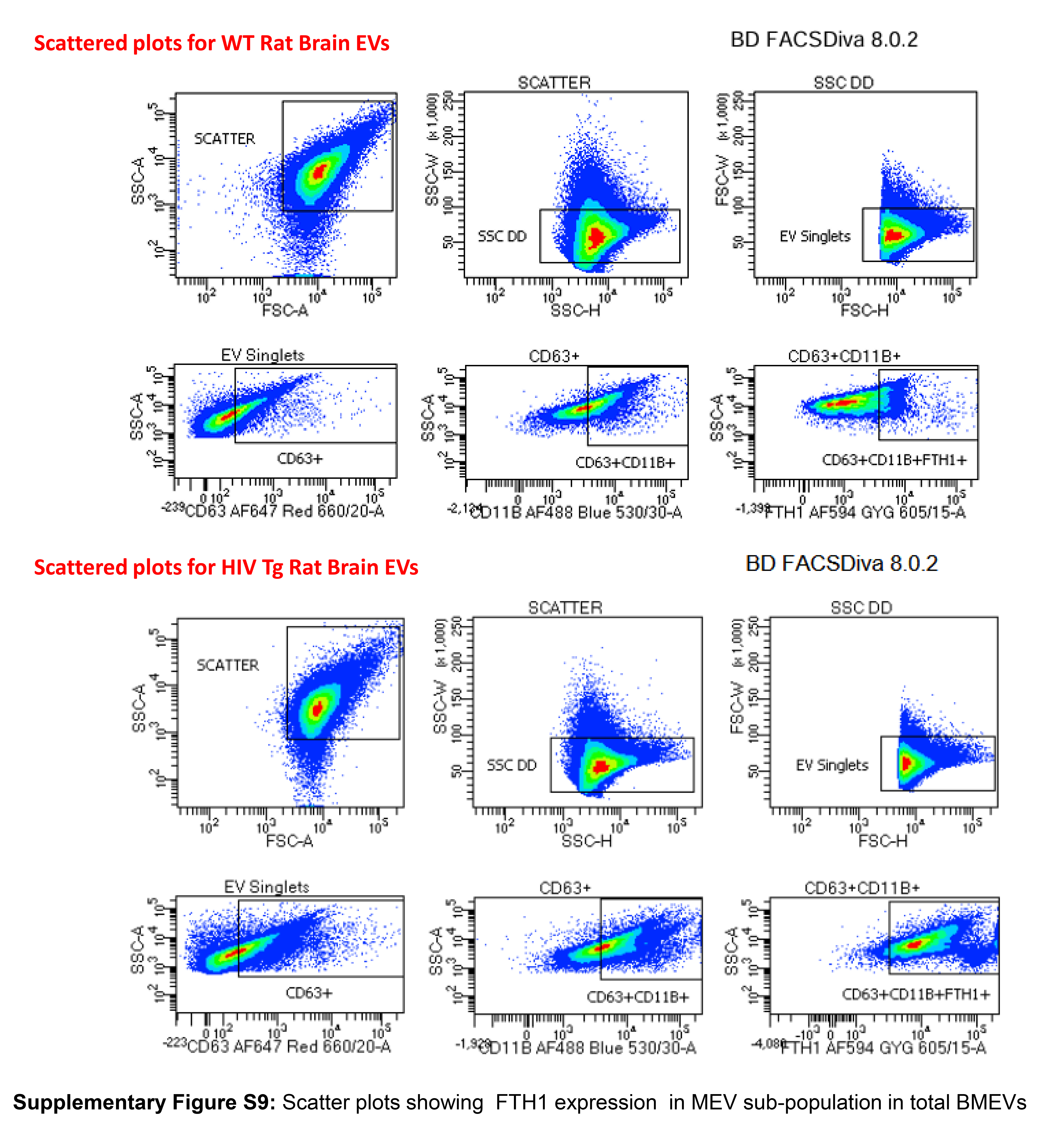

Supplement: Supplementary file 9 — Supporting Information: jex270153‐sup‐0009‐FigureS9.tif [file JEX2-5-e70153-s008.tif]

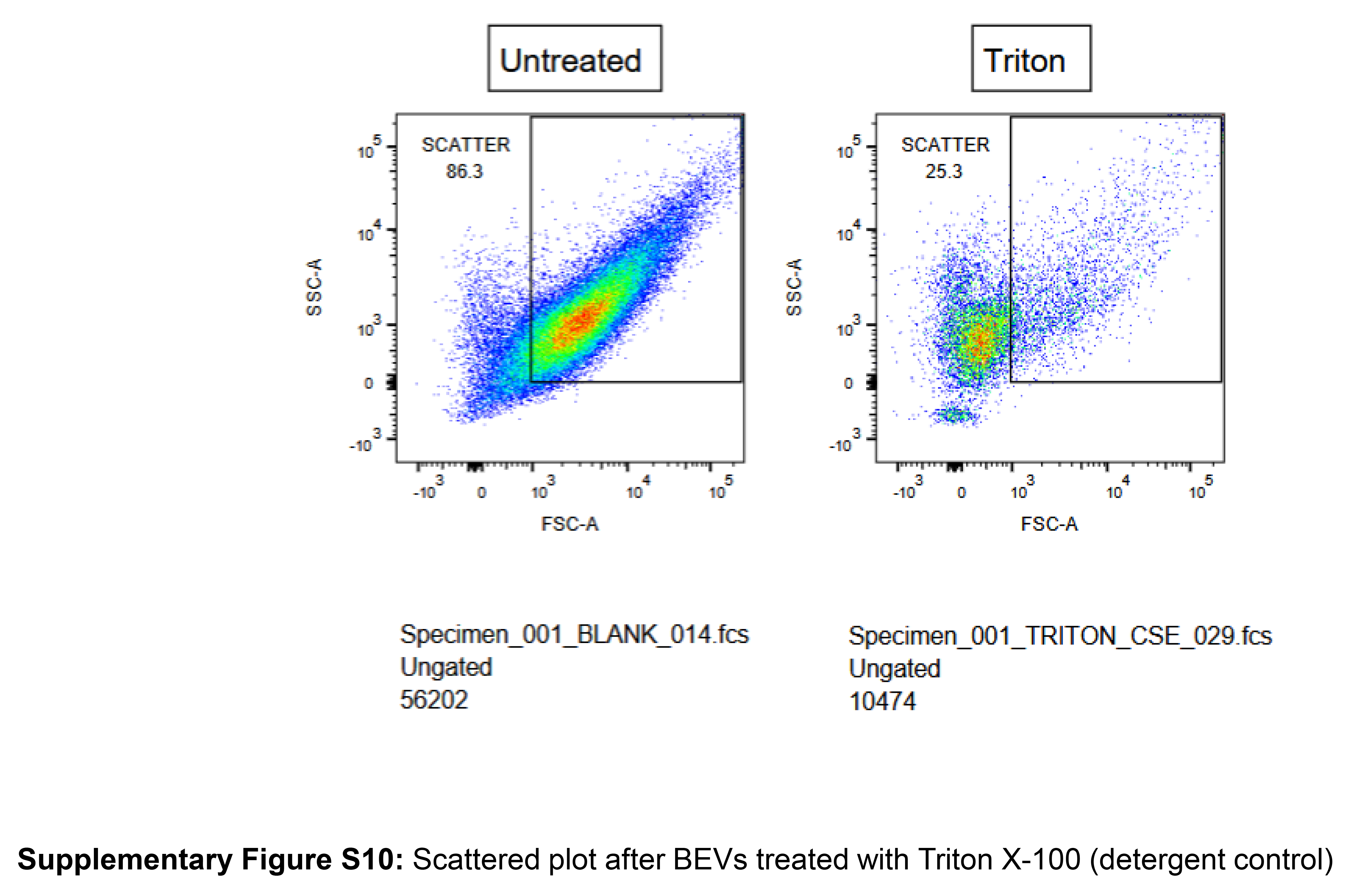

Supplement: Supplementary file 10 — Supporting Information: jex270153‐sup‐0010‐FigureS10.tif [file JEX2-5-e70153-s004.tif]
