# Supplementary material for: HIV Tat‐Stimulated Microglial Extracellular Vesicles Are Enriched for Ferroptosis Mediators: Role of Dysregulated Autophagy
Source: J Extracell Biol. 2026 Jun 11;5(6):e70153. doi: 10.1002/jex2.70153 (PMC13257887; doi:10.1002/jex2.70153)
Supplement: Supplementary file 12 — Supporting Information: jex270153‐sup‐0012‐SuppMat.pdf [file JEX2-5-e70153-s009.pdf]

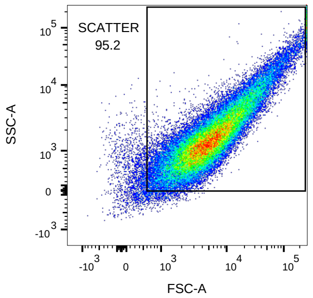

DILUTION_001_DH_117_038.fcs
Ungated
55312

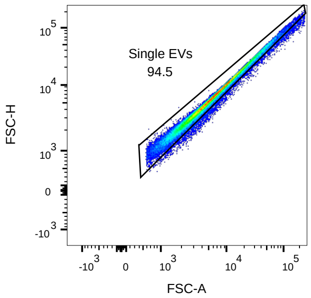

DILUTION_001_DH_117_038.fcs
SCATTER
52642

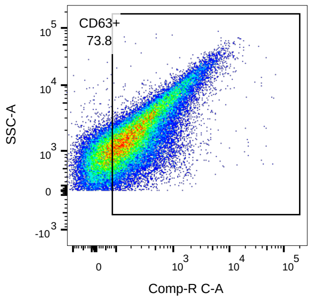

DILUTION_001_DH_117_038.fcs
Single EVs
49770

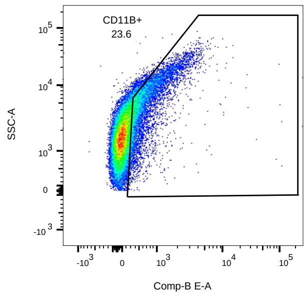

DILUTION_001_DH_117_038.fcs
CD63+
36726

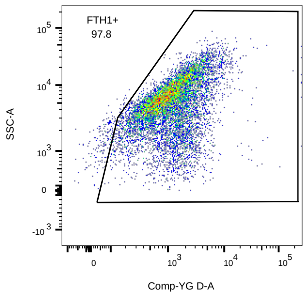

DILUTION_001_DH_117_038.fcs
CD11B+
8675

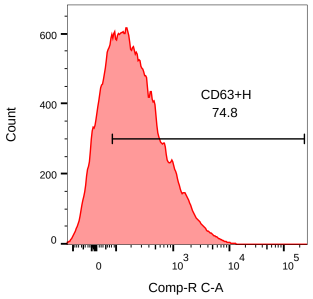

DILUTION_001_DH_117_038.fcs
Single EVs
49770

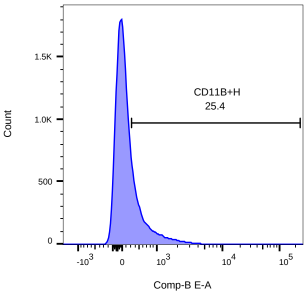

DILUTION_001_DH_117_038.fcs
CD63+H
37245

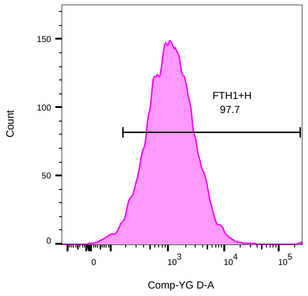

DILUTION_001_DH_117_038.fcs
CD11B+H
9464

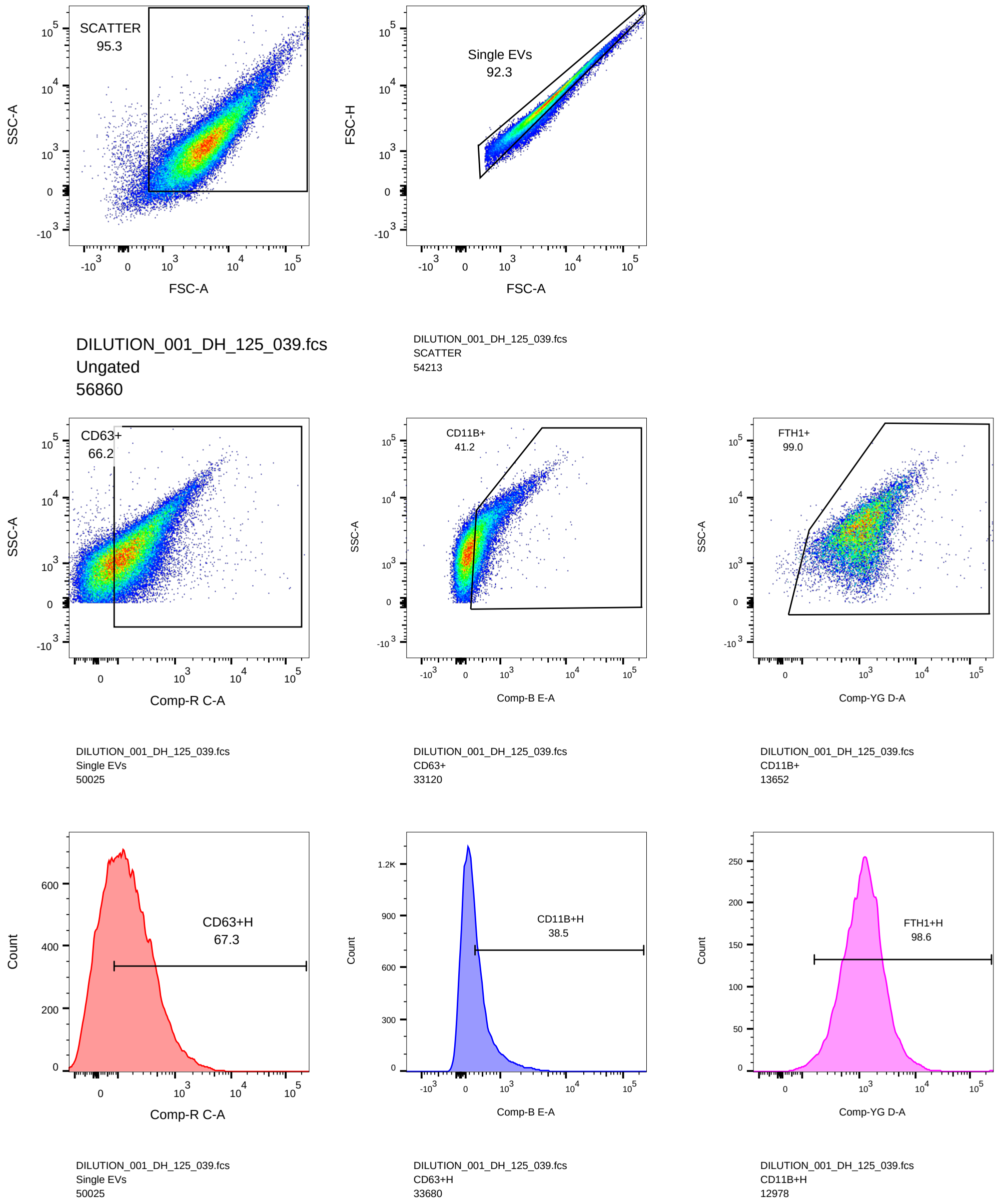
SCATTER
95.3
SSC-A
FSC-A
Single EVs
92.3
FSC-H
FSC-A
DILUTION_001_DH_125_039.fcs
Ungated
56860
DILUTION_001_DH_125_039.fcs
SCATTER
54213
CD63+
66.2
SSC-A
Comp-R C-A
CD11B+
41.2
SSC-A
Comp-B E-A
FTH1+
99.0
SSC-A
Comp-YG D-A
DILUTION_001_DH_125_039.fcs
Single EVs
50025
DILUTION_001_DH_125_039.fcs
CD63+
33120
DILUTION_001_DH_125_039.fcs
CD11B+
13652
CD63+H
67.3
Count
Comp-R C-A
CD11B+H
38.5
Count
Comp-B E-A
FTH1+H
98.6
Count
Comp-YG D-A
DILUTION_001_DH_125_039.fcs
Single EVs
50025
DILUTION_001_DH_125_039.fcs
CD63+H
33680
DILUTION_001_DH_125_039.fcs
CD11B+H
12978

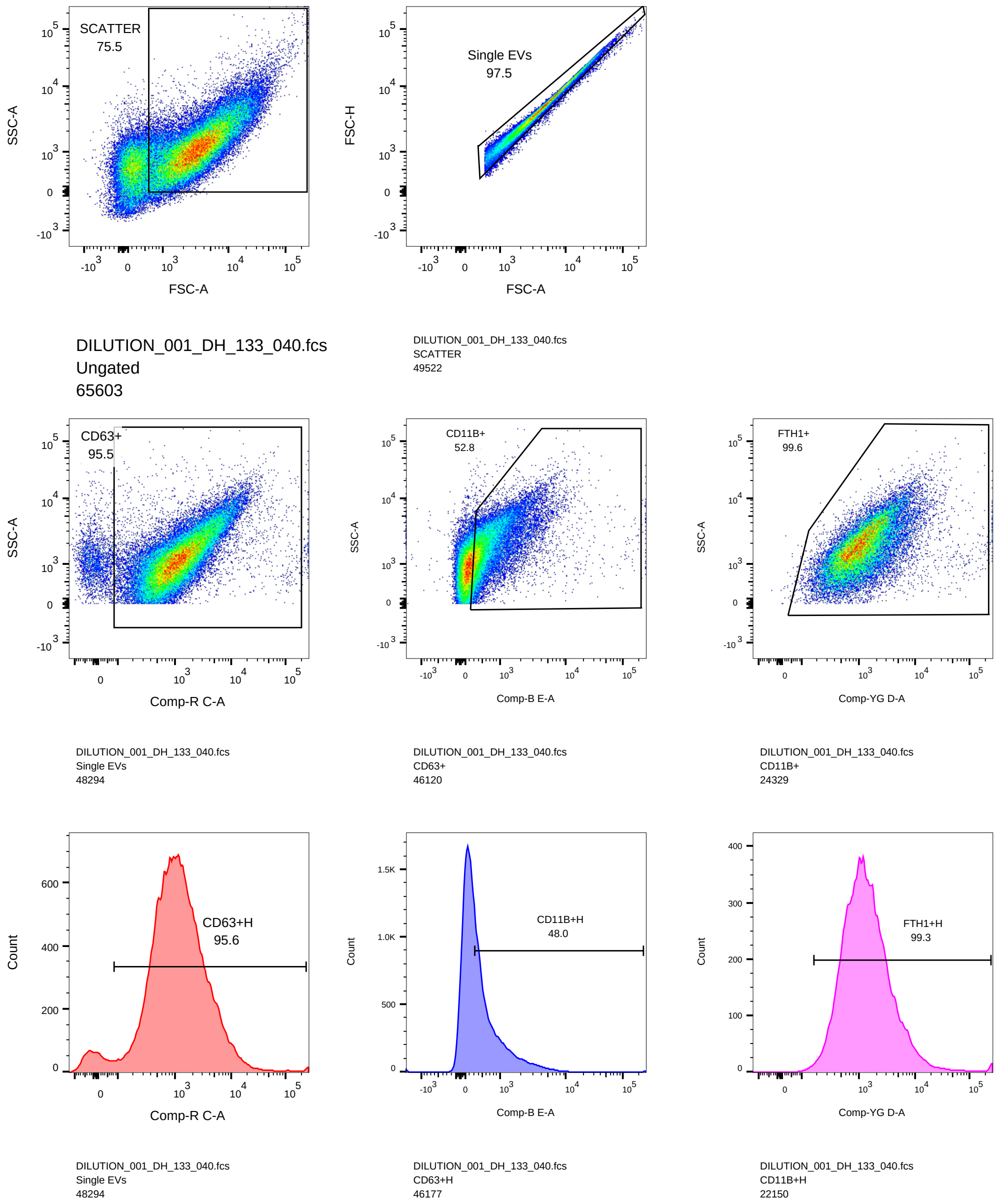
SCATTER
75.5
SSC-A
FSC-A
Single EVs
97.5
FSC-H
FSC-A
DILUTION_001_DH_133_040.fcs
Ungated
65603
DILUTION_001_DH_133_040.fcs
SCATTER
49522
CD63+
95.5
SSC-A
Comp-R C-A
CD11B+
52.8
SSC-A
Comp-B E-A
FTH1+
99.6
SSC-A
Comp-YG D-A
DILUTION_001_DH_133_040.fcs
Single EVs
48294
DILUTION_001_DH_133_040.fcs
CD63+
46120
DILUTION_001_DH_133_040.fcs
CD11B+
24329
CD63+H
95.6
Count
Comp-R C-A
CD11B+H
48.0
Count
Comp-B E-A
FTH1+H
99.3
Count
Comp-YG D-A
DILUTION_001_DH_133_040.fcs
Single EVs
48294
DILUTION_001_DH_133_040.fcs
CD63+H
46177
DILUTION_001_DH_133_040.fcs
CD11B+H
22150

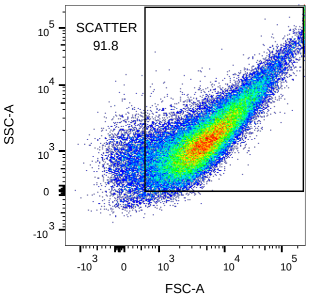

DILUTION_001_DW_86_036.fcs
Ungated
56395

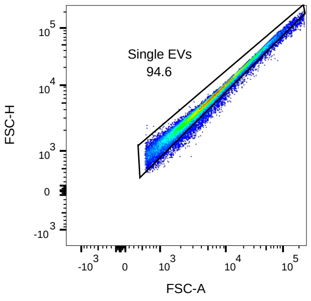

DILUTION_001_DW_86_036.fcs
SCATTER
51780

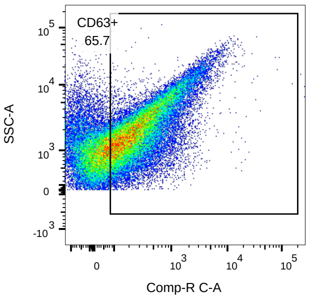

DILUTION_001_DW_86_036.fcs
Single EVs
48980

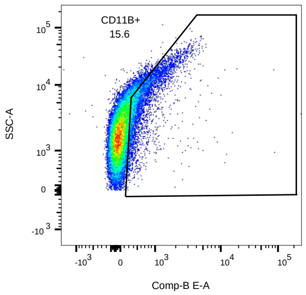

DILUTION_001_DW_86_036.fcs
CD63+
32201

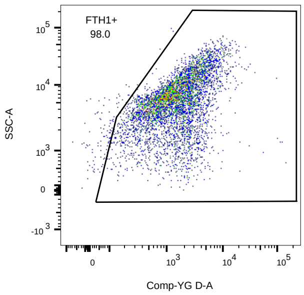

DILUTION_001_DW_86_036.fcs
CD11B+
5031

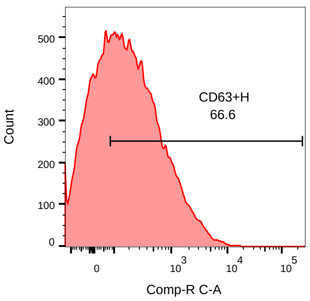

DILUTION_001_DW_86_036.fcs
Single EVs
48980

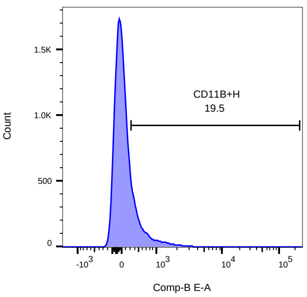

DILUTION_001_DW_86_036.fcs
CD63+H
32613

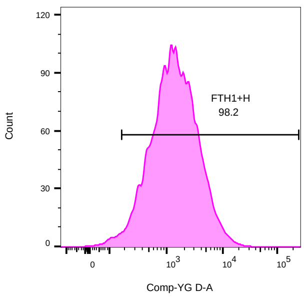

DILUTION_001_DW_86_036.fcs
CD11B+H
6373

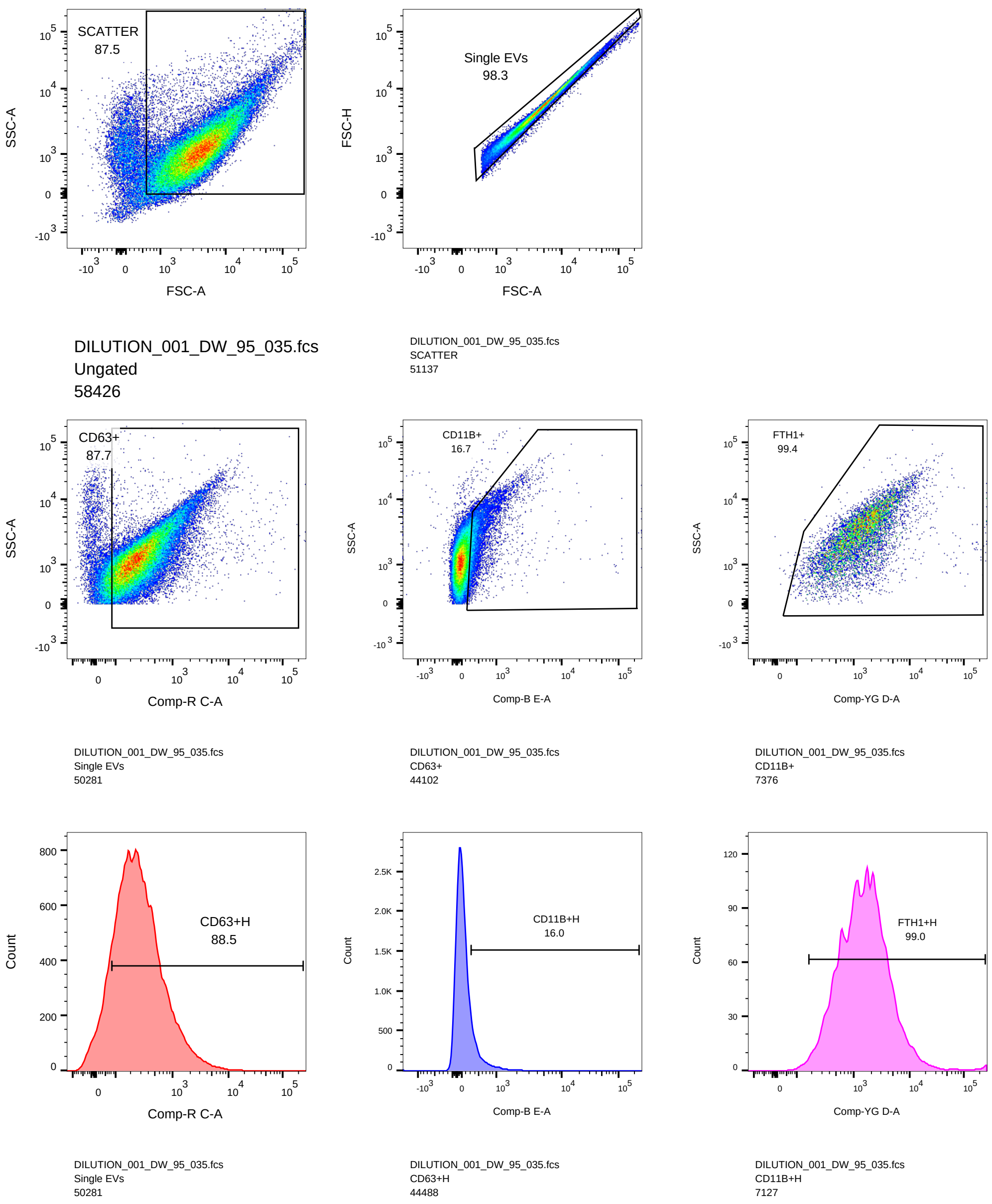

SCATTER
87.5
SSC-A
FSC-A
Single EVs
98.3
FSC-H
FSC-A
DILUTION_001_DW_95_035.fcs
Ungated
58426
DILUTION_001_DW_95_035.fcs
SCATTER
51137
CD63+
87.7
SSC-A
Comp-R C-A
CD11B+
16.7
SSC-A
Comp-B E-A
FTH1+
99.4
SSC-A
Comp-YG D-A
DILUTION_001_DW_95_035.fcs
Single EVs
50281
DILUTION_001_DW_95_035.fcs
CD63+
44102
DILUTION_001_DW_95_035.fcs
CD11B+
7376
CD63+H
88.5
Count
Comp-R C-A
CD11B+H
16.0
Count
Comp-B E-A
FTH1+H
99.0
Count
Comp-YG D-A
DILUTION_001_DW_95_035.fcs
Single EVs
50281
DILUTION_001_DW_95_035.fcs
CD63+H
44488
DILUTION_001_DW_95_035.fcs
CD11B+H
7127

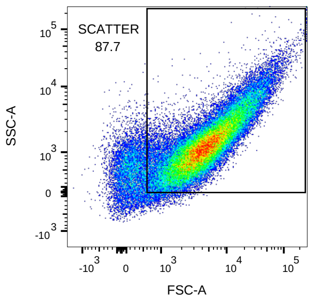

DILUTION_001_DW_121_037.fcs
Ungated
58537

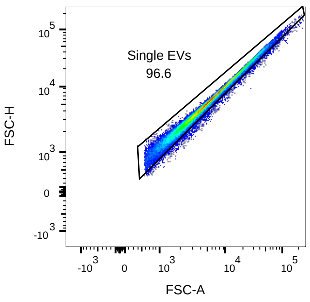

DILUTION_001_DW_121_037.fcs
SCATTER
51327

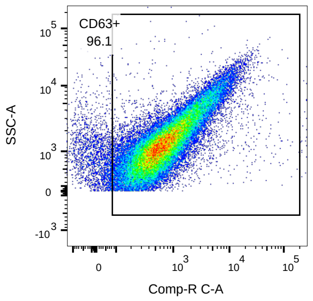

DILUTION_001_DW_121_037.fcs
Single EVs
49582

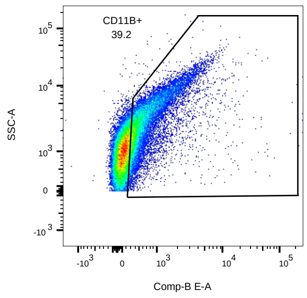

DILUTION_001_DW_121_037.fcs
CD63+
47644

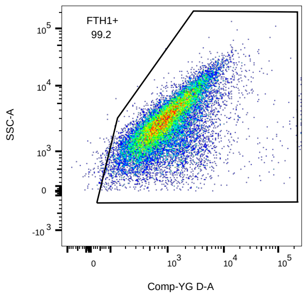

DILUTION_001_DW_121_037.fcs
CD11B+
18656

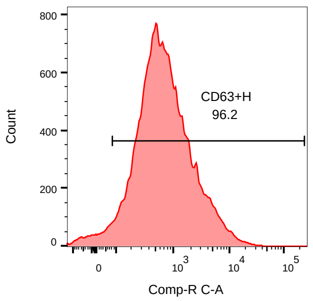

DILUTION_001_DW_121_037.fcs
Single EVs
49582

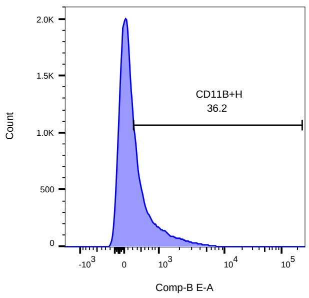

DILUTION_001_DW_121_037.fcs
CD63+H
47714

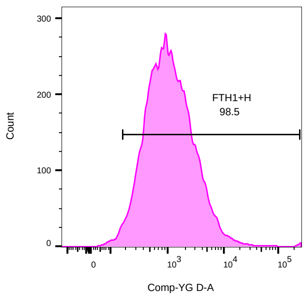

DILUTION_001_DW_121_037.fcs
CD11B+H
17257

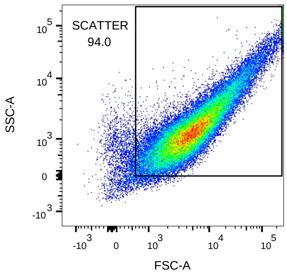

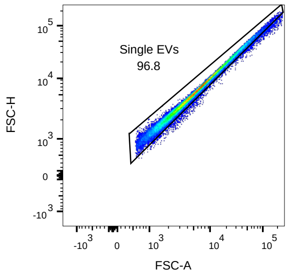

DILUTION_002_DH_117_044.fcs
Ungated
55173

DILUTION_002_DH_117_044.fcs
SCATTER
51842

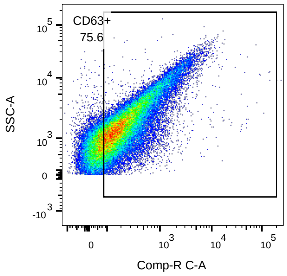

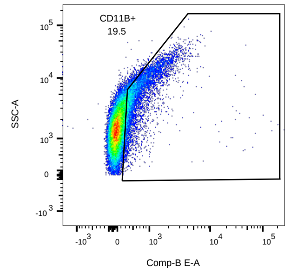

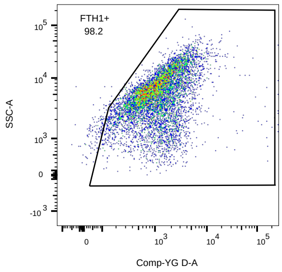

DILUTION_002_DH_117_044.fcs
Single EVs
50171

DILUTION_002_DH_117_044.fcs
CD63+
37941

DILUTION_002_DH_117_044.fcs
CD11B+
7403

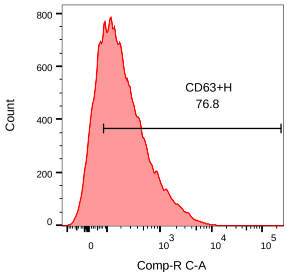

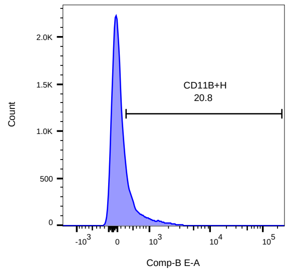

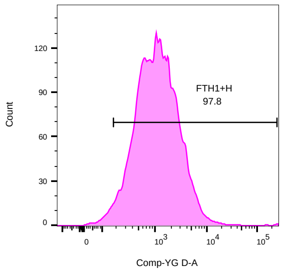

DILUTION_002_DH_117_044.fcs
Single EVs
50171

DILUTION_002_DH_117_044.fcs
CD63+H
38551

DILUTION_002_DH_117_044.fcs
CD11B+H
8007

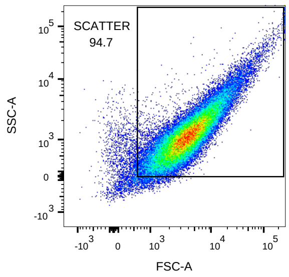

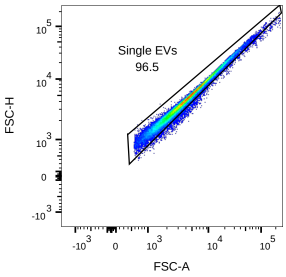

DILUTION_002_DH_125_045.fcs
Ungated
55508

DILUTION_002_DH_125_045.fcs
SCATTER
52574

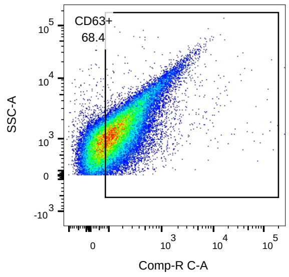

DILUTION_002_DH_125_045.fcs
Single EVs
50721

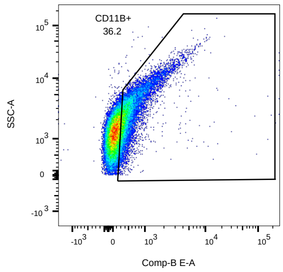

DILUTION_002_DH_125_045.fcs
CD63+
34685

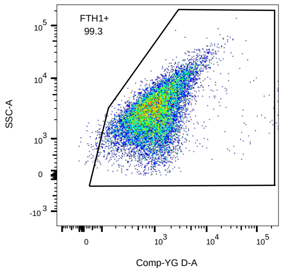

DILUTION_002_DH_125_045.fcs
CD11B+
12559

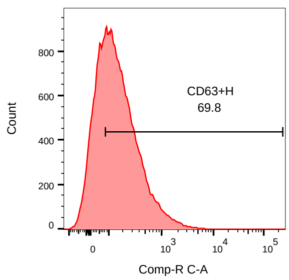

DILUTION_002_DH_125_045.fcs
Single EVs
50721

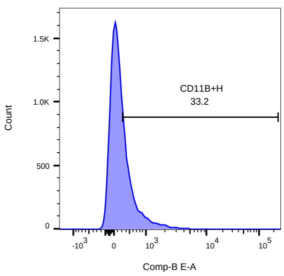

DILUTION_002_DH_125_045.fcs
CD63+H
35426

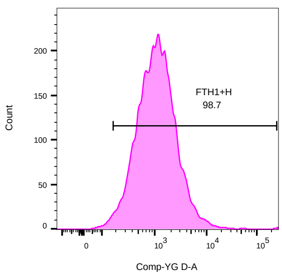

DILUTION_002_DH_125_045.fcs
CD11B+H
11779

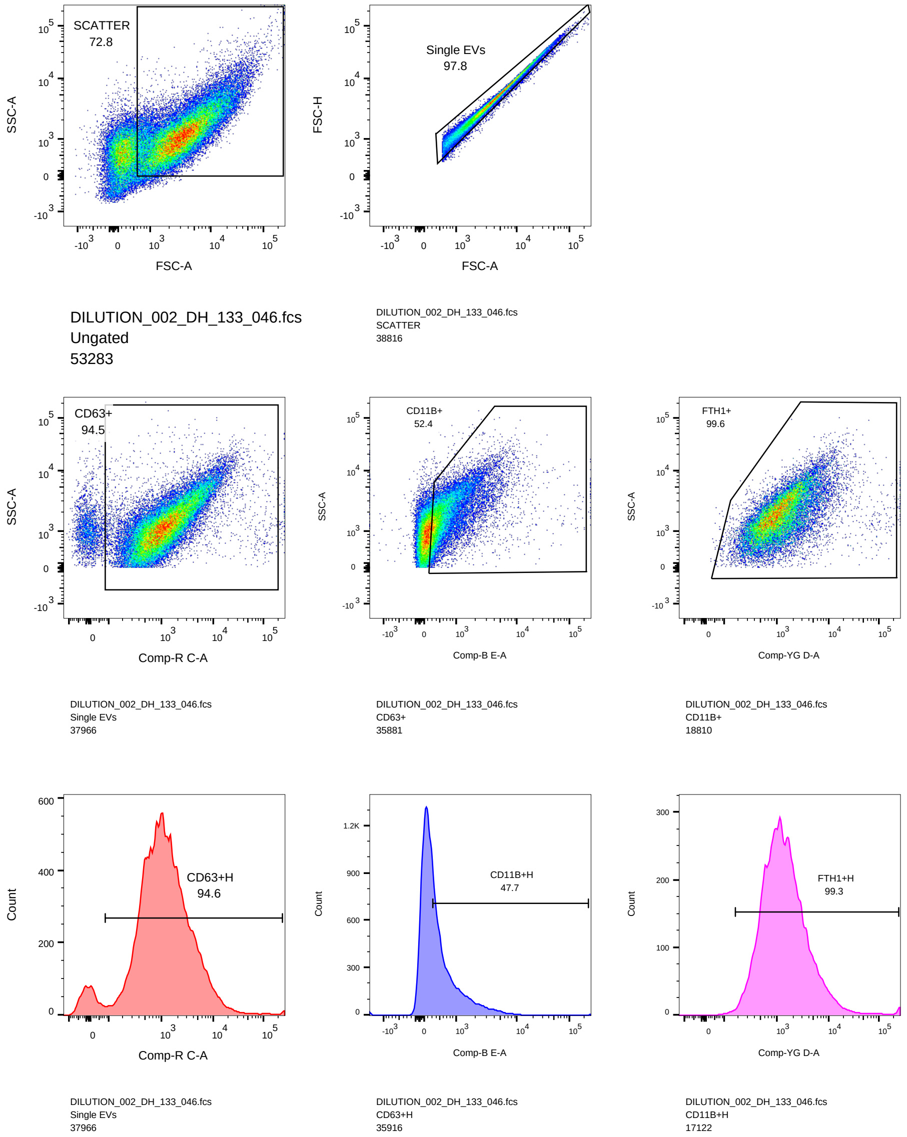
SCATTER
72.8
SSC-A
FSC-A
Single EVs
97.8
FSC-H
FSC-A
DILUTION_002_DH_133_046.fcs
Ungated
53283
DILUTION_002_DH_133_046.fcs
SCATTER
38816
CD63+
94.5
SSC-A
Comp-R C-A
CD11B+
52.4
SSC-A
Comp-B E-A
FTH1+
99.6
SSC-A
Comp-YG D-A
DILUTION_002_DH_133_046.fcs
Single EVs
37966
DILUTION_002_DH_133_046.fcs
CD63+
35881
DILUTION_002_DH_133_046.fcs
CD11B+
18810
CD63+H
94.6
Count
Comp-R C-A
CD11B+H
47.7
Count
Comp-B E-A
FTH1+H
99.3
Count
Comp-YG D-A
DILUTION_002_DH_133_046.fcs
Single EVs
37966
DILUTION_002_DH_133_046.fcs
CD63+H
35916
DILUTION_002_DH_133_046.fcs
CD11B+H
17122

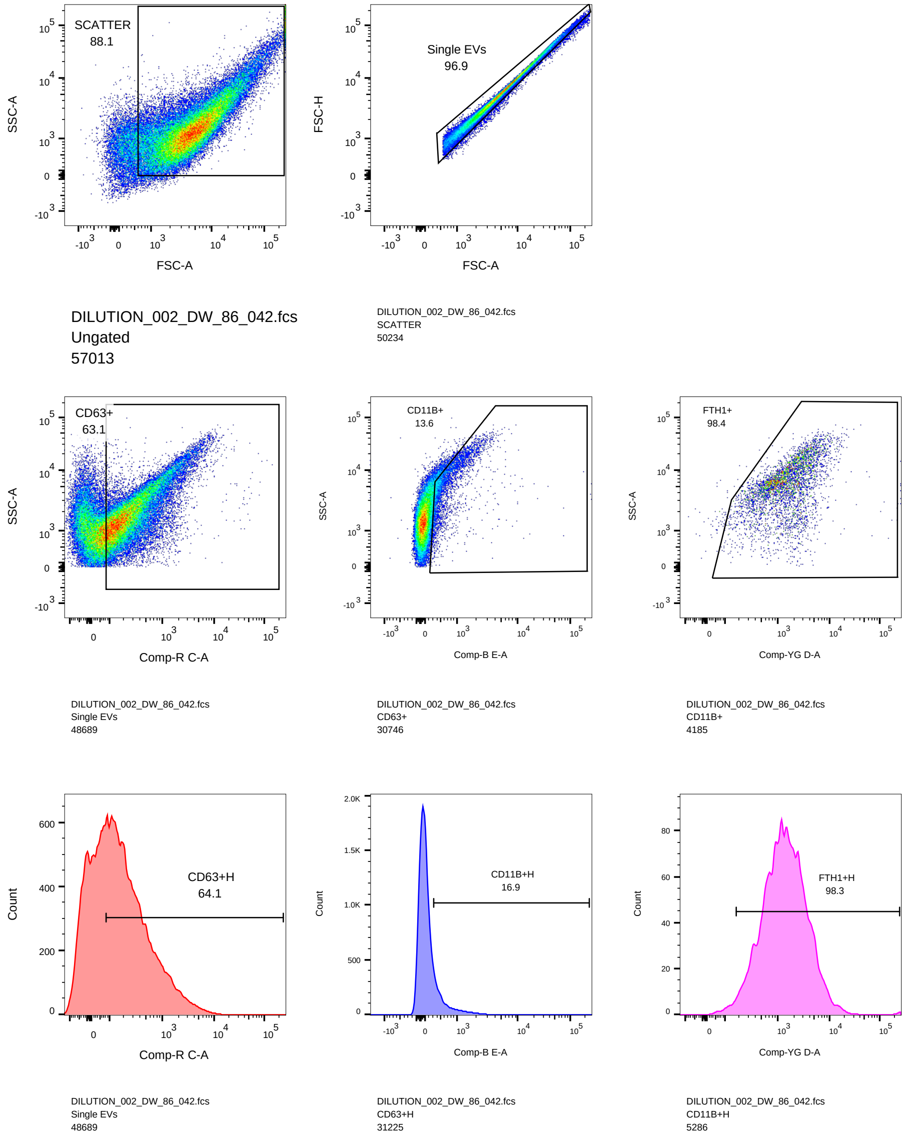
SCATTER
88.1
SSC-A
FSC-A
Single EVs
96.9
FSC-H
FSC-A
DILUTION_002_DW_86_042.fcs
Ungated
57013
DILUTION_002_DW_86_042.fcs
SCATTER
50234
CD63+
63.1
SSC-A
Comp-R C-A
CD11B+
13.6
SSC-A
Comp-B E-A
FTH1+
98.4
SSC-A
Comp-YG D-A
DILUTION_002_DW_86_042.fcs
Single EVs
48689
DILUTION_002_DW_86_042.fcs
CD63+
30746
DILUTION_002_DW_86_042.fcs
CD11B+
4185
CD63+H
64.1
Count
Comp-R C-A
CD11B+H
16.9
Count
Comp-B E-A
FTH1+H
98.3
Count
Comp-YG D-A
DILUTION_002_DW_86_042.fcs
Single EVs
48689
DILUTION_002_DW_86_042.fcs
CD63+H
31225
DILUTION_002_DW_86_042.fcs
CD11B+H
5286

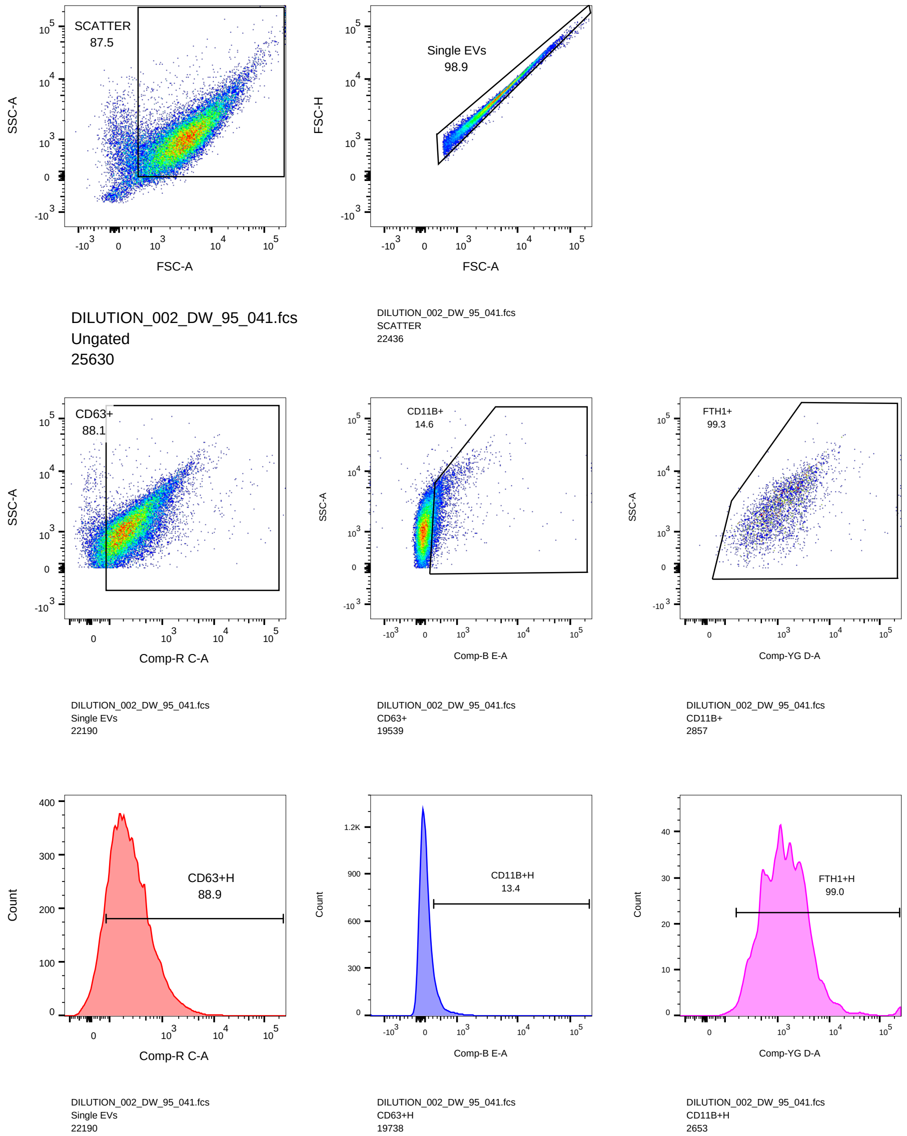
SCATTER
87.5
SSC-A
FSC-A
DILUTION_002_DW_95_041.fcs
Ungated
25630
Single EVs
98.9
FSC-H
FSC-A
DILUTION_002_DW_95_041.fcs
SCATTER
22436
CD63+
88.1
SSC-A
Comp-R C-A
DILUTION_002_DW_95_041.fcs
Single EVs
22190
CD11B+
14.6
SSC-A
Comp-B E-A
DILUTION_002_DW_95_041.fcs
CD63+
19539
FTH1+
99.3
SSC-A
Comp-YG D-A
DILUTION_002_DW_95_041.fcs
CD11B+
2857
CD63+H
88.9
Count
Comp-R C-A
DILUTION_002_DW_95_041.fcs
Single EVs
22190
CD11B+H
13.4
Count
Comp-B E-A
DILUTION_002_DW_95_041.fcs
CD63+H
19738
FTH1+H
99.0
Count
Comp-YG D-A
DILUTION_002_DW_95_041.fcs
CD11B+H
2653

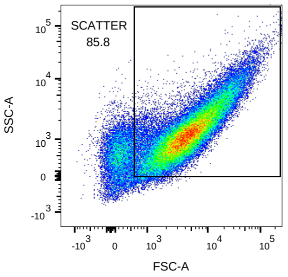

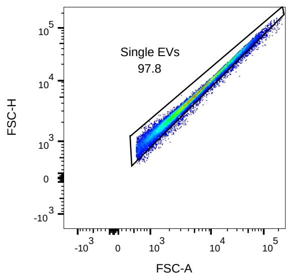

DILUTION_002_DW_121_043.fcs
Ungated
59229

DILUTION_002_DW_121_043.fcs
SCATTER
50832

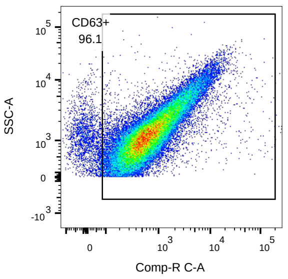

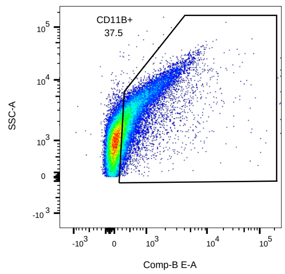

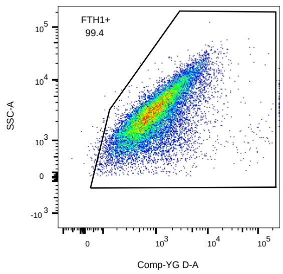

DILUTION_002_DW_121_043.fcs
Single EVs
49690

DILUTION_002_DW_121_043.fcs
CD63+
47750

DILUTION_002_DW_121_043.fcs
CD11B+
17916

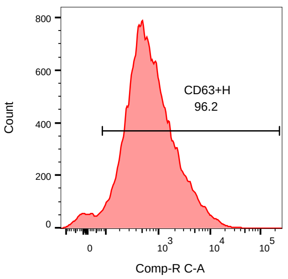

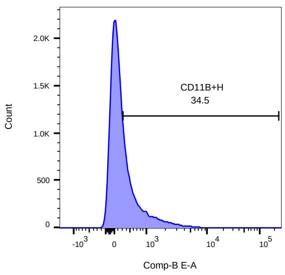

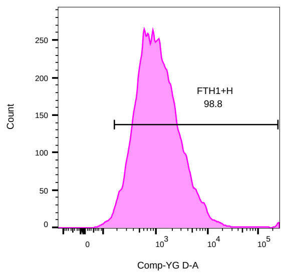

DILUTION_002_DW_121_043.fcs
Single EVs
49690

DILUTION_002_DW_121_043.fcs
CD63+H
47820

DILUTION_002_DW_121_043.fcs
CD11B+H
16506

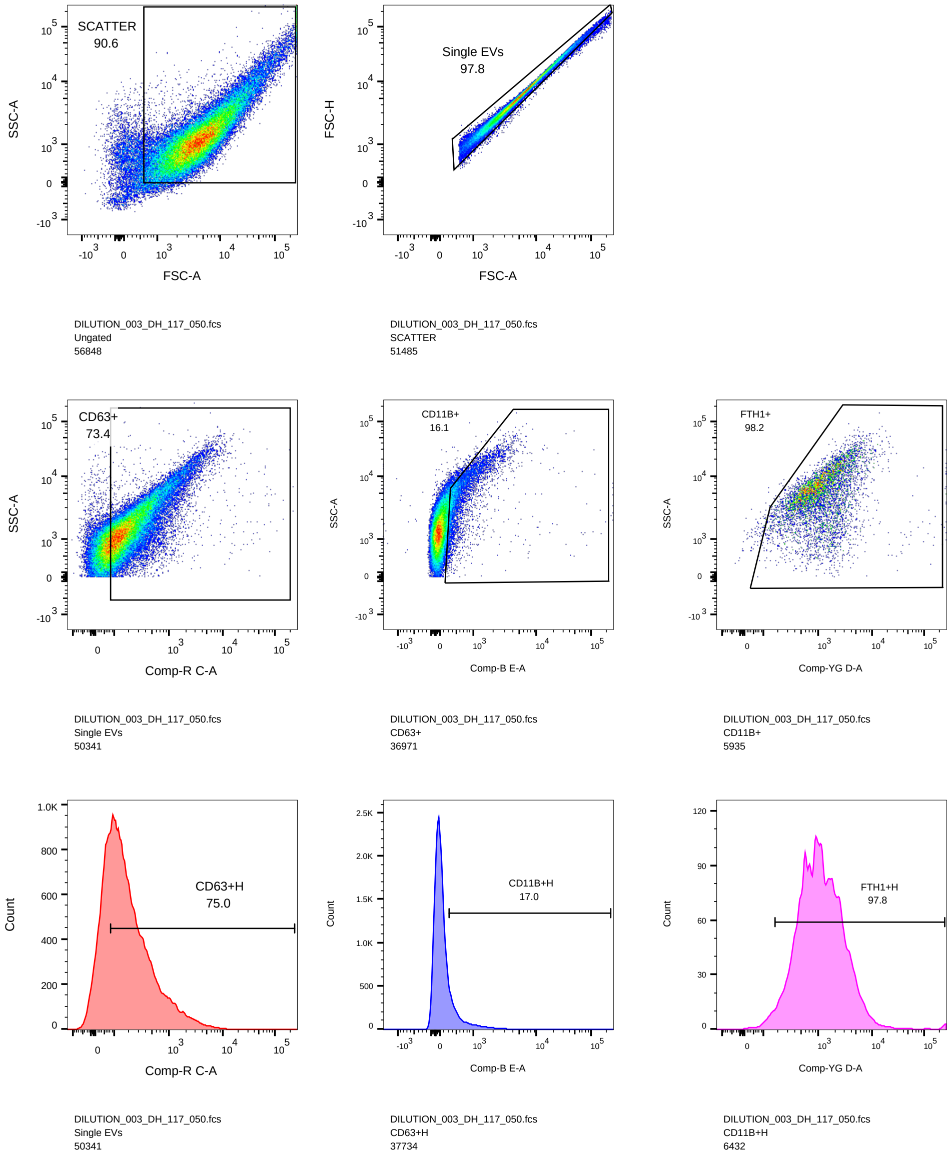
SCATTER
90.6
SSC-A
FSC-A
DILUTION_003_DH_117_050.fcs
Ungated
56848
Single EVs
97.8
FSC-H
FSC-A
DILUTION_003_DH_117_050.fcs
SCATTER
51485
CD63+
73.4
SSC-A
Comp-R C-A
DILUTION_003_DH_117_050.fcs
Single EVs
50341
CD11B+
16.1
SSC-A
Comp-B E-A
DILUTION_003_DH_117_050.fcs
CD63+
36971
FTH1+
98.2
SSC-A
Comp-YG D-A
DILUTION_003_DH_117_050.fcs
CD11B+
5935
CD63+H
75.0
Count
Comp-R C-A
DILUTION_003_DH_117_050.fcs
Single EVs
50341
CD11B+H
17.0
Count
Comp-B E-A
DILUTION_003_DH_117_050.fcs
CD63+H
37734
FTH1+H
97.8
Count
Comp-YG D-A
DILUTION_003_DH_117_050.fcs
CD11B+H
6432

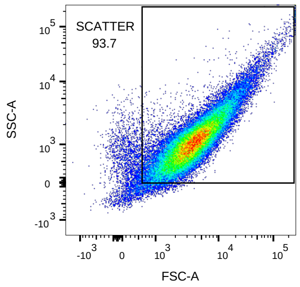

DILUTION_003_DH_125_051.fcs
Ungated
55606

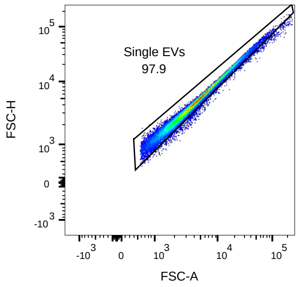

DILUTION_003_DH_125_051.fcs
SCATTER
52125

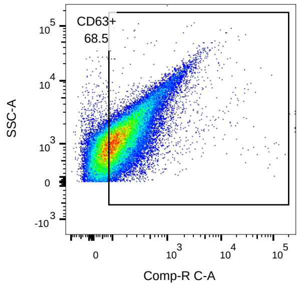

DILUTION_003_DH_125_051.fcs
Single EVs
51047

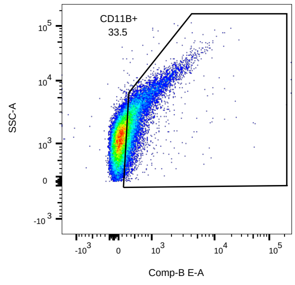

DILUTION_003_DH_125_051.fcs
CD63+
34962

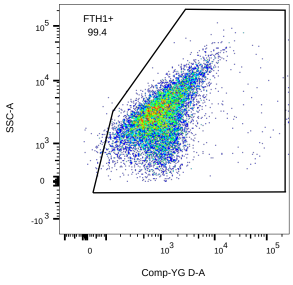

DILUTION_003_DH_125_051.fcs
CD11B+
11720

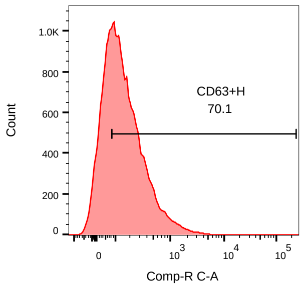

DILUTION_003_DH_125_051.fcs
Single EVs
51047

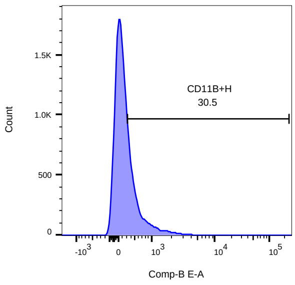

DILUTION_003_DH_125_051.fcs
CD63+H
35778

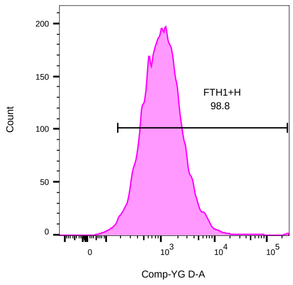

DILUTION_003_DH_125_051.fcs
CD11B+H
10899

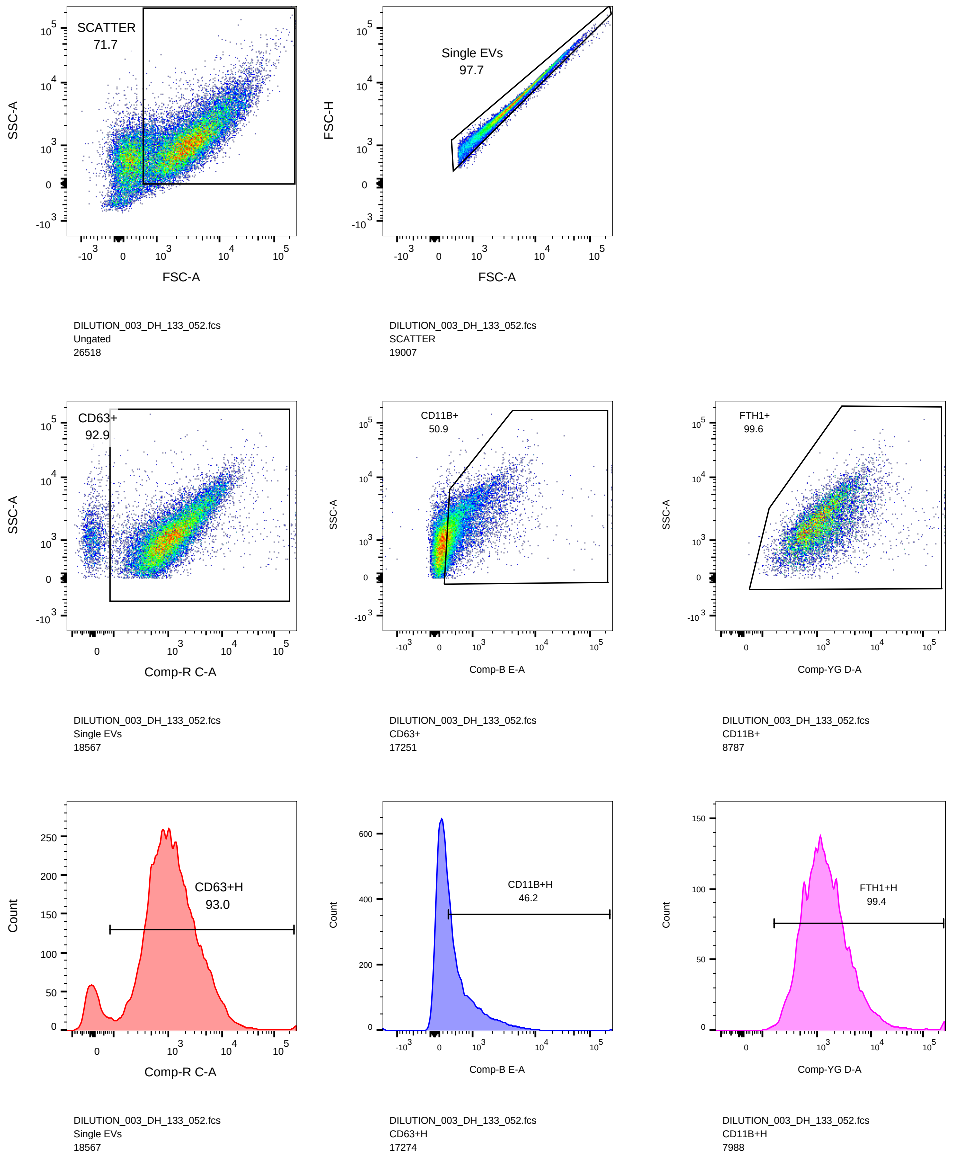
SCATTER
71.7
SSC-A
FSC-A
DILUTION_003_DH_133_052.fcs
Ungated
26518
Single EVs
97.7
FSC-H
FSC-A
DILUTION_003_DH_133_052.fcs
SCATTER
19007
CD63+
92.9
SSC-A
Comp-R C-A
DILUTION_003_DH_133_052.fcs
Single EVs
18567
CD11B+
50.9
SSC-A
Comp-B E-A
DILUTION_003_DH_133_052.fcs
CD63+
17251
FTH1+
99.6
SSC-A
Comp-YG D-A
DILUTION_003_DH_133_052.fcs
CD11B+
8787
CD63+H
93.0
Count
Comp-R C-A
DILUTION_003_DH_133_052.fcs
Single EVs
18567
CD11B+H
46.2
Count
Comp-B E-A
DILUTION_003_DH_133_052.fcs
CD63+H
17274
FTH1+H
99.4
Count
Comp-YG D-A
DILUTION_003_DH_133_052.fcs
CD11B+H
7988

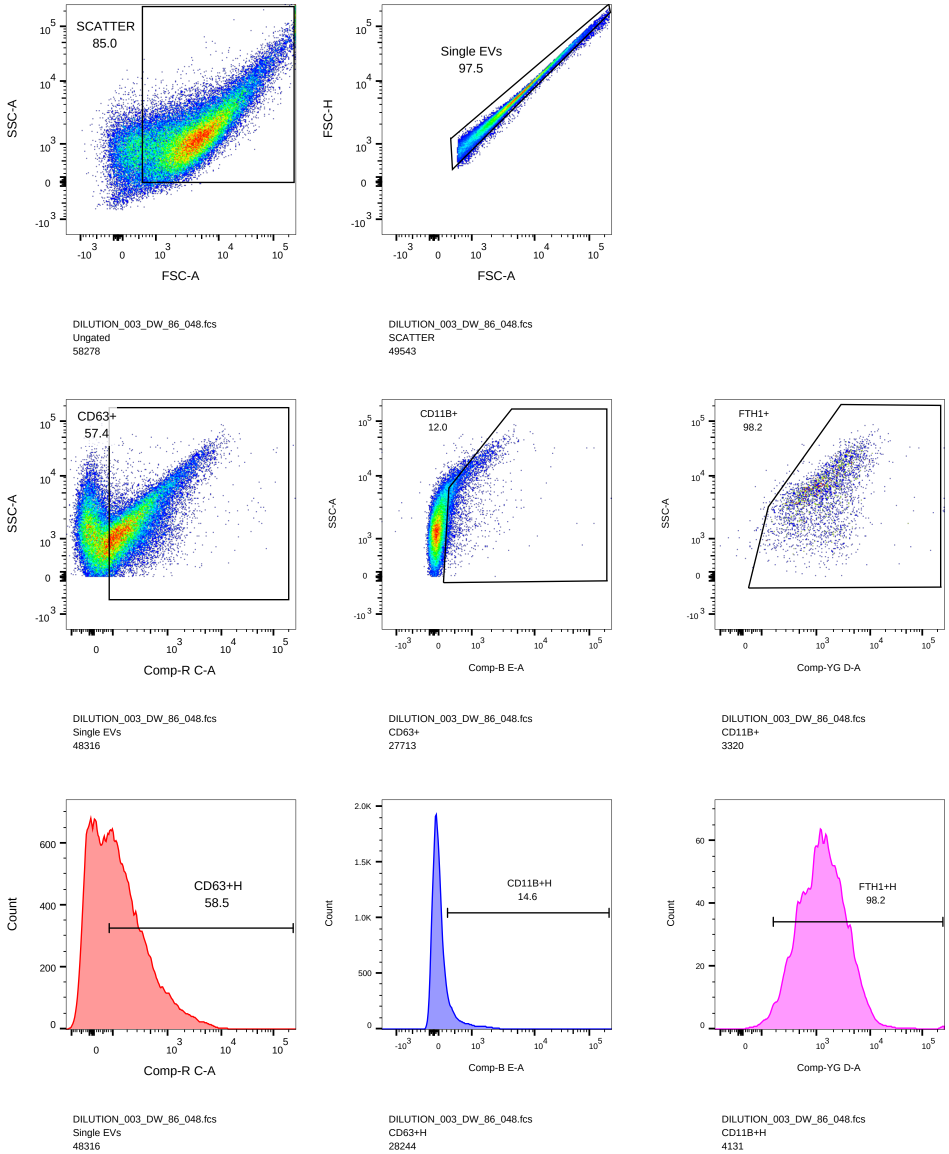
SCATTER
85.0
SSC-A
FSC-A
DILUTION_003_DW_86_048.fcs
Ungated
58278
Single EVs
97.5
FSC-H
FSC-A
DILUTION_003_DW_86_048.fcs
SCATTER
49543
CD63+
57.4
SSC-A
Comp-R C-A
DILUTION_003_DW_86_048.fcs
Single EVs
48316
CD11B+
12.0
SSC-A
Comp-B E-A
DILUTION_003_DW_86_048.fcs
CD63+
27713
FTH1+
98.2
SSC-A
Comp-YG D-A
DILUTION_003_DW_86_048.fcs
CD11B+
3320
CD63+H
58.5
Count
Comp-R C-A
DILUTION_003_DW_86_048.fcs
Single EVs
48316
CD11B+H
14.6
Count
Comp-B E-A
DILUTION_003_DW_86_048.fcs
CD63+H
28244
FTH1+H
98.2
Count
Comp-YG D-A
DILUTION_003_DW_86_048.fcs
CD11B+H
4131

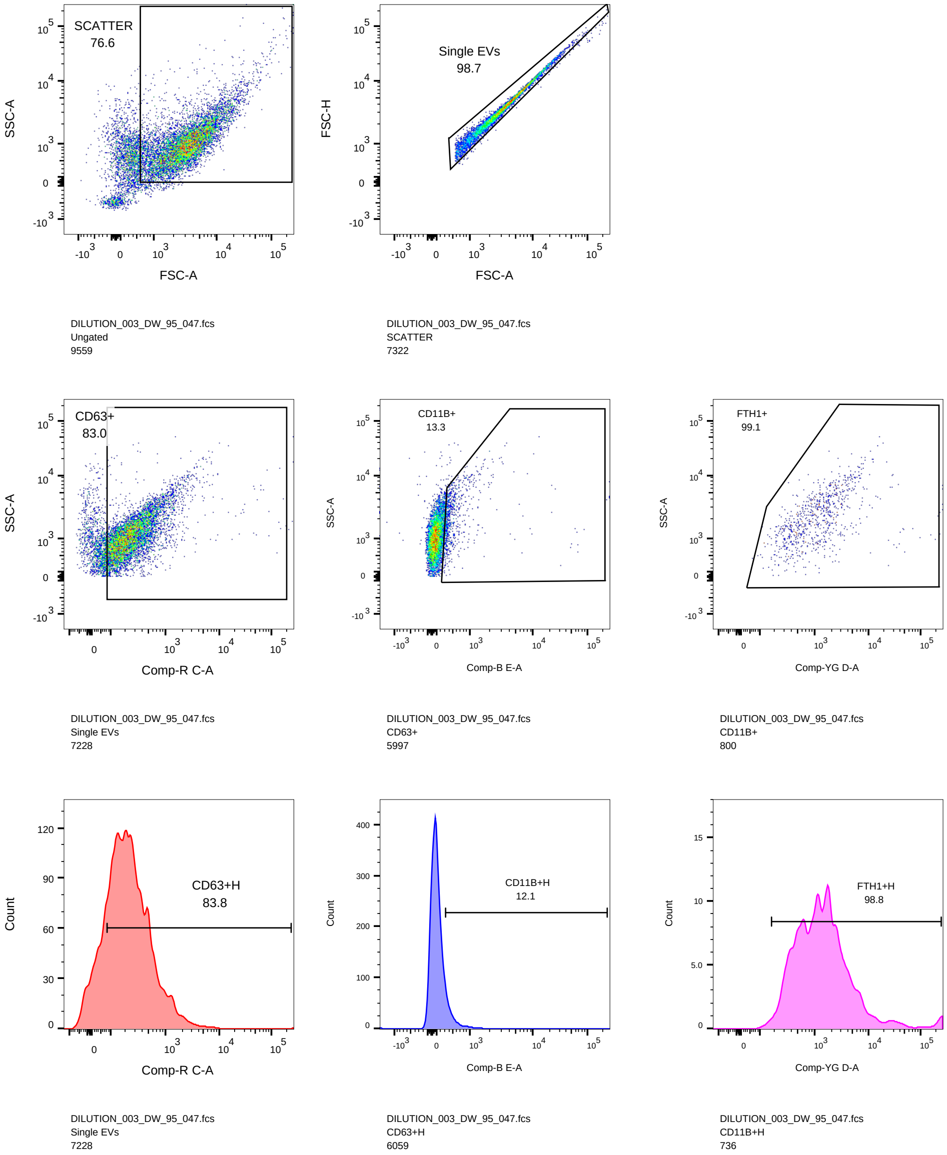
SCATTER
76.6
SSC-A
FSC-A
DILUTION_003_DW_95_047.fcs
Ungated
9559
Single EVs
98.7
FSC-H
FSC-A
DILUTION_003_DW_95_047.fcs
SCATTER
7322
CD63+
83.0
SSC-A
Comp-R C-A
DILUTION_003_DW_95_047.fcs
Single EVs
7228
CD11B+
13.3
SSC-A
Comp-B E-A
DILUTION_003_DW_95_047.fcs
CD63+
5997
FTH1+
99.1
SSC-A
Comp-YG D-A
DILUTION_003_DW_95_047.fcs
CD11B+
800
CD63+H
83.8
Count
Comp-R C-A
DILUTION_003_DW_95_047.fcs
Single EVs
7228
CD11B+H
12.1
Count
Comp-B E-A
DILUTION_003_DW_95_047.fcs
CD63+H
6059
FTH1+H
98.8
Count
Comp-YG D-A
DILUTION_003_DW_95_047.fcs
CD11B+H
736

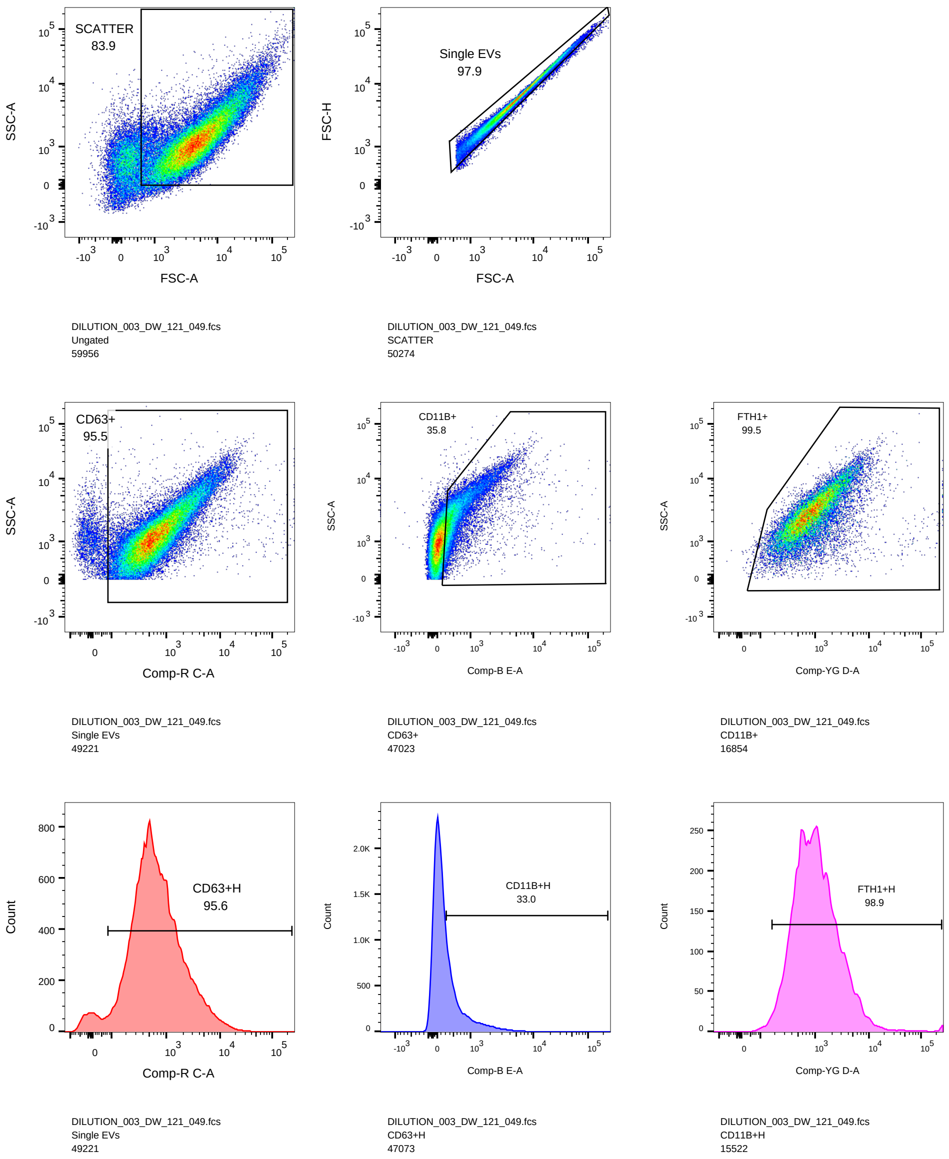
SCATTER
83.9
SSC-A
FSC-A
DILUTION_003_DW_121_049.fcs
Ungated
59956
Single EVs
97.9
FSC-H
FSC-A
DILUTION_003_DW_121_049.fcs
SCATTER
50274
CD63+
95.5
SSC-A
Comp-R C-A
DILUTION_003_DW_121_049.fcs
Single EVs
49221
CD11B+
35.8
SSC-A
Comp-B E-A
DILUTION_003_DW_121_049.fcs
CD63+
47023
FTH1+
99.5
SSC-A
Comp-YG D-A
DILUTION_003_DW_121_049.fcs
CD11B+
16854
CD63+H
95.6
Count
Comp-R C-A
DILUTION_003_DW_121_049.fcs
Single EVs
49221
CD11B+H
33.0
Count
Comp-B E-A
DILUTION_003_DW_121_049.fcs
CD63+H
47073
FTH1+H
98.9
Count
Comp-YG D-A
DILUTION_003_DW_121_049.fcs
CD11B+H
15522

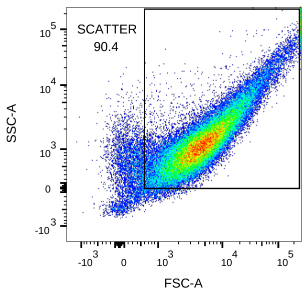

DILUTION_004_DH_117_056.fcs
Ungated
56570

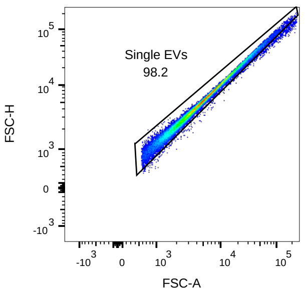

DILUTION_004_DH_117_056.fcs
SCATTER
51134

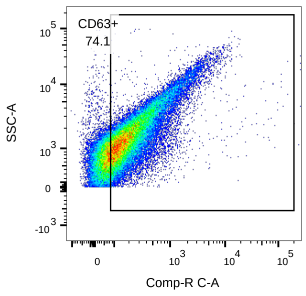

DILUTION_004_DH_117_056.fcs
Single EVs
50220

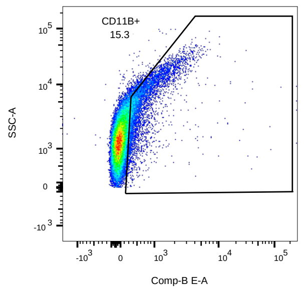

DILUTION_004_DH_117_056.fcs
CD63+
37202

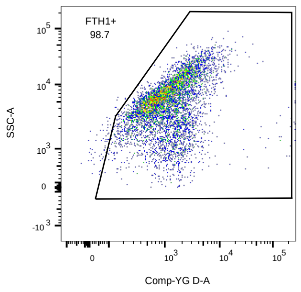

DILUTION_004_DH_117_056.fcs
CD11B+
5702

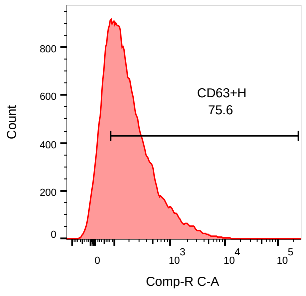

DILUTION_004_DH_117_056.fcs
Single EVs
50220

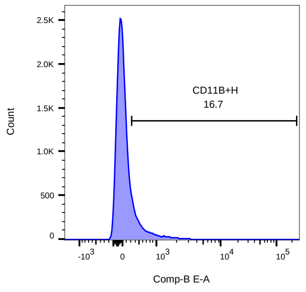

DILUTION_004_DH_117_056.fcs
CD63+H
37981

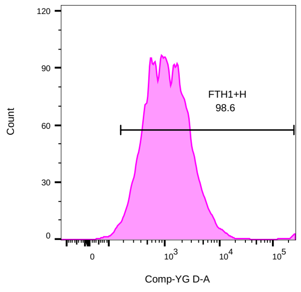

DILUTION_004_DH_117_056.fcs
CD11B+H
6344

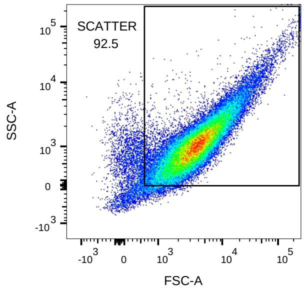

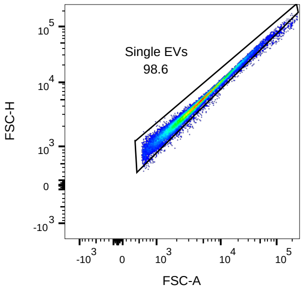

DILUTION_004_DH_125_057.fcs
Ungated
56147

DILUTION_004_DH_125_057.fcs
SCATTER
51908

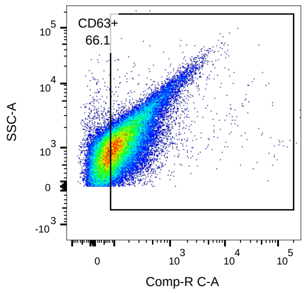

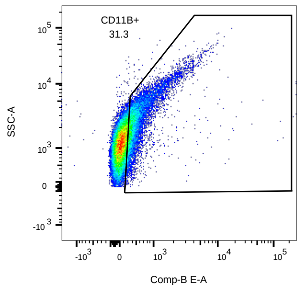

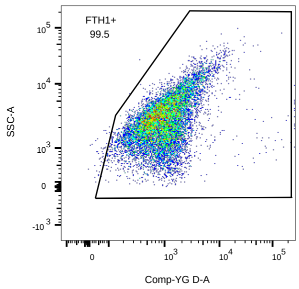

DILUTION_004_DH_125_057.fcs
Single EVs
51198

DILUTION_004_DH_125_057.fcs
CD63+
33821

DILUTION_004_DH_125_057.fcs
CD11B+
10587

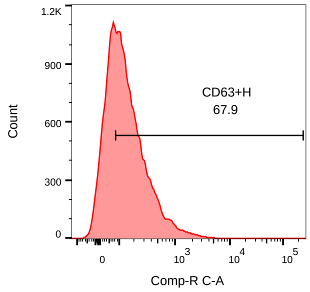

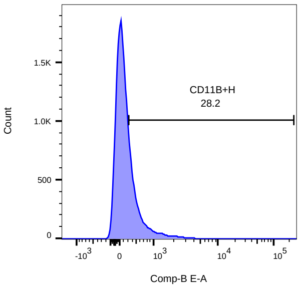

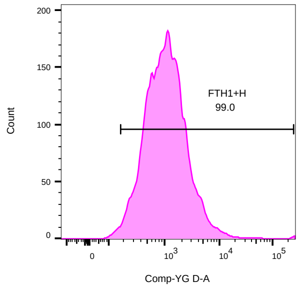

DILUTION_004_DH_125_057.fcs
Single EVs
51198

DILUTION_004_DH_125_057.fcs
CD63+H
34745

DILUTION_004_DH_125_057.fcs
CD11B+H
9815

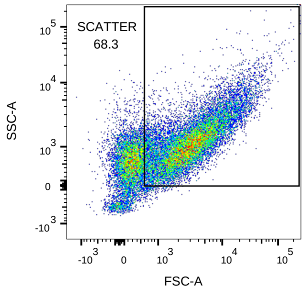

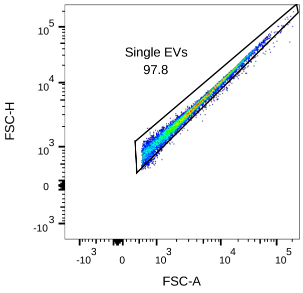

DILUTION_004_DH_133_058.fcs
Ungated
20828

DILUTION_004_DH_133_058.fcs
SCATTER
14233

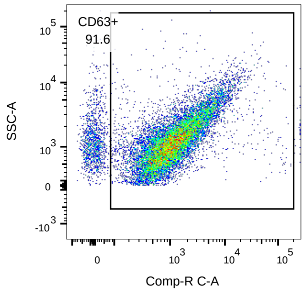

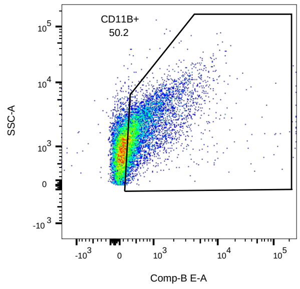

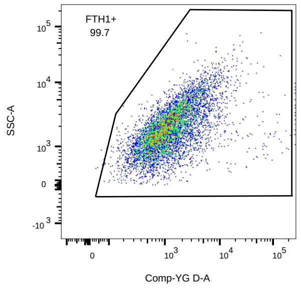

DILUTION_004_DH_133_058.fcs
Single EVs
13913

DILUTION_004_DH_133_058.fcs
CD63+
12746

DILUTION_004_DH_133_058.fcs
CD11B+
6398

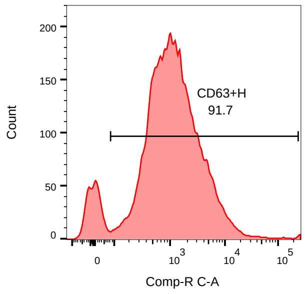

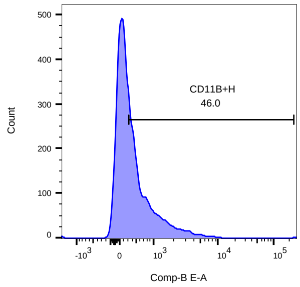

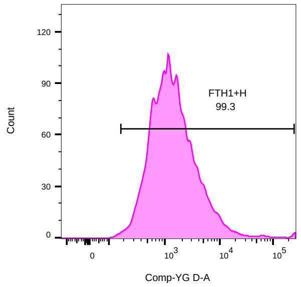

DILUTION_004_DH_133_058.fcs
Single EVs
13913

DILUTION_004_DH_133_058.fcs
CD63+H
12754

DILUTION_004_DH_133_058.fcs
CD11B+H
5866

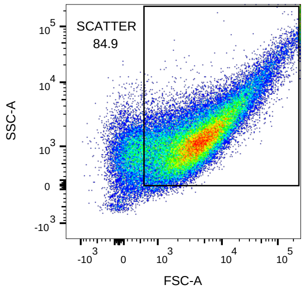

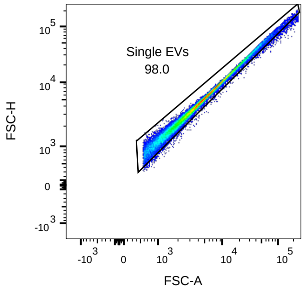

DILUTION_004_DW_86_054.fcs
Ungated
58132

DILUTION_004_DW_86_054.fcs
SCATTER
49383

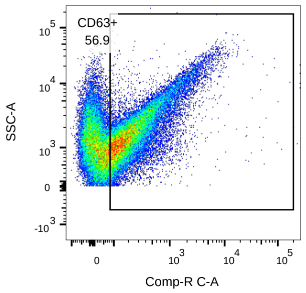

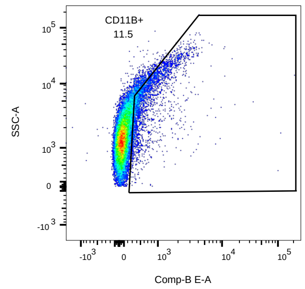

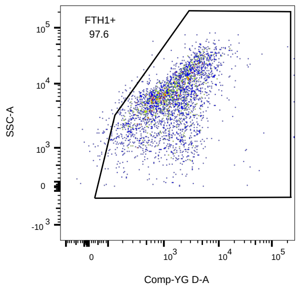

DILUTION_004_DW_86_054.fcs
Single EVs
48381

DILUTION_004_DW_86_054.fcs
CD63+
27537

DILUTION_004_DW_86_054.fcs
CD11B+
3154

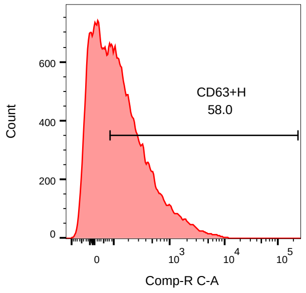

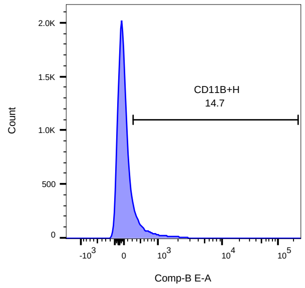

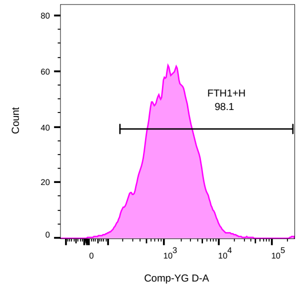

DILUTION_004_DW_86_054.fcs
Single EVs
48381

DILUTION_004_DW_86_054.fcs
CD63+H
28071

DILUTION_004_DW_86_054.fcs
CD11B+H
4120

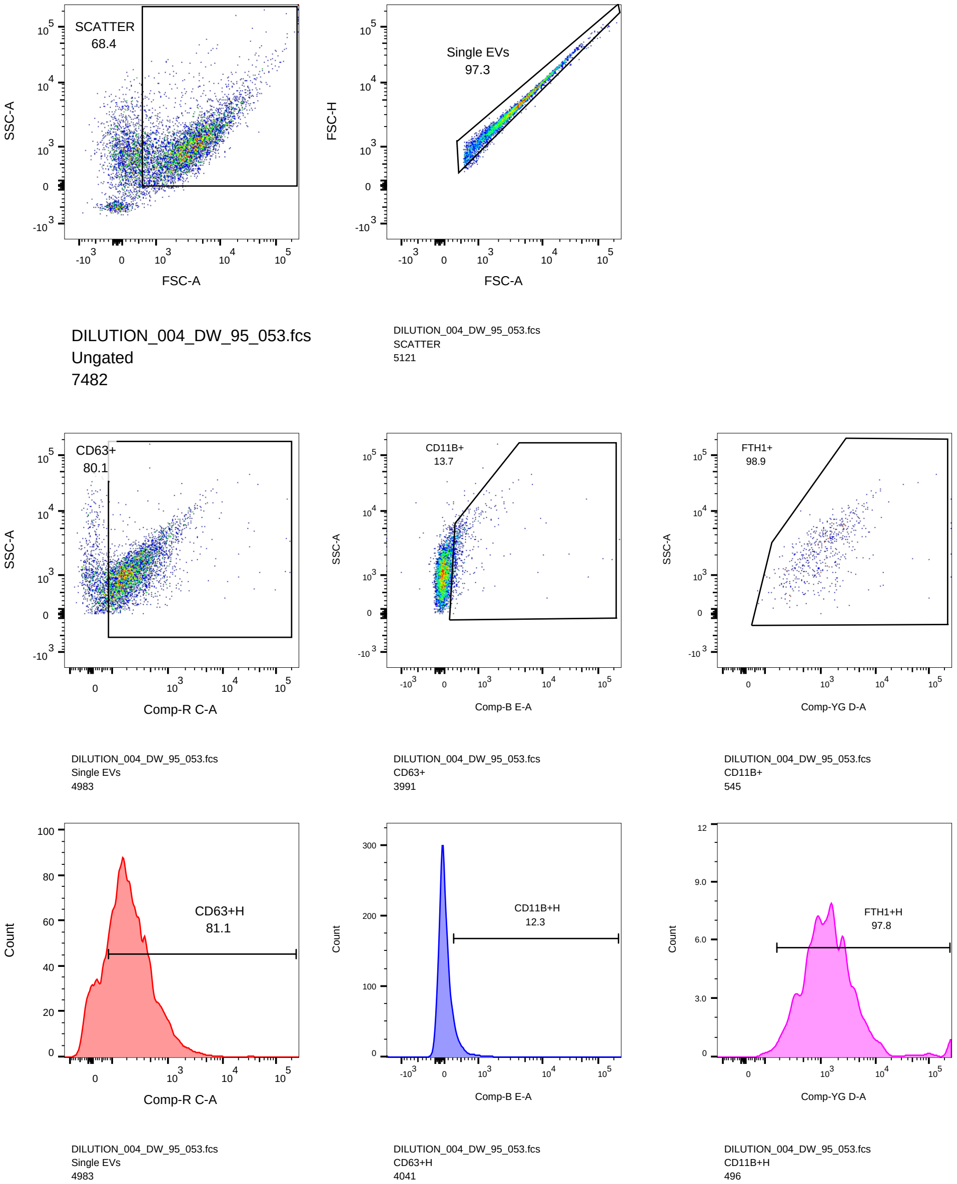
SCATTER
68.4
SSC-A
FSC-A
Single EVs
97.3
FSC-H
FSC-A
DILUTION_004_DW_95_053.fcs
Ungated
7482
DILUTION_004_DW_95_053.fcs
SCATTER
5121
CD63+
80.1
SSC-A
Comp-R C-A
CD11B+
13.7
SSC-A
Comp-B E-A
FTH1+
98.9
SSC-A
Comp-YG D-A
DILUTION_004_DW_95_053.fcs
Single EVs
4983
DILUTION_004_DW_95_053.fcs
CD63+
3991
DILUTION_004_DW_95_053.fcs
CD11B+
545
Count
CD63+H
81.1
Comp-R C-A
Count
CD11B+H
12.3
Comp-B E-A
Count
FTH1+H
97.8
Comp-YG D-A
DILUTION_004_DW_95_053.fcs
Single EVs
4983
DILUTION_004_DW_95_053.fcs
CD63+H
4041
DILUTION_004_DW_95_053.fcs
CD11B+H
496

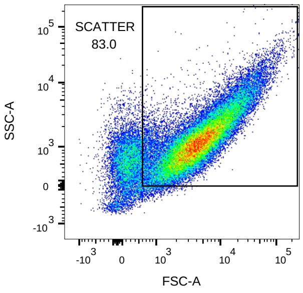

DILUTION_004_DW_121_055.fcs
Ungated
48319

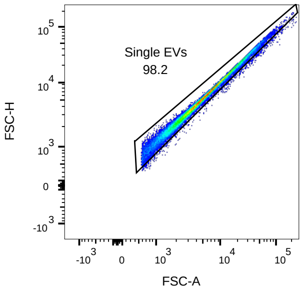

DILUTION_004_DW_121_055.fcs
SCATTER
40105

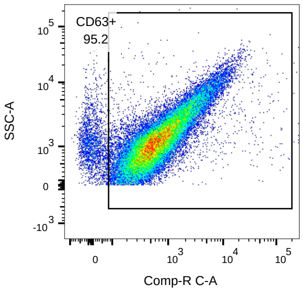

DILUTION_004_DW_121_055.fcs
Single EVs
39375

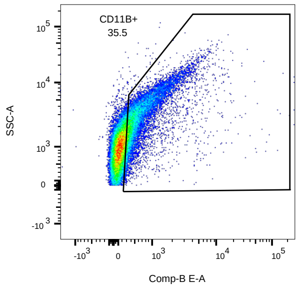

DILUTION_004_DW_121_055.fcs
CD63+
37473

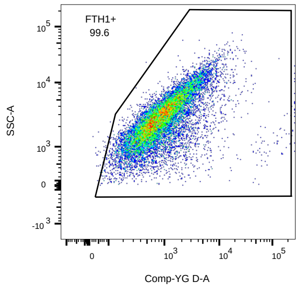

DILUTION_004_DW_121_055.fcs
CD11B+
13300

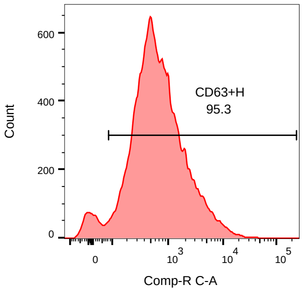

DILUTION_004_DW_121_055.fcs
Single EVs
39375

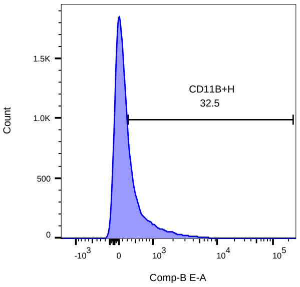

DILUTION_004_DW_121_055.fcs
CD63+H
37521

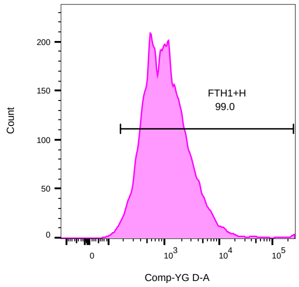

DILUTION_004_DW_121_055.fcs
CD11B+H
12190
